# Derivation of a Human In Vivo Benchmark Dose for Bisphenol A from ToxCast In Vitro Concentration Response Data Using a Computational Workflow for Probabilistic Quantitative *In Vitro* to *In Vivo* Extrapolation

**DOI:** 10.3389/fphar.2021.754408

**Published:** 2022-02-11

**Authors:** George Loizou, Kevin McNally, Alicia Paini, Alex Hogg

**Affiliations:** ^1^ Health and Safety Executive, Harpur Hill, Buxton, United Kingdom; ^2^ European Commission Joint Research Centre, Ispra, Italy

**Keywords:** PBK, in silico, *in vitro*, QIVIVE, BPA (bisphenol A)

## Abstract

A computational workflow which integrates physiologically based kinetic (PBK) modelling; global sensitivity analysis (GSA), Approximate Bayesian Computation (ABC), Markov Chain Monte Carlo (MCMC) simulation and the Virtual Cell Based Assay (VCBA) for the estimation of the active, free *in vitro* concentration of chemical in the reaction medium was developed to facilitate quantitative *in vitro* to *in vivo* extrapolation (QIVIVE). The workflow was designed to estimate parameter and model uncertainty within a computationally efficient framework. The workflow was tested using a human PBK model for bisphenol A (BPA) and high throughput screening (HTS) *in vitro* concentration-response data, for estrogen and pregnane X receptor activation determined in human liver and kidney cell lines, from the ToxCast/Tox21 database. *In vivo* benchmark dose 10% lower confidence limits (BMDL_10_) for oral uptake of BPA (ng/kg BW/day) were calculated from the *in vivo* dose-responses and compared to the human equivalent dose (HED) BMDL_10_ for relative kidney weight change in the mouse derived by European Food Safety Authority (EFSA). Three from four *in vivo* BMDL_10_ values calculated in this study were similar to the EFSA values whereas the fourth was much smaller. The derivation of an uncertainty factor (UF) to accommodate the uncertainties associated with measurements using human cell lines *in vitro*, extrapolated to *in vivo*, could be useful for the derivation of Health Based Guidance Values (HBGV).

## 1 Introduction

The modern ecosystem is replete with chemicals of anthropogenic and natural origin. These chemicals are both ubiquitous and diverse and present a considerable health risk assessment challenge. People are exposed in their homes, workplaces, by the use of pharmaceuticals, cosmetics and cleaning products and from the contamination of food. Anthropogenic contaminants found in our food include pesticides, biocides, food and feed additives, pharmaceuticals, air pollutants, persistent organic pollutants, heavy metals, perfluoroalkyl substances, brominated flame retardants, dioxins etc., and those of natural origin (marine biotoxins, mycotoxins etc.).

In the risk assessment of a given chemical, a “critical” toxicity study is one in which a key apical endpoint that is, an observable clinical outcome or pathology indicative of a disease resulting from exposure to the chemical, is identified based on the elicitation and dose-response relationship of an adverse effect in animal studies. Such a dose or concentration, known as the reference point (RP) or point of departure (PoD), is defined as the point on a toxicological dose–response curve established from experimental data that corresponds to an estimated no-observed-adverse-effect-level (NOAEL), lowest-observed-adverse- effect-level (LOAEL) or preferably, a benchmark dose (BMD). PoDs are used as the basis for the derivation of safe levels of human exposure known as Health Based Guidance Values (HBGV) (Ingenbleek L et al., 2021).

The most common RPs or PoDs are the NOAEL and the BMD. The NOAEL approach uses statistical methods to identify the test dose that has no significant effect compared to the control group. The BMD approach, however, fits a dose–response model(s) to a complete dose–response dataset to identify the benchmark dose lower and upper confidence limits (BMDL and BMDU) for a selected observed level of effect, the benchmark response (BMR) (e.g., a 5% response). The BMD is increasingly preferred by regulatory agencies, but its use is often limited by test design (Bokkers and Slob 2005, 2007; European Food Safety 2009; EFSA Scientific Committee et al., 2017b).

However, the reliance on toxicological studies, conducted using laboratory animals for the derivation of RPs and HBGVs, is being challenged. The international scientific community has been involved in considerable research and validation efforts to reduce animal testing and provide alternative-to-animal testing methods. These are known as new approach methods (NAMs).

Alternative-to-animal methods invariably refer to an *in vitro* bioassay based strategy that ideally uses human cell lines for the determination of a RP. Invariably, *in vitro* concentration-response data must be converted to *in vivo* dose-responses to be used in human safety testing of chemicals. This activity is known as quantitative *in vitro* to *in vivo* extrapolation (QIVIVE) (Yoon et al., 2012; Bale et al., 2014; Yoon et al., 2015). Examples of QIVIVE increasingly involve the application of physiologically-based kinetic (PBK) modelling-based reverse dosimetry for the translation of *in vitro* to *in vivo* responses and the derivation of *in vivo* BMDs (Louisse et al., 2010; Louisse et al., 2012; Strikwold et al., 2013; Louisse et al., 2016; Boonpawa et al., 2017a, 2017b; Li et al., 2017; Punt et al., 2017; Strikwold et al., 2017; Adam et al., 2019; Zhao et al., 2019; Shi et al., 2020; Zhang et al., 2020). In these studies, all parameters, other than input dose or exposure, are held fixed at central values. An optimisation routine is implemented to minimise the discrepancy between a target *in vivo* concentration, predicted by the PBK model, and a given *in vitro* concentration. The dose concentration which corresponds to the target *in vitro* concentration, is considered a surrogate for the *in vivo* concentration. However, these studies did not account for structural uncertainty in the PBK model nor parameter value uncertainty. It is known that the amount of biological mechanistic detail described in a PBK model could have a bearing on model output (Rowland et al., 2017). Also, understanding and quantifying the level of uncertainty in each step of a chemical safety assessment with NAMs is important for the development of confidence in this approach (Berggren et al., 2017).

Another limitation of past QIVIVE studies was the use of applied or nominal *in vitro* concentrations only, that is, no consideration was made of the fate and distribution of the chemical in the reaction vessel. This could be a significant omission because the concentrations of chemical available for interaction with sub-cellular protein receptors and enzymes could be substantially lower than the nominal concentration (Tanneberger et al., 2010; Groothuis et al., 2015; Kramer et al., 2015; Proença et al., 2021). Chemicals have different properties, for instance highly lipophilic chemicals can interact with constituents of the reaction medium as well as reaction vessel geometry and composition. For example, the chemical can migrate and bind to the plastic of the reaction vessel (Proença et al., 2019; Proença et al., 2021). To account for this one can set up *in vitro* distribution and fate measurements or use in silico tools to predict distribution. Currently, there are several mathematical models that allow calculation of the proportion of the nominal concentration that is free and presumably active and therefore available in the reaction medium to be taken up by the cell (Tanneberger et al., 2010; Armitage et al., 2014; Comenges et al., 2017; Fischer et al., 2017; Fisher et al., 2019). One such model is the Virtual Cell Based Assay (VCBA). This is an algorithm which integrates models for: 1) chemical fate and transport; 2) cell partitioning; 3) cell growth and division; 4) toxicity and effects; and 5) considering the experimental set up (size of well-plate). Ultimately, the VCBA can simulate the active, free concentration of chemical in the medium, which can be used on its own to design and interpret *in vitro* experiments, and in combination with PBK models to perform *in vitro* to *in vivo* extrapolation (Comenges et al., 2017; Bell et al., 2018).

In order to address the issues of PBK model structure uncertainty, parameter value uncertainty and the calculation of free concentration of chemical *in vitro*, the VCBA was used in combination with an algorithm, described in detail previously, which was developed to extrapolate *in vitro* concentration-response to *in vivo* dose-response relationships (McNally and Loizou 2015; McNally et al., 2018; Loizou et al., 2021). The latter applies a rigorous statistical framework for the accommodation of uncertainty in both PBK model parameters, the quality of fit to measured biological monitoring data, and a consideration of how this affects an *in vivo* dose response relationship in the context of QIVIVE (Judson et al., 2011).

The workflow uses global sensitivity analysis (GSA), PBK modelling, Approximate Bayesian Computation (ABC) and Markov Chain Monte Carlo (MCMC) simulation to convert *in vitro* concentration-response data to *in vivo* dose-response data (McNally et al., 2018; Loizou et al., 2021). There are several advantages regarding exposure or dose reconstruction provided by this probabilistic approach. Firstly, defining informative prior distributions around parameters converts a deterministic model to a population model which can account for inter-individual variability. Secondly, the application of GSA is appropriate for systems where tissue dose is not necessarily linearly related to external exposure. Finally, this combination can extract population variability and multiple routes of exposure information integrated within pharmacokinetic data (McNally et al., 2012; McNally and Loizou 2015; McNally et al., 2018; Loizou et al., 2021). In this report we present the results from a study investigating the QIVIVE of bisphenol A (BPA) (4,4'-(propane-2,2-diyl) diphenol).

As emphasised in our previous studies the purpose of this study was not to propose an animal-free risk assessment for BPA, since it is recognised that much work is still needed to demonstrate *in vitro* to *in vivo* concordance for systemic, chronic exposures to environmental xenobiotics (McNally and Loizou 2015; McNally et al., 2018; Loizou et al., 2021). Therefore, the selection of *in vitro* concentration-response data for use in this study was not predicated on seeking consistency with the apical endpoint used to calculate a BMDL_10_ by a regulatory agency, in this case EFSA, or whether the data were consistent with an adverse outcome pathway (AOL) for BPA. Our purpose was to investigate the behaviour of BPA, as a structurally dissimilar chemical to perfluorooctanoic acid (PFOA) (Loizou, et al., 2021) and ethylene glycol monoethyl ether (EGME) (McNally, et al., 2018) studied previously and to demonstrate the utility of: 1) freely available concentration-response data, 2) the importance of using free *in vitro* concentrations, and 3) a computational workflow which quantifies uncertainty and variability by integrating PBK modelling, the VCBA, GSA, ABC and MCMC simulation for QIVIVE.

## 2 Materials and Methods

### 2.1 PBK Model

The biokinetics of bisphenol A following single oral doses were described using a PBK model previously developed for the plasticizer DPHP (McNally et al., 2021), with adaptions to model structure made, as necessary. The final model described entry of BPA through ingestion with absorption of BPA from the stomach and intestines and a simple model of the lymphatic system describing uptake of BPA via the lacteals in the small intestine and entering venous blood after bypassing the liver. The dose that entered the lymphatic system was coded as a fraction of the administered dose; a fraction of administered dose was coded as entering the liver via the portal vein; a fraction of the administered dose of BPA passed through the intestine without being absorbed and was coded as administered dose minus the lymphatic and hepatic dose components. The model described the metabolism of BPA-to-BPA glucuronide (BPAG) and BPA sulphate (BPAS) in both liver and gut. Sub-models were included to describe the kinetics of BPAG and BPAS, with the models for BPA and the two metabolites connected via gut and liver. Binding of BPA, BPAG and BPAS was coded from arterial blood, with the consequence that only the unbound fraction in blood was available for distribution to organs and tissues, metabolism, and elimination. However, the bound and unbound fractions of BPA in blood are in equilibrium such that the bound fraction is gradually removed to become unbound as the existing unbound fraction is excreted.

The model structures for BPA and the two metabolites differed only in the coding of uptake required for BPA (including the simplified description of a lymphatic compartment). BPA and metabolite models had a stomach and intestine draining into the liver. Adipose, blood, kidney, and slowly and rapidly perfused compartments were included. Elimination of BPA, BPAG and BPAS was coded through the kidney compartment, with first order elimination rates, proportional to kidney tissue concentration, coded in each case. Both BPA and metabolite models included the transport process of enterohepatic recirculation. Uptake of BPA and metabolites from the liver into bile was modelled as a first order uptake process with a delay of 4 hours (to represent transport in bile) before BPA (and metabolites) appeared in the small intestine and were available for reabsorption. First order elimination rates for each substance were coded to account for fractions of recirculated BPA and metabolites that were eliminated in faeces rather than reabsorbed from the small intestine. As a consequence of coding enterohepatic recirculation, the PBPK model was solved as a system of delay differential equations (DDEs).

**FIGURE 1:**
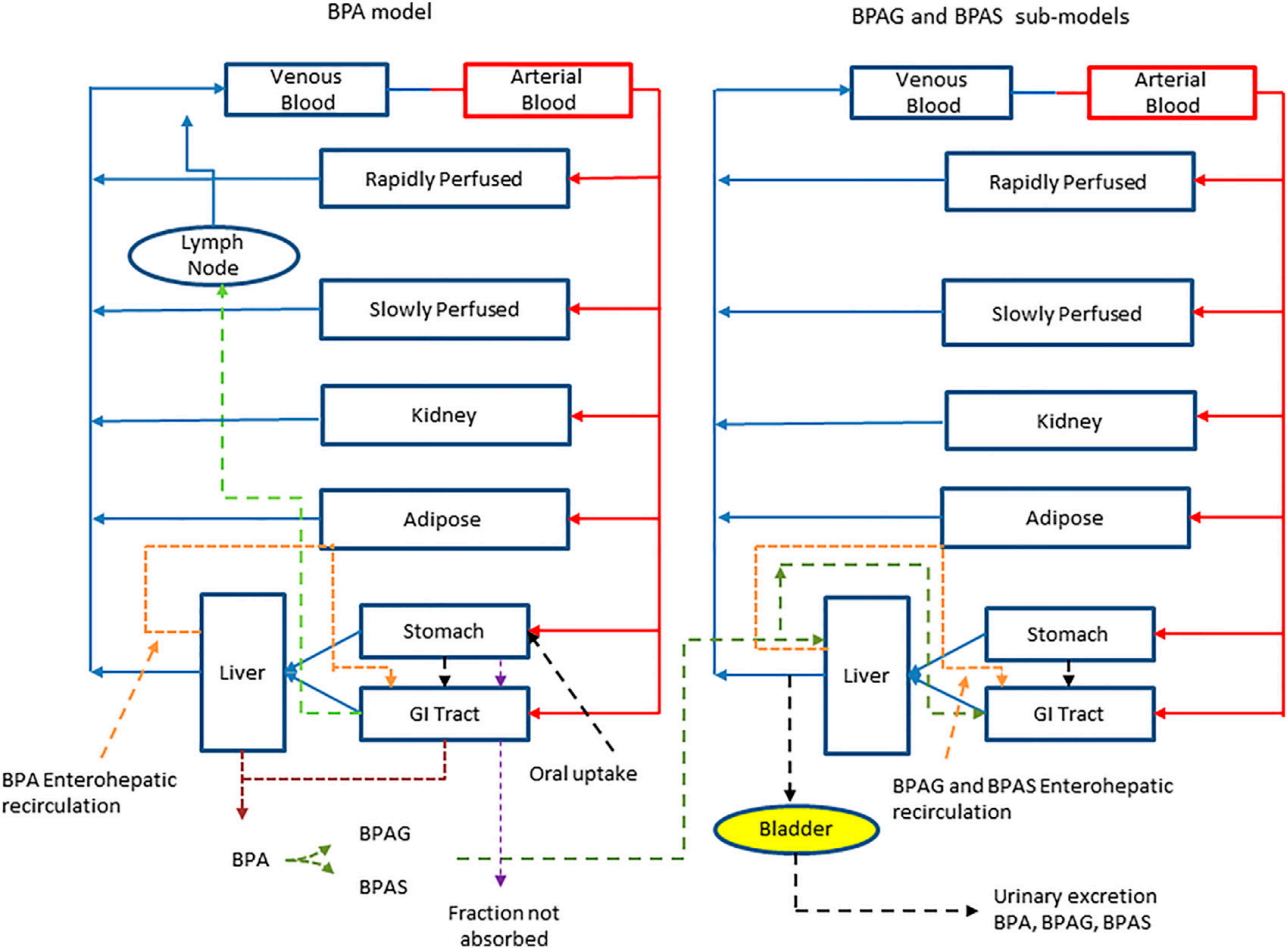
A schematic of the model for BPA and sub-model for BPAG and BPAS. The main model contained a lymphatic compartment (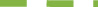) which received a portion of the oral dose of BPA from the stomach and GI tract which entered the systemic circulation after bypassing the liver. The model described metabolism of BPA to BPAG and BPAS in the gut with subsequent uptake into the hepatic portal vein as well as hepatic metabolism of BPA to BPAG and BPAS. Enterohepatic recirculation of BPA, BPAG and BPAS was also included.

The final structure of the model described above followed the iterative model development process (incorporating uncertainty and sensitivity analysis) documented in McNally et al. (2021). Table 1 lists a glossary of parameters name and abbreviations. A schematic representation of the model is shown in Figure 1.

**TABLE 1:** Model Parameters

Physiological parameters	Abbreviation
Body weight	BW
Tissue volumes (Fraction of BW)	
Liver	VliC
Stomach	VstC
Gut	VguC
Kidney	VkiC
Lymph	VlymphC
Fat	VfaC
Slowly perfused	VspdC
Rapidly perfused	VrpdC
Blood	VBldC
Cardiac output	QCC
Blood flows (fraction CO)	
Liver	QhepartC
Stomach	QstC
Gut	QguC
Kidney	QkiC
Fat	QfaC
Slowly perfused	QspdC
Rapidly perfused	QrpdC
Fraction of dose taken up into liver	FracDOSEHep
Fraction of dose taken up into lymph	FracDOSELymph
Time taken to drink or eat	DRINKTIME
Fraction of BPA bound to plasma proteins	FB_BPA
Fraction of BPAG bound to plasma proteins	FB_BPAG
Fraction of BPAS bound to plasma proteins	FB_BPAS
Hepatic microsomal protein yield	MPY
Gut microsomal protein yield	MPYgu
Rate Constants	
Stomach to hepatic portal permeability rate	BELLYPERM
Gut to hepatic portal permeability rate	GIPERM
Stomach to lymph permeability rate	BELLYPERMLymph
Gut to lymph portal permeability rate	GIPERMLymph
Maximum emptying rate from stomach	KEMAX
Minimum emptying rate from stomach	KEMIN
BPA gut to bowel elimination rate	K1_BPA_GUT
BPAG gut to bowel elimination rate	K1_BPAG_GUT
BPAS gut to bowel elimination rate	K1_BPAS_GUT
BPA liver to bile elimination rate	K1_BPA_LIVER
BPAG liver to bile elimination rate	K1_BPAG_LIVER
BPAS liver to bile elimination rate	K1_BPAS_LIVER
BPA urinary elimination rate	K1_BPA_Urine
BPAG urinary elimination rate	K1_BPAG_Urine
BPAS urinary elimination rate	K1_BPAS_Urine
BPA from lymph to blood elimination rate	K1Lymph
Metabolic Rate Constants	
In vitro liver maximum rate of metabolism BPA to BPAG	Vmax_liv_BPA_in_vitro
In vitro liver Michaelis Menten constant BPA to BPAG	KM_liv_BPA_in_vitro
In vitro gut maximum rate of metabolism BPA to BPAG	Vmax_liv_BPAG_in_vitro
In vitro gut Michaelis Menten constant BPA to BPAG	KM_liv_BPAG_in_vitro
In vitro liver maximum rate of metabolism BPA to BPAS	Vmax_liv_BPAS_in_vitro
In vitro liver Michaelis Menten constant	
BPA to BPAS	KM_liv_BPAS_in_vitro
In vitro gut maximum rate of metabolism BPA to BPAS	Vmax_gut_BPAS_in_vitro
In vitro gut Michaelis Menten constant BPA to BPAS	KM_gut_BPAS_in_vitro
Partition coefficients (tissue:blood)	
BPA red blood cells:plasma	Pbab
BPA liver:blood	Plib
BPA kidney:blood	Pkib
BPA fat:blood	Pfab
BPA gut:blood	Pgub
BPA stomach:blood	Pstb
BPA rapidly perfused:blood	Prpdb
BPA slowly perfused:blood	Pspdb
BPAG red blood cells:plasma	PbaG
BPAG liver:blood	PliG
BPAG kidney:blood	PkiG
BPAG fat:blood	PfaG
BPAG gut:blood	PguG
BPAG stomach:blood	PstG
BPAG rapidly perfused:blood	PrpdG
BPAG slowly perfused:blood	PspdG
BPAS red blood cells:plasma	PbaG
BPAS liver:blood	PliG
BPAS kidney:blood	PkiG
BPAS fat:blood	PfaG
BPAS gut:blood	PguG
BPAS stomach:blood	PstG
BPAS rapidly perfused:blood	PrpdG
BPAS slowly perfused:blood	PspdG

#### 2.1.1 Parameterisation

Baseline estimates of organ and tissue masses and regional blood flows were taken from Brown et al. (1997) and (ICRP 2002). The mass of the lymphatic system was obtained from Offman et al. (2016). The method of Schmitt (2008), which was developed to predict the tissue distribution of chemicals with an octanol: water partition coefficient, (Log Pow) <5.17 used to predict the PCs of BPA, BPAG and BPAS (Table 2).

**TABLE 2:** Default probability distributions (and upper and lower bounds) ascribed to PBPK model parameters.

**Parameter**	**Unit**	**Mean**	**SD**	**Lower bound**	**Upper bound**	**Distribution**
BW	Kg	4.36	0.313	3.747	4.973	Lognormal
VliC	L kg^−1^ BW	0.0307	0.00758	0.02	0.05	Normal
VstC	L kg^−1^ BW	0.0210	0.00069	0.021	0.0235	Normal
VguC	L kg^−1^ BW	0.0150	0.00234	0.008	0.0220	Normal
VkiC	L kg^−1^ BW	0.0038	0.00148	0.0012	0.005	Normal
VlymphC	L kg^−1^ BW	0.0036	0.0007	0.0022	0.0050	Normal
VfaC	L kg^−1^ BW	0.27	0.0600	0.1500	0.39	Normal
VspdC	L kg^−1^ BW	0.6050	0.1000	0.4500	0.7500	Normal
VrpdC	L kg^−1^ BW	0.0302	0.005	0.010	0.0454	Normal
VBldC	L kg^−1^ BW	0.060	0.01	0.04	0.09	Normal
QCC	L h^−1^ kg^−1^ BW^0.75^	11	1	9	13	Normal
QhepartC	Unit less	0.0690	0.0060	0.03	0.12	Normal
QstC	Unit less	0.0110	0.0009	0.005	0.015	Normal
QguC	Unit less	0.1490	0.0130	0.09	0.25	Normal
QkiC	Unit less	0.20	0.05	0.10	0.30	Normal
QfaC	Unit less	0.0500	0.0050	0.0300	0.0699	Normal
QspdC	Unit less	0.2870	0.0221	0.2100	0.3600	Normal
QrpdC	Unit less	0.2100	0.0168	0.1600	0.2700	Normal
FracDOSEHep	Unit less	-	-	0.7	0.92	Uniform
FracDOSELymph	Unit less	-	-	0.02	0.08	Uniform
FB_BPA	Unit less	-	-	0	0.99	Uniform
FB_BPAG	Unit less	-	-	0.7	0.99	Uniform
FB_BPAS	Unit less	-	-	0.7	0.99	Uniform
MPY	mg/g	34	10	14	54	Normal
MPYgu	mg/g	3.9	0.8	2.3	5.5	Normal
BELLYPERM	h^−1^	-	-	0.1	10	Uniform
GIPERM	h^−1^	-	-	0.5	25	Uniform
BELLYPERMLymph	h^−1^	-	-	0.84	2.5	Uniform
GIPERMLymph	h^−1^	-	-	0.55	1.6	Uniform
KEMAX	h^−1^	-	-	0.1	25	Uniform
KEMIN	h^−1^	-	-	0.0025	0.0075	Uniform
K1_BPA_GUT	h^−1^	-	-	0.01	20	Uniform
K1_BPAG_GUT	h^−1^	-	-	0.01	20	Uniform
K1_BPAS_GUT	h^−1^	-	-	0.01	20	Uniform
K1_BPA_LIVER	h^−1^	-	-	0.55	1.6	Uniform
K1_BPAG_LIVER	h^−1^	-	-	0.005	0.015	Uniform
K1_BPAS_LIVER	h^−1^	-	-	0.005	0.015	Uniform
K1_BPA_Urine	h^−1^	-	-	0.0005	0.0015	Uniform
K1_BPAG_Urine	h^−1^	-	-	0.0005	0.0015	Uniform
K1_BPAS_Urine	h^−1^	-	-	0.0005	0.0015	Uniform
K1_BPA_REMOVED_PLASMA	h^−1^		-	0.01	100	Uniform
K1_BPAG_REMOVED_PLASMA	h^−1^		-	10	150	Uniform
K1_BPAS_REMOVED_PLASMA	h^−1^		-	0.01	100	Uniform
K1Lymph	h^−1^	-	-	0.25	0.75	Uniform
Lymphlag	h^−1^	-	-	0.25	1.25	Uniform
Vmax_liv_BPA_in_vitro	pmol/min/mg	4255	900	1000	8000	Normal
KM_liv_BPA_in_vitro	mg/L	1.118	0.20	0.1	2.5	Normal
Vmax_liv_BPAS_in_vitro	pmol/min/mg	80	30	13	133	Normal
KM_liv_BPAS_in_vitro	mg/L	3.114	0.6	1.5	6.5	Normal
Vmax_gut_BPAG_in_vitro	pmol/min/mg	487	100	244	974	Normal
KM_gut_BPAG_in_vitro	mg/L	18.29	4	9	37	Normal
Vmax_gut_BPAS_in_vitro	pmol/min/mg	73	30	13	133	Normal
KM_gut_BPAS_in_vitro	mg/L	3.114	0.6	1.5	6.5	Normal
Pbab	Unit less	-	-	0.36	1.1	Uniform
Plib	Unit less	-	-	0.36	1.1	Uniform
Pkib	Unit less	-	-	1.35	15	Uniform
Pfab	Unit less	-	-	1.35	15	Uniform
Pgub	Unit less	-	-	1.35	15	Uniform
Pstb	Unit less	-	-	1.35	15	Uniform
Prpdb	Unit less	-	-	1.4	4.2	Uniform
Pspdb	Unit less	-	-	1.4	4.2	Uniform
PbaG	Unit less	-	-	0.7	2.1	Uniform
PliG	Unit less	-	-	1.0	23	Uniform
PkiG	Unit less	-	-	1.0	23	Uniform
PfaG	Unit less	-	-	1.2	3.60	Uniform
PguG	Unit less	-	-	1.0	23	Uniform
PstG	Unit less	-	-	1.70	5.3	Uniform
PrpdG	Unit less	-	-	2.1	6.4	Uniform
PspdG	Unit less	-	-	1	3	Uniform
PbaS	Unit less	-	-	0.7	2.1	Uniform
PliS	Unit less	-	-	1.0	23	Uniform
PkiS	Unit less	-	-	1.0	23	Uniform
PfaS	Unit less	-	-	1.3	3.9	Uniform
PguS	Unit less	-	-	1.0	23	Uniform
PstS	Unit less	-	-	1.9	5.7	Uniform
PrpdS	Unit less	-	-	2.3	6.8	Uniform
PspdS	Unit less	-	-	1	3.1	Uniform
Parameter	Unit	Mean	SD	Lower bound	Upper bound	Distribution
BW	Kg	4.36	0.313	3.747	4.973	Lognormal
VliC	L kg^−1^ BW	0.0350	0.00758	0.021	0.0490	Normal
VstC	L kg^−1^ BW	0.0210	0.00069	0.021	0.0235	Normal
VguC	L kg^−1^ BW	0.0150	0.00234	0.008	0.0220	Normal
VkiC	L kg^−1^ BW	0.0058	0.00148	0.002	0.0100	Normal
VlymphC	L kg^−1^ BW	0.0036	0.0007	0.0022	0.0050	Normal
VfaC	L kg^−1^ BW	0.1950	0.0400	0.1200	0.2800	Normal
VspdC	L kg^−1^ BW	0.6050	0.1000	0.4500	0.7500	Normal
VrpdC	L kg^−1^ BW	0.0284	0.0020	0.0120	0.0450	Normal
VBldC	L kg^−1^ BW	0.0600	0.0080	0.0410	0.0790	Normal
QCC	L h^−1^ kg^−1^ BW^0.75^	12.0	2	11.1	12.98	Normal
QhepartC	Unit less	0.0690	0.0060	0.0500	0.0900	Normal
QstC	Unit less	0.0110	0.0009	0.0040	0.0160	Normal
QguC	Unit less	0.1700	0.0130	0.1100	0.2300	Normal
QkiC	Unit less	0.2000	0.0015	0.1000	0.2980	Normal
QfaC	Unit less	0.0500	0.0050	0.0300	0.0699	Normal
QspdC	Unit less	0.2870	0.0221	0.2100	0.3600	Normal
QrpdC	Unit less	0.2100	0.0168	0.1600	0.2700	Normal
FracDOSEHep	Unit less	-	-	0.715	0.914	Uniform
FracDOSELymph	Unit less	-	-	0.021	0.079	Uniform
FB_BPA	Unit less	-	-	0.023	0.962	Uniform
FB_BPAG	Unit less	-	-	0.707	0.982	Uniform
FB_BPAS	Unit less	-	-	0.707	0.982	Uniform
MPY	mg/g	34	7	14.6	53.7	Normal
MPYgu	mg/g	3.9	0.8	2.3	5.5	Normal
BELLYPERM	h^−1^	-	-	50	150	Uniform
GIPERM	h^−1^	-	-	5	15	Uniform
BELLYPERMLymph	h^−1^	-	-	0.84	2.5	Uniform
GIPERMLymph	h^−1^	-	-	0.55	1.6	Uniform
KEMAX	h^−1^	-	-	5.1	15	Uniform
KEMIN	h^−1^	-	-	0.0025	0.0075	Uniform
K1_BPA_GUT	h^−1^	-	-	0.55	1.6	Uniform
K1_BPAG_GUT	h^−1^	-	-	0.50	19.3	Uniform
K1_BPAS_GUT	h^−1^	-	-	0.55	1.6	Uniform
K1_BPA_LIVER	h^−1^	-	-	0.55	1.6	Uniform
K1_BPAG_LIVER	h^−1^	-	-	0.005	0.015	Uniform
K1_BPAS_LIVER	h^−1^	-	-	0.005	0.015	Uniform
K1_BPA_Urine	h^−1^	-	-	0.0005	0.0015	Uniform
K1_BPAG_Urine	h^−1^	-	-	0.0005	0.0015	Uniform
K1_BPAS_Urine	h^−1^	-	-	0.00005	0.00015	Uniform
K1_BPA_REMOVED_PLASMA	h^−1^	49.99	-	2.41	97.48	Uniform
K1_BPAG_REMOVED_PLASMA	h^−1^	78.83	-	13.22	146.58	Uniform
K1_BPAS_REMOVED_PLASMA	h^−1^	49.99	-	2.41	97.48	Uniform
K1Lymph	h^−1^	-	-	0.262	0.738	Uniform
Lymphlag	H	-	-	0.28	1.47	Uniform
Vmax_liv_BPA_in_vitro	pmol/min/mg	4494	900	1183	7839	Normal
KM_liv_BPA_in_vitro	mg/L	1.31	0.20	0.154	2.44	Normal
Vmax_liv_BPAG_in_vitro	pmol/min/mg	487	100	290	690	Normal
KM_liv_BPAG_in_vitro	mg/L	18.29	4	10	26	Normal
Vmax_liv_BPAS_in_vitro	pmol/min/mg	73	40	16.50	132.18	Normal
KM_liv_BPAS_in_vitro	mg/L	4.00	0.6	1.63	6.37	Normal
Vmax_gut_BPAG_in_vitro	pmol/min/mg	610	100	262	957	Normal
KM_gut_BPAG_in_vitro	mg/L	22.98	4	9.8	35.10	Normal
Vmax_gut_BPAS_in_vitro	pmol/min/mg	73	10	16.5	132.18	Normal
KM_gut_BPAS_in_vitro	mg/L	4.00	0.6	1.63	6.37	Normal
Pbab	Unit less	-	-	0.36	1.1	Uniform
Plib	Unit less	-	-	0.36	1.1	Uniform
Pkib	Unit less	-	-	1.35	14.67	Uniform
Pfab	Unit less	-	-	1.35	14.67	Uniform
Pgub	Unit less	-	-	1.35	14.67	Uniform
Pstb	Unit less	-	-	1.35	14.67	Uniform
Prpdb	Unit less	-	-	1.4	4.2	Uniform
Pspdb	Unit less	-	-	1.4	4.2	Uniform
PbaG	Unit less	-	-	0.7	2.1	Uniform
PliG	Unit less	-	-	1.59	22.49	Uniform
PkiG	Unit less	-	-	1.59	22.49	Uniform
PfaG	Unit less	-	-	1.2	3.60	Uniform
PguG	Unit less	-	-	1.59	22.49	Uniform
PstG	Unit less	-	-	1.70	5.3	Uniform
PrpdG	Unit less	-	-	2.1	6.4	Uniform
PspdG	Unit less	-	-	1	3	Uniform
PbaS	Unit less	-	-	0.7	2.1	Uniform
PliS	Unit less	-	-	1.59	22.49	Uniform
PkiS	Unit less	-	-	1.59	22.49	Uniform
PfaS	Unit less	-	-	1.3	3.9	Uniform
PguS	Unit less	-	-	1.59	22.49	Uniform
PstS	Unit less	-	-	1.9	5.7	Uniform
PrpdS	Unit less	-	-	2.3	6.8	Uniform
PspdS	Unit less	-	-	1	3.1	Uniform

The *in vitro* metabolic rate constants for the biotransformation of BPA to BPAG and BPA to BPAS were taken from (Mazur et al., 2010) and scaled to whole liver and gut using the microsomal protein yield (Pacifici et al., 1988; Soars et al., 2002) and mass of the liver or gut. Default values for uptake and elimination rate parameters were based on corresponding terms in McNally et al. (2021) and subsequently refined through tuning parameters to better represent the trends in data from the human volunteer study reported in Thayer et al. (2015). The default model assumed 80% of the administered BPA was absorbed through the hepatic route, with a further 5% absorbed through the lymphatic route with the complementary 15% passing through unabsorbed (Thayer et al., 2015).

The baseline model was subsequently refined using an iterative model development process, as outlined in *QIVIVE Workflow* and in Supplementary Material, in order to better represent the trends in BM (blood) data from the human volunteer study reported in Thayer et al. (2015). Technical details on the process of model development and refinement are provided in Supplementary Material.

### 2.2 Experimental Data

#### 2.2.1 Human Biological Monitoring Data

The pharmacokinetics of BPA in humans following a single oral dose of 100 µg/kg body weight (in a vanilla wafer cookie after fasting from the previous midnight) was studied in fourteen, healthy men (*n* = 6) and non-pregnant women (*n* = 8), 26–45 years of age (Thayer et al., 2015). The cohort comprised seven African American and seven non-Hispanic whites with average BMIs of 28.6 and 26.1 for the males and females, respectively. The body weights of individuals 1 to 14 were, 94, 69, 118, 72, 86, 61, 91, 75, 73, 68, 102, 95, 68 and 79 kg. The study was approved by the Research in Human Subjects Committee of the U.S. Food and Drug Administration (Thayer et al., 2015). The biological monitoring data were kindly provided by Dr. Xiaoxia Yang of the Division of Biochemical Toxicology of the U.S. Food and Drug Administration. Plasma concentrations of BPA, BPAG and BPAS were used to calibrate the PBK model.

#### 2.2.2 *In vitro* Data

*In vitro* concentration-response data were obtained from the ToxCast/Tox21 database available from the Bioactivity section of the United States Environmental Protection Agency Chemistry Dashboard. Appropriate datasets were obtained by first filtering to retain only those that were active (positive hit-call) i.e., had an AC_50_ (concentration at which 50% maximum activity was observed) derived from the Hill or Gain-Loss model where both the modelled and observed maximum responses met or exceeded an efficacy cut-off (Filer et al., 2014) and had no warning signs (flags). Two of the selected datasets were generated using HepG2 cells, a human liver cell line and two using HEK293T cells, a human kidney cell line. Datasets with the lowest AC_50_ value were selected. The rationale adopted was analogous to the process followed by regulatory agencies where generally, the lowest NOAEL or BMD value is identified and used for the safety assessment of any given chemical. Four datasets selected for study; none were associated with an Adverse Outcome Pathway (AOP) in the information available from the US EPA Chemistry Dashboard^1^. However, mechanistic and epigenetic effect studies support the conclusion that BPA is an endocrine disruptor and has been associated with an AOP (Carvaillo et al., 2019). It affects several receptor-dependent and independent signalling pathways which perturb hormone homeostasis and gene expression leading to cytogenetic and epigenetic effects. Although, it is emphasised that existence of an AOP is not of importance to the aim of this study. Overall, the primary objective in data selection was the availability of a useful concentration-response profile, not the association of an *in vitro* endpoint consistent with an *in vivo* endpoint.

1) Estrogen receptor activation. (Assay name: ATG_ERE_CIS_up, AC50 = 0.1 µM). No flags.
2) Pregnane X receptor. (Assay name: ATG_PXR_TRANS_up, AC50 = 0.72 µM). No flags.
3) Estrogen receptor activation. (Assay name: OT_ER_ERaERb_0480, AC50 = 0.32 µM). No flags.
4) Estrogen receptor activation. (Assay name: OT_ER_ERaERa_1440, AC50 = 4.31 µM). No flags.

Assays 1-2 were conducted in 24-well plates using human liver HepG2 cells and dimethyl sulfoxide the dilution solvent. Assays 3-4 were conducted using human kidney HEK293T cells in 384-well plates and dimethyl sulfoxide the dilution solvent.

### 2.3 QIVIVE Workflow

The aim of the QIVIVE workflow was to estimate a distribution of ingested concentrations of BPA that was consistent with target tissue concentrations, for comparison against *in vitro* data. *In vivo* hepatic tissue BPA concentrations (CVli) and *in vivo* kidney tissue BPA concentrations (CVki) were selected as suitable target tissue concentrations because HepG2 cells are derived from the human liver and HEK293T cells from the human kidney, respectively and are considered *in vitro* surrogates for the liver and kidney *in vivo*, respectively.

**FIGURE 2:**
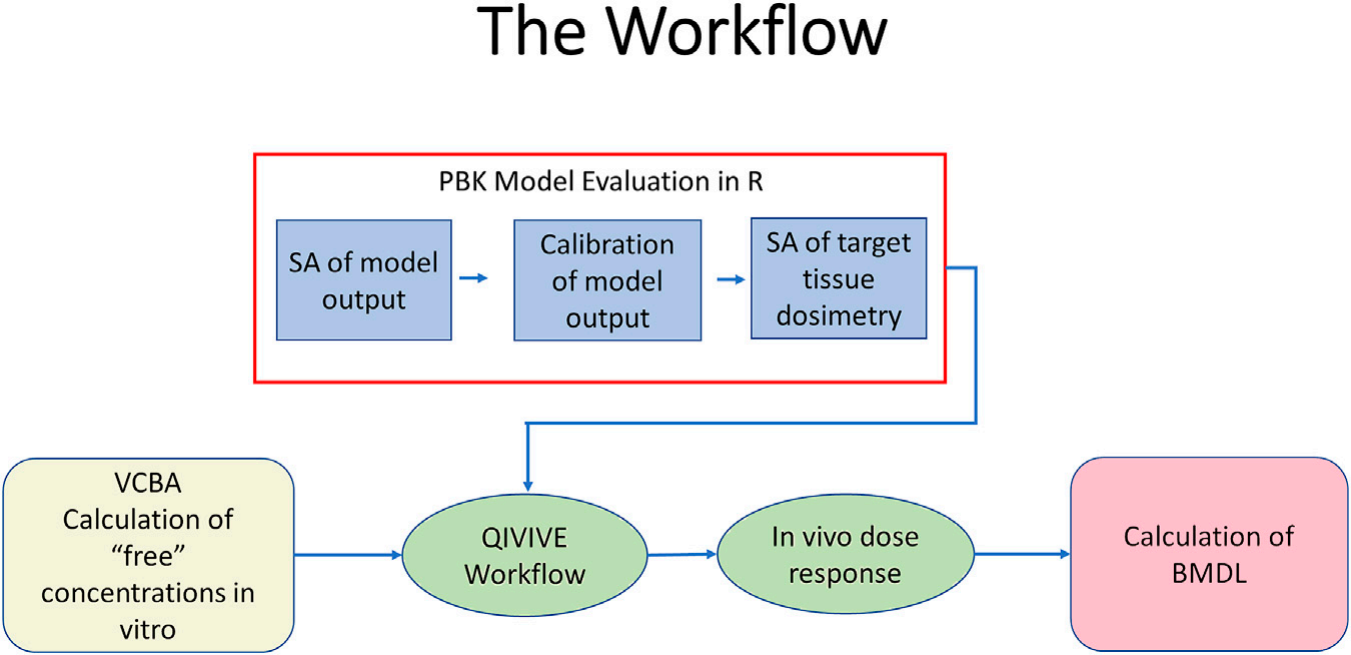
The workflow. PBPK model evaluation was conducted using R (blue fill). This comprised sensitivity analysis of blood BPA following oral uptake, identification of marginal distributions using rejection sampling, calibration of model output using measured blood BPA concentrations followed by sensitivity analysis of *in vivo* target tissue dosimetry of liver (CVli) and kidney (CVki). Free concentrations of BPA *in vitro* were estimated from nominal concentrations using the VCBA (beige fill). Free BPA concentrations and the calibrated PBK model were input in the QIVIVE workflow to estimate *in vivo* dose responses (pale green fill). The latter were used to calculate a BMDL_10_ using PROAST (pink fill).

The key to the approach described below is the recognition that the PBK model is an imperfect approximation to reality. Exact matching of the chosen PBK model response to an *in vitro* concentration suggests a higher degree of belief in the model than is warranted and is thus not desirable. By accepting a discrepancy between the two, within a specified threshold, model uncertainty is thus accommodated, and an error term is created that can be exploited by efficient sampling techniques. The modelling framework was adapted from Loizou et al., 2021, with the addition of the application of the VCBA for the estimation of the *in vitro* free concentration of BPA. Figure 2 shows a schematic of the workflow. The steps were:

1) Probability distributions for tissue volumes, expressed as a fraction of body weight, and tissue blood flows, expressed as a fraction of cardiac output were estimated using a virtual population generated using PopGen (McNally et al., 2014). Specifically, a US population of 10,000 individuals comprised of 2500 Caucasian males; 2500 Caucasian females, 2500 African American males and 2500 African American females, aged 25–45 and with BMI ranging from 19–35 was generated (which captured the characteristics of the human volunteer study population). Probability distributions for tissue volumes and fractional blood flows were based upon statistical analysis of this model output. For other parameters, such as partition coefficients and rate parameters, uniform distributions were ascribed based upon author’s judgement to represent conservative yet credible bounds and refined through the model development process. The probability distributions used in the reported uncertainty and sensitivity analyses and parameter calibrations are given in Supplementary Table SA1 Supplementary Material.
2) Refinement of the parameter ranges through calibration, using the HBM data of Thayer et al. (2015). A statistical model was specified to link predicted concentrations of BPA, BPAG and BPAS in plasma to corresponding HBM data. A Gaussian error model was assumed with calibration achieved using Markov Chain Monte Carlo (MCMC), implemented in GNU MCSim. Technical details on calibration are provided in Supplementary Material. Refined parameter ranges, based upon results from calibration, were used in subsequent steps of the workflow.
3) Elementary effects screening using the Morris Test was conducted to determine the parameters that the model outputs of interest, AUC of hepatic and kidney tissue concentrations of BPA, CVli, and CVki, respectively, were insensitive to. A simulation of 5 h was used, a time period which represented the maximum range of model output variance.
4) The top ranked parameters from the Morris screening were further examined using eFAST, a variance-based sensitivity analysis. The parameters determined by the Morris Test to which the outputs were insensitive were held fixed at default values in this second phase of sensitivity analysis. A reduced parameter set was taken through into subsequent steps in the workflow following the two-phased GSA.
5) Estimation of the bioavailable or “free” *in vitro* concentration of BPA using the Virtual Cell Based Assay (VCBA). The raw data downloaded from the Chemistry dashboard are generally expressed as Log10 µM against the corresponding responses as Log2 fold induction or percentage activity. Calculation of the bioavailable or “free” *in vitro* concentrations of BPA were estimated using the VCBA (Comenges et al., 2017; Paini et al., 2017; Worth et al., 2017; Proença et al., 2019). The algorithm, written in R syntax and available in the Supplementary Material of Proença et al. (2019) was run in RStudio (Version 1.2.1335). The bioavailable fraction was calculated for each *in vitro* dose concentration and used in step 6.
6) Estimation of the distribution of oral dose (PORALDOSE) corresponding to target tissue concentration (the bioavailable fraction of each of the experimental *in vitro* concentrations) whilst accounting for model structure and parameter value uncertainty. This was achieved using a two-step Approximate Bayesian Computation (ABC) approach. In the first phase, 5000 parameter sets were drawn for sensitive parameters from uniform distributions based upon the refined limits resulting from calibration. These were paired with samples drawn for PORALDOSE. The PBK model was run for each of these 5000 parameters sets. The parameter sets that corresponded to predictions of CVli and CVki within ±7.5% of the target *in vitro* concentration were retained and the covariance matrix of the parameters calculated. In the second phase, a more efficient parameter space search was conducted using ABC MCMC. A proposed move was accepted if within ±5% of the target concentration. Four chains were run, each for 50,000 iterations. The above approach was repeated for each of the dose concentrations. Technical details of the approach are given in McNally et al., 2018 and Loizou et al., 2021.

Finally, a PoD, the BMDL_10_ lower bound in the *in vivo* dose response relationship was estimated (see section Calculation of *in vivo* benchmark dose).

### 2.4 Calculation of *in vivo* Benchmark Dose

The dose-response curves were predicted by calculating the AUC of the liver (CVli) or kidney (CVki) tissue concentrations versus fold induction or percentage activity. The first 3 h of the 24-h simulation period captured the maximum AUC for both tissues (data not shown). The mean, 2.5 and 97.5% of the credible interval values were calculated for the most sensitive parameters identified by GSA that determined CVli and CVki variability whilst also estimating the oral exposure concentration (PORALDOSE). PORALDOSE was estimated in µg/kg BW/day for direct comparison with the temporary Tolerable Daily Intake (t_TDI) of 4 μg/kg bw per day for BPA used in the risk assessment conducted by EFSA.

The BMD, BMDL and BMDU were estimated for the 10% BMRs compared to controls from the 90% confidence interval around the mean *in vivo* concentrations and corresponding fold inductions or percentage activities. The 10% BMR was calculated for direct comparison with the value used by EFSA. BMD values were calculated by model averaging from four fitted models for continuous (value for each individual) response data.

### 2.5 Software

The model was coded in the GNU MCSim language (version 6.1.0.)^2^ and run under Windows 10 Pro using RStudio (RStudio Team 2016). Files for running MCSim under windows, tools and instructions for installation are available from Github^3^. All plots were created using R version 4.0.2 and ggplot2 (R Development Core Team 2008; Wickham 2016).

In order to perform probabilistic simulations the model code was further modified to ensure that logical constraints on mass balance and blood flow to the tissues were met by adopting the re-parameterizations described by (Gelman et al., 1996).

The PBK model was evaluated using RVis, an open access PBK modelling platform^4^ which provides an intuitive user-friendly interface with which to interact with MCSim and the R platform^5^. The model equations were solved using MCSim which writes an output file in Tab Separated Values (TSV) format which is then input into the R environment and read by the R packages required for the various analyses. GSA of model outputs (Morris screening test and extended Fourier Amplitude Sensitivity Test (eFAST)) were conducted using the Sensitivity package of R. Reshaping of data and plotting was done using the reshape and ggplot2 packages respectively (Wickham 2007; Pouillot and Delignette-Muller 2010; Soetaert et al., 2010; Pujol et al., 2015). The main effects and total effects (McNally et al., 2011) were computed at each time point and parameter sensitivities were studied over this period using Lowry plots generated as described in McNally et al. (2011).

Benchmark dose values (BMDs) were calculated using PROAST version 69.0 hosted on the EFSA Open Analytics web site^6^.

The PBPK model is provided in Supplementary Material. R scripts for interacting with the model and replicating various stages of the analysis are available from the authors on request.

### 2.6 Hardware

A Dell Precision M4800 with an Intel(R) Core™ i7-4800MQ CPU@2.70 GHz with 32.0 GB RAM running Windows 10 Pro was used for this study.

## 3 Results

### 3.1 Refinement of Exposure Assessment

**FIGURE 3:**
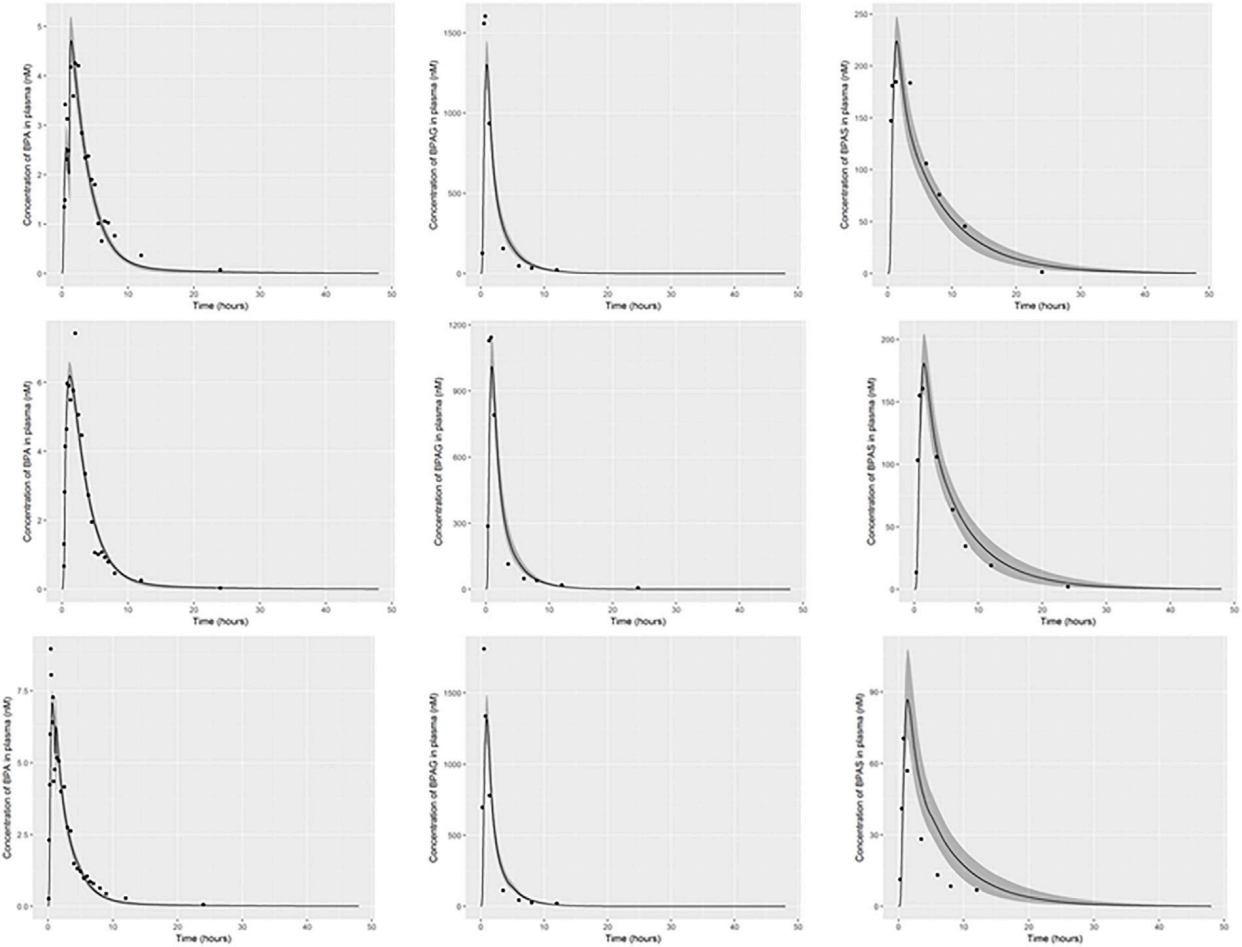
PBK model for BPA was evaluated by simulating the data of Thayer et al. (2015). The panels show serum BPA, BPAG and BPAS from left to right for individual 1, body weight, 94 kg **(upper panel)**, individual 3, body weight, 118 kg **(middle panel)** and individual 5, body weight, 86 kg **(lower panel)**. The solid lines represent the posterior mode-fit and the shaded bands bounding the posterior mode-fit correspond to a numerically derived 95% credible interval.

The parameter ranges for global and individual-specific parameters were refined through calibration of the model using the plasma BPA, BPAG and BPAS data of Thayer et al. (2015). Detailed results from calibration are provided in Supplementary Material; however, a summary of the key results is presented below. The simulations for three individuals only are shown in Figure 3: this subset of results demonstrates the range of behaviour that the calibrated model was able to fit and is used to emphasise key points from the results. Good fits to the plasma data of Thayer et al. (2015) were obtained for all 14 volunteers and the fits to the unique trends of BPA, BPAG and BPAS in plasma from each volunteer are shown in the three panels of Supplementary Figures S2–S12 of Supplementary Material. Summary statistics, that is, prior and refined (posterior) parameter distributions for global (i.e., common to all 14 individuals) and individual-specific parameters, are listed in Table 3 and Table 4.

**TABLE 3:** Global prior and posterior parameter distributions.

Parameter	Median (95% interval)	
	Prior	Posterior
FB_BPA	0.488 (0.023, 0.962)	0.119 (0.006, 0.300)
FB_BPAG	0.845 (0.707, 0.982)	0.920 (0.905, 0.935)
FB_BPAS	0.845 (0.707, 0.982)	0.855 (0.744, 0.927)
KM_liv_BPA_in_vitro	1.31 (0.154, 2.44)	1.543 (0.750, 2.44)
Vmax_liv_BPA_in_vitro	4494 (1183, 7839)	3603 (1726, 6268)
KM_gut_BPAG_in_vitro	22.98 (9.80, 35.91)	27.8 (12.12, 36.07)
Vmax_gut_BPAG_in_vitro	610 (262, 957)	433 (252, 922)
KM_liv_BPAS_in_vitro	4.00 (1.63, 6.37)	4.08 (1.63, 6.38)
Vmax_liv_BPAS_in_vitro	73 (16.50, 132.18)	70.31 (21.77, 123.45)
KM_gut_BPAS_in_vitro	4.00 (1.63, 6.37)	5.16 (2.23, 6.44)
Vmax_gut_BPAS_in_vitro	73 (16.50, 132.18)	40.39 (14.93, 87.92)
KEMAX	12.36 (0.70, 24.37)	1.52 (0.81, 2.85)
GIPERM	12.85 (1.05, 24.46)	15.13 (6.60, 24.33)
BELLYPERM	5.05 (0.36, 9.75)	0.78 (0.11, 1.89)
Pfab	8.02 (1.35, 14.67)	11.58 (1.03, 14.83)
Pgub	8.02 (1.35, 14.67)	3.38 (1.74, 6.62)
Pstb	8.02 (1.35, 14.67)	1.27 (1.01, 3.84)
Pkib	8.02 (1.35, 14.67)	12.00 (5.59, 14.85)
PliG	12.01 (1.59, 22.49)	1.19 (1.00, 1.85)
PliS	12.01 (1.59, 22.49)	3.20 (1.03, 16.44)
PguG	12.01 (1.59, 22.49)	16.75 (4.43, 22.77)
PguS	12.01 (1.59, 22.49)	1.75 (1.01, 8.46)
PkiG	12.01 (1.59, 22.49)	17.94 (7.85, 22.75)
PkiS	12.01 (1.59, 22.49)	11.68 (2.92, 22.31)
K1_BPA_REMOVED_PLASMA	49.99 (2.41, 97.48)	76.38 (28.00, 98.94)
K1_BPAG_REMOVED_PLASMA	79.83 (13.22, 146.58)	118 (55.14, 148.83)
K1_BPAS_REMOVED_PLASMA	49.99 (2.41, 97.48)	46.55 (11.51, 97.03)
K1_BPAG_GUT	9.83 (0.50, 19.53)	15.11 (5.80, 19.74)
K1Lymph	0.497 (0.262, 0.738)	0.592 (0.512, 0.688)

**TABLE 4:** Individual-specific prior and posterior parameter distributions.

Parameter	Prior	Ind1	Ind2	Ind3	Ind4	Ind5	Ind6	Ind7
FracDOSEHep	0.81 (0.715, 0.914)	0.895 (0.816, 0.918)	0.782 (0.704, 0.900)	0.782 (0.706, 0.900)	0.789 (0.705, 0.906)	0.852 (0.729, 0.916)	0.791 (0.708, 0.906)	0.889 (0.793, 0.918)
FracDOSELymph	0.05 (0.021, 0.079)	0.04 (0.03, 0.051)	0.054 (0.042, 0.067)	0.067 (0.052, 0.079)	0.054 (0.042, 0.068)	0.03 (0.021, 0.042)	0.032 (0.234, 0.437)	0.042 (0.032, 0.055)
Lymphlag	0.875 (0.28, 1.47)	1.14 (0.993, 1.28)	0.929 (0.684, 1.28)	0.38 (0.292, 0.657)	0.936 (0.800, 0.989)	1.12 (0.267, 1.32)	0.877 (0.783, 0.979)	0.636 (0.316, 0.728)
MPY	34 (14.6, 53.7)	48.5 (40.3, 53.6)	36.5 (27.5, 48.4)	45.2 (35.1, 53.1)	30.7 (23.3, 40.2)	16.3 (14.1, 21.9)	45.2 (36.2, 53.0)	49.5 (40.5, 53.7)
VrpdC	0.0284 (0.012, 0.045)	0.030 (0.014, 0.043)	0.032 (0.017, 0.043)	0.032 (0.017, 0.044)	0.029 (0.016, 0.042)	0.025 (0.013, 0.041)	0.028 (0.014, 0.043)	0.028 (0.014, 0.042)
VliC	0.035 (0.021, 0.049)	0.029 (0.021, 0.043)	0.039 (0.024, 0.050)	0.042 (0.027, 0.050)	0.031 (0.021, 0.047)	0.035 (0.023, 0.048)	0.031 (0.021, 0.047)	0.025 (0.020, 0.044)
VBldC	0.06 (0.041, 0.079)	0.052 (0.041, 0.070)	0.062 (0.044, 0.080)	0.057 (0.042, 0.075)	0.068 (0.049, 0.085)	0.052 (0.041, 0.071)	0.058 (0.042, 0.077)	0.059 (0.043, 0.077)
QCC	12.00 (11.01, 12.98)	12.00 (11.18, 12.85)	12.00 (11.19, 12.84)	12.25 (11.35, 12.92)	11.85 (11.08, 12.73)	12.42 (11.56, 12.95)	12.05 (11.20, 12.85)	11.82 (11.08, 12.72)
QguC	0.17 (0.11, 0.23)	0.187 (0.142, 0.234)	0.130 (0.095, 0.182)	0.183 (0.134, 0.231)	0.116 (0.092, 0.171)	0.220 (0.172, 0.247)	0.180 (0.134, 0.229)	0.166 (0.120, 0.219)
QstC	0.01 (0.004, 0.016)	0.011 (0.007, 0.015)	0.009 (0.005, 0.014)	0.0100 (0.006, 0.014)	0.008 (0.005, 0.013)	0.013 (0.007, 0.015)	0.009 (0.005, 0.014)	0.100 (0.006, 0.014)
QkiC	0.2 (0.10, 0.298)	0.133 (0.102, 0.210)	0.172 (0.110, 0.259)	0.218 (0.135, 0.282)	0.217 (0.134, 0.289)	0.190 (0.117, 0.271)	0.222 (0.141, 0.289)	0.160 (0.104, 0.259)
**Parameter**	**Prior**	**Ind8**	**Ind9**	**Ind10**	**Ind11**	**Ind12**	**Ind13**	**Ind14**
FracDOSEHep	0.81 (0.715, 0.914)	0.729 (0.701, 0.840)	0.874 (0.749, 0.918)	0.754 (0.703, 0.883)	0.907 (0.859, 0.919)	0.795 (0.706, 0.909)	0.715 (0.700, 0.778)	0.775 (0.704, 0.898)
FracDOSELymph	0.05 (0.021, 0.079)	0.034 (0.024, 0.045)	0.033 (0.022, 0.044)	0.063 (0.049, 0.077)	0.033 (0.021, 0.047)	0.045 (0.030, 0.061)	0.023 (0.020, 0.029)	0.061 (0.046, 0.077)
Lymphlag	0.875 (0.28, 1.47)	0.641 (0.366, 0.984)	0.315 (0.253, 0.480)	0.713 (0.668, 0.741)	0.772 (0.736, 0.814)	0.432 (0.290, 0.580)	0.734 (0.524, 0.995)	0.575 (0.268, 0.719)
MPY	34 (14.6, 53.7)	36.9 (26.8, 48.8)	41.3 (32.2, 51.6)	34.0 (25.9, 43.8)	14.5 (14.0, 16.4)	19.9 (14.7, 28.2)	25.4 (17.7, 37.1)	41.8 (31.2, 52.6)
VrpdC	0.284 (0.012, 0.045)	0.030 (0.017, 0.043)	0.028 (0.015, 0.042)	0.032 (0.016, 0.044)	0.029 (0.015, 0.043)	0.028 (0.014, 0.042)	0.03 (0.016, 0.043)	0.029 (0.015, 0.042)
VliC	0.035 (0.021, 0.049)	0.043 (0.027, 0.050)	0.034 (0.021, 0.049)	0.045 (0.032, 0.049)	0.021 (0.020, 0.023)	0.038 (0.023, 0.049)	0.048 (0.040, 0.050)	0.035 (0.021, 0.049)
VBldC	0.06 (0.041, 0.079)	0.059 (0.042, 0.078)	0.066 (0.047, 0.083)	0.052 (0.041, 0.070)	0.055 (0.041, 0.074)	0.062 (0.045, 0.081)	0.063 (0.045, 0.081)	0.060 (0.044, 0.079)
QCC	12 (11.01, 12.98)	12.17 (11.29, 12.91)	11.66 (11.04, 12.56)	12.31 (11.47, 12.93)	12.33 (11.48, 12.94)	11.94 (11.13, 12.79)	12.05 (11.18, 12.82)	12.04 (11.20, 12.86)
QguC	0.17 (0.11, 0.23)	0.172 (0.118, 0.226)	0.102 (0.090, 0.152)	0.200 (0.150, 0.244)	0.235 (0.199, 0.249)	0.168 (0.117, 0.220)	0.129 (0.092, 0.202)	0.167 (0.115, 0.221)
QstC	0.01 (0.004, 0.016)	0.009 (0.005, 0.014)	0.009 (0.005, 0.014)	0.011 (0.006, 0.015)	0.013 (0.009, 0.015)	0.009 (0.006, 0.014)	0.007 (0.005, 0.013)	0.009 (0.006, 0.014)
QkiC	0.2 (0.10, 0.298)	0.222 (0.146, 0.292)	0.204 (0.115, 0.291)	0.207 (0.133, 0.285)	0.158 (0.110, 0.236)	0.231 (0.152, 0.292)	0.249 (0.170, 0.297)	0.189 (0.115, 0.275)
**Parameter**	**Prior**	**Posterior**
*σ_BPA_*	6.72 (0.32, 22.58)	0.606 (0.551, 0.667)
*σ_BPAG_*	166.68 (8.36, 551.93)	198.85 (166.50, 236.14)
*σ_BPAS_*	16.88 (0.76, 56.71)	23.34 (19.16, 29.02)

Three individual-specific parameters governed variability in the uptake of BPA: the fraction of administered BPA that was available for hepatic uptake; the fraction taken up into the lymphatic system; and a lag term (Lymphlag) which determined the time duration before BPA entered venous blood (at the thoracic duct). Results from calibration (Table 4) showed the majority fraction of BPA, between 70 and 90% of administered BPA, was taken up in the gut and entered hepatic circulation with substantial differences in central estimates and credible intervals between volunteers. This majority fraction was subject to first pass metabolism in gut and liver, with simulations indicating that metabolism was rapid, with little BPA entering systemic circulation from this route. In contrast the fraction entering the lymphatic system was small – between 2 and 7% of administered BPA (with differences in central estimates and credible intervals between volunteers) - however this small fraction was not subject to first pass metabolism. Whilst only a small fraction, consideration of lymphatic uptake was important for successfully fitting 1) the peak of BPA in plasma occurring at later time point compared to metabolites BPAG and BPAS; 2) the double peak of BPA seen in individuals one and five (discussed below); 3) accounting for a slower clearance of BPA from plasma. The influence of the lymphatic component is evident in the plasma BPA data from individuals 1 and 5 (Figure 3) where the double peak fitted for these individuals is a consequence of the fraction entering the lymphatic system. For some participants (Figure 3, participant 3) there was no double peak – this behaviour corresponded to small values of Lymphlag. It is interesting to note that the double peak (characterised by the very rapid decrease and rapid increase at approximately 1.5 h) in individual one occurs during the uptake phase whereas it is in the distribution phase (at approximately 2.5 h) in individual five. This is consistent with a more rapid removal of BPA through metabolism (to BPAG and BPAS) due to the much higher estimated MPY in individual 1 compared to individual 5.

Comparison of the two sets of summary statistics indicates a broad consistency, with modest changes to the medians following calibration, but with a consistent narrowing of credible intervals. This was not true, however, for the following partition coefficients; Pgub, Pstb, PliG, PliS and PguS. For these parameters uniform priors over wide ranges were specified, reflecting the a priori uncertainty in partition coefficients predicted using an in-silico algorithm. Following calibration much narrower marginal posterior distributions were associated with these parameters, reflecting that good fits to HBM data could only be achieved within a much narrower range of parameter space. Similarly, there was a very substantial narrowing of the posteriors for KEMAX, BELLYPERM and GIPERM compared with priors.

### 3.2 GSA

The AUC tissue concentrations for CVli and CVki were studied with sensitivity analysis. The parameter ranges ascribed to model parameters were the refined limits following parameter calibration – for participant specific parameters, the minimum and maximum values were taken over the summary statistics for the 14 individuals. Due to the stochastic nature of the Morris test, parameter rankings were derived by identifying the mode for each parameter over six simulations. The ten parameters to which each of the outputs were most sensitive were selected from the entire set of 74 model parameters for the second phase of sensitivity analysis using the variance-based eFAST method. The outputs were judged to be insensitive to the other parameters; these were held fixed at default values in the second phase of sensitivity analysis. Table 5 lists the parameters following elementary effects screening that had the most significant impact on CVli and CVki tissue concentrations and are ranked according to eFAST.

**TABLE 5:** Sensitivity analysis: parameter ranking.

Serum BPA	Serum BPAG	Serum BPAS	CVli	CVki
KM_liv_BPA_in_vitro	FB_BPAG	FB_BPAS	KM_liv_BPA_in_vitro	KM_liv_BPA_in_vitro
MPY	FracDOSEHep	Vmax_gut_BPAS_in_vitro	MPY	K1_BPA_REMOVED_PLASMA
Vmax_liv_BPA_in_vitro	VspdC	KM_gut_BPAS_in_vitro	KEMAX	Vmax_liv_BPA_in_vitro
VliC	Vmax_gut_BPAS_in_vitro	BELLYPERM	Vmax_liv_BPA_in_vitro	MPY
KEMAX	Pstb	MPY	VliC	Pkib
FB_BPA	VguC	KM_liv_BPA_in_vitro	BELLYPERM	VliC
FracDOSEHep	VliC	VguC	FracDOSEHep	VspdC
VspdC	VBldC	FracDOSEHep	Pgub	FracDOSEHep
BELLYPERM	BELLYPERM	Vmax_liv_BPA_in_vitro	VspdC	Pstb
Pstb	KEMAX	KEMAX	Pstb	QCC

**FIGURE 4:**
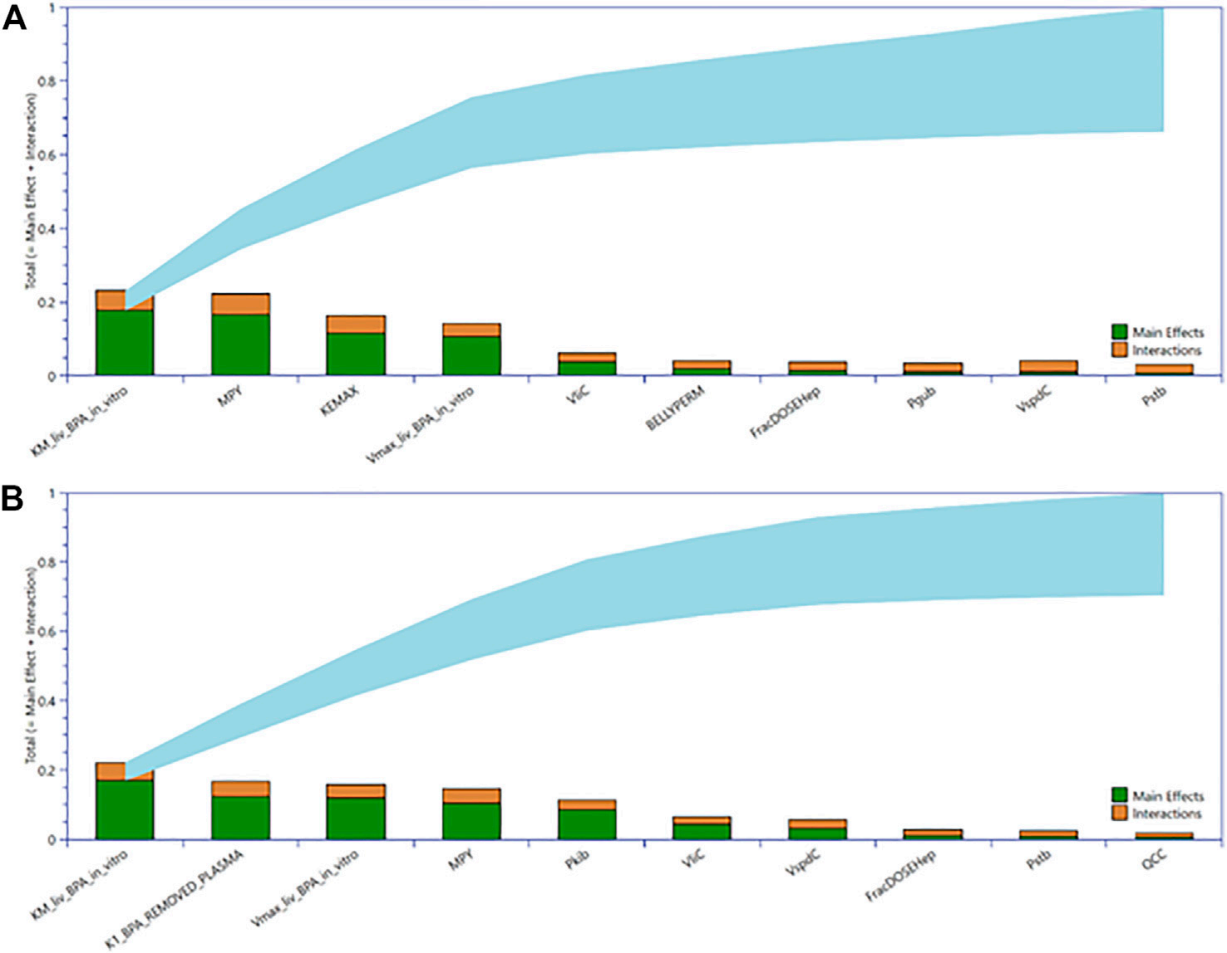
Lowry plots of the most influential parameters governing tissue BPA in liver (CVli) **(A)** and kidney (CVki) **(B)**. The Lowry plot shows the total effect of a parameter S_T_, which is comprised the main effect S_M_ (green bar) and any interactions with other parameters Si (brown bar) given as a proportion of variance. The ribbon (light blue), representing variance due to parameter interactions, is bounded by the cumulative sum of the S_M_ **(lower bound)** and the minimum of the cumulative sum of the S_T_ (upper bound). The S_T_ for top three parameters for CVli **(upper panel)**, and CVki **(lower panel)** accounted for 61 and 55% of variance, respectively.

Figure 4 shows the GSA for tissue concentrations of CVli and CVki as a Lowry plot (McNally et al., 2011). The Lowry plot shows the total effect of a parameter S_T_, which is comprised of the main effect S_M_ (green bar) and any interactions with other parameters S_i_ (brown bar) given as a proportion of variance (range 0 – 1 on *y* axis) (McNally, et al., 2011). The ribbon (light blue), representing variance due to parameter interactions, is bounded by the cumulative sum of the S_M_ (lower bound) and the minimum of the cumulative sum of the S_T_ (upper bound). The most significant parameters that contributed to variance throughout the 24-h simulation period range from left to right. The 10 most significant parameters dominated from 0 to 5 h and remained in that order throughout that period whereas some swapping of order was observed for the other less important parameters. Furthermore, the S_T_ for the top three parameters for CVli (upper panel), and CVki (lower panel) accounted for 61 and 55% of variance, respectively. Parameter sensitivities from 0 to 5 h, the period of maximum variance and the period for which the AUC was calculated for use in QIVIVE, remained at similar proportions of variance throughout the simulation period of 24 h. To reduce simulation time, the top eight parameters for CVli and CVki listed in Table 5 were used in ABC MCMC.

Similar observations were made for plasma BPA, BPAG and BPAS where the top ten parameters shown in Table 5 accounted for 54, 82 and 66% variance, respectively.

### 3.3 *In vitro* Bioavailable Concentrations

The physicochemical parameters required to run the VCBA for BPA are listed in Table 6. The raw data from the Chemistry dashboard alongside the transformed nominal and calculated free *in vitro* concentrations and the ratios of free to nominal concentrations for all assays using the VCBA are presented in Table 7 and Table 8. The bioavailable concentrations of BPA were predicted to be around 48–49% of the nominal concentration (in the presence of 5% serum) for all the selected assays.

**TABLE 6:** Physicochemical parameters to run Virtual Cell Based Assay for Bisphenol A.

Parameters	Value
Molecular weight (MW; g/mol)	228.291
Molecular diffusion volume	220.14
Molar volume (MV; cm^3^/mol)	200
Henry law constant (HLC; Pa×m^3^/mol)	9.28 × 10^–7^
Degradation rates in water (s^−1^)	2.14 × 10^–7^
Degradation rates in air (s^−1^)	6.42 × 10^–5^
Log Kow (unitless)	3.32

**TABLE 7:** Concentration-response data from Chemistry Dashboard^a^

ATG_PXR_TRANS_up						
Nominal *in vitro* concentration (µM/L)^b^	LOG_10_ *in vitro* Concentration (µM/L)^b^	Fold Induction (log2)^b^	Nominal *in vitro* concentration (mg/L)^c^	Free concentration (mg/L)^c^	Fold Induction (Natural Scale)^d^	Ratio
0.01	−2.000	−0.050	2.28E-03	1.14E-03	0.966	0.499
0.03	−1.523	0.257	6.85E-03	3.42E-03	1.190	0.499
0.09	−1.046	0.056	2.05E-02	1.03E-02	1.040	0.501
0.3	−0.523	0.277	6.85E-02	3.42E-02	1.210	0.499
0.8	−0.097	1.689	1.83E-01	9.12E-02	3.220	0.499
2	0.301	2.711	4.57E-01	2.28E-01	6.550	0.499
7	0.845	2.740	1.60E+00	7.98E-01	6.680	0.499
20	1.301	2.547	4.57E+00	2.28E+00	5.840	0.499
70	1.845	2.340	1.60E+01	7.97E+00	5.060	0.499
ATG_ERE_CIS_up						
0.01	−2.000	−0.122	2.28E-03	1.14E-03	0.919	0.499
0.03	−1.523	0.239	6.85E-03	3.42E-03	1.180	0.499
0.09	−1.046	1.144	2.05E-02	1.03E-02	2.210	0.499
0.3	−0.523	2.046	6.85E-02	3.42E-02	4.129	0.499
0.8	−0.097	2.442	1.83E-01	9.12E-02	5.432	0.499
2	0.301	2.717	4.57E-01	2.28E-01	6.576	0.499
7	0.845	2.368	1.60E+00	7.98E-01	5.163	0.499
20	1.301	2.053	4.57E+00	2.28E+00	4.149	0.499
70	1.845	2.427	1.60E+01	7.97E+00	5.378	0.499

a https://comptox.epa.gov/dashboard/dsstoxdb/results?search=DTXSID7020182#invitrodb-bioassays-toxcast-tox21

b Raw data in form available from Chemistry Dashboard.

c Transformed using the VCBA.

d Natural scale required for BMD calculation.

**TABLE 8:** Concentration-response data from Chemistry Dashboard and estimates of free concentrations^a^

OT_ER_ERaERb_0480						
Nominal *in vitro* concentration (µM/L)^b^	LOG_10_ *in vitro* Concentration (µM/L)^b^	Percentage Activity^b^	Nominal *in vitro* concentration (mg/L)^c^	Free concentration (mg/L)^c^	Percentage Activity^d^	Ratio
0.003	−2.523	−6.989	0.001	0.000342	1.00	1.499
0.003	−2.523	−4.593	0.001	0.000342	1.00	0.499
0.003	−2.523	−4.548	0.001	0.000342	1.00	0.499
0.01	−2.000	2.330	0.002	0.00114	1.00	0.500
0.01	−2.000	−0.599	0.002	0.00114	1.00	0.500
0.01	−2.000	3.617	0.002	0.00114	1.00	0.500
0.03	−1.523	−5.303	0.007	0.00342	1.00	0.499
0.03	−1.523	−5.480	0.007	0.00342	1.00	0.499
0.03	−1.523	−0.333	0.007	0.00342	1.00	0.499
0.1	−1.000	−0.954	0.023	0.0114	1.00	0.482
0.1	−1.000	−1.176	0.023	0.0114	1.00	0.482
0.1	−1.000	2.019	0.023	0.0114	3.019	0.482
0.3	−0.523	26.914	0.069	0.0342	27.914	0.499
0.3	−0.523	30.286	0.069	0.0342	31.286	0.499
0.3	−0.523	24.074	0.069	0.0342	25.074	0.499
1	0.000	72.620	0.228	0.1140	73.620	0.482
1	0.000	78.744	0.228	0.1140	79.744	0.482
1	0.000	60.373	0.228	0.1140	61.373	0.482

OT_ER_ERaERa_1440					
Nominal *in vitro* concentration (µM/L)	LOG_10_ *in vitro* Concentration (µM/L)	Percentage Activity	Nominal *in vitro* concentration (mg/L)	Free concentration (mg/L)	Ratio
0.3	−0.523	3.130	0.069	0.034	0.499
0.3	−0.523	3.790	0.069	0.034	0.499
0.3	−0.523	5.390	0.069	0.034	0.499
1	0.000	17.480	0.228	0.110	0.482
1	0.000	15.450	0.228	0.110	0.482
1	0.000	15.040	0.228	0.110	0.482
3	0.477	72.430	0.685	0.330	0.482
3	0.477	73.940	0.685	0.330	0.482
3	0.477	70.810	0.685	0.330	0.482
10	1.000	138.290	2.280	1.100	0.482
10	1.000	152.640	2.280	1.100	0.482
10	1.000	150.180	2.280	1.100	0.482
30	1.477	186.120	6.850	3.300	0.482
30	1.477	168.100	6.850	3.300	0.482
30	1.477	187.980	6.850	3.300	0.482
100	2.000	196.670	22.800	11.010	0.482
100	2.000	191.740	22.800	11.010	0.482
100	2.000	177.490	22.800	11.010	0.482

a https://comptox.epa.gov/dashboard/dsstoxdb/results?search=DTXSID7020182#invitrodb-bioassays-toxcast-tox21

b Raw data in form available from Chemistry Dashboard.

c Transformed using the VCBA.

d Removal of negative values and two positive values at 0.01 µM/L (considered measurement anomalies) was required for BMD calculation. A constant of 1 was added to each percentage activity to replace zero values at low concentrations.

### 3.4 Quantitative *in vitro in vivo* Extrapolation

The percentage activity measure of response for assay OT_ER_ERaERb_0480 included 9 negative and two positive values at the lowest *in vitro* concentrations (Table 8). This occurs when activities are normalised using controls. This is a range of minimal activity associated with controls where the mean was assigned to zero. In most ToxCast assays a threshold point based on all controls is assigned and activity below the threshold is not considered to indicate anything about the test chemical. Therefore, values in this range were assigned zero on the assumption that the interpretation of this dataset does not change (Filer et al., 2014) (Dr. John Wambaugh, personal communication). A constant of one was then added to the entire range of percentage activities which was required for BMD analysis which assumes a lognormal distribution.

**FIGURE 5:**
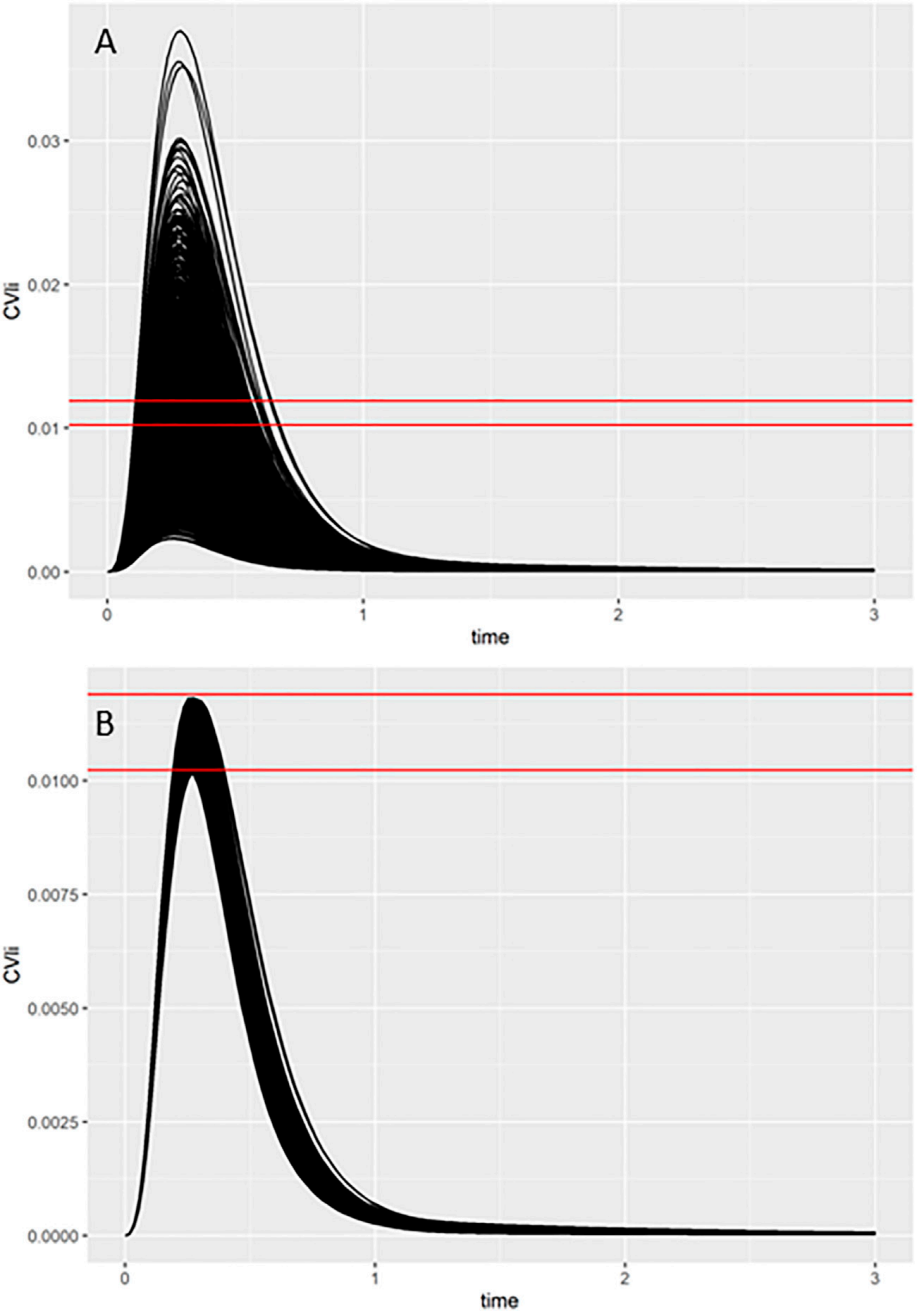
Comparisons of concentration-time response profiles simulated in the rejection phase were run for each concentration. A typical example shows the target concentration of 0.011 mg/L bounded by two red lines representing the 7.5% range above and below the target concentration. **(A)** 5000 concentration-response profiles **(upper panel)**, and **(B)** retained samples within a relative error of 7.5% **(lower panel)**.

As described in the *Materials and Methods* and in previous reports (McNally et al., 2018; Loizou et al., 2021), a two-stage approach was used to sample PORALDOSE that was consistent with the free *in vitro* experimental data and a modest degree of model uncertainty. The latter was accounted for through accepting simulations within 5% of the target *in vivo* concentration. The first phase involves rejection sampling and is illustrated in Figure 5. Panel A of Figure 5 typically illustrates concentration-response profiles from 5,000 simulations, whereas panel B shows the concentration-response profiles from the retained simulations that were within 7.5% of the target concentration (0.011 mg/L, in this example). Plots like these were obtained for each *in vitro* concentration for each of the four assays listed in Tables 7, 8. In the second phase an ABC MCMC algorithm was applied for more efficient sampling of parameter space, in this case within a tighter threshold of 5%, consistent with a given *in vitro* target concentration. This two-stage process was repeated for each *in vitro* concentration with acceptance rates between 7.5 and 35%. Subsequent analysis was based upon results from the retained samples and pooled over the four chains run for QIVIVE for each *in vitro* concentration.

The posterior means and 95% credible interval for the *in vivo* dose-response relationships for PORALDOSE (µg/kg BW/Day) are provided in Table 9 for each target concentration in the assay.

**TABLE 9:** Posterior means and 97.5% credible ranges for PORALDOSE.

PORALDOSE (µg/kg BW/Day)			
Free concentration (mg/L)	ATG_ERE_CIS_up^a^	ATG_PXR_TRANS_up^b^	
0.0011	0.073(0.044, 0.101)	0.071(0.045, 0.099)	
0.0034	0.207(0.113, 0.296)	0.206(0.112, 0.295)	
0.0103	0.617(0.340, 0.892)	0.636(0.325, 0.892)	
0.0342	1.983(1.164, 2.796)	2.000(1.136, 2.780)	
0.0912	4.932(3.072, 6.767)	5.067(3.191, 6.892)	
0.2280	11.054(5.691, 16.713)	11.668(6.316, 17.315)	
0.7979	28.415(18.999, 37.114)	27.969(18.873, 37.635)	
2.2794	47.675(29.234, 65.224)	46.346(30.225, 65.934)	
7.9735	63.492(39.384, 91.921)	71.190(42.385, 99.022)	
**Free concentration (mg/L)**	**OT_ER_ERaERb_0480** ^**a**^	**Free concentration (mg/L)**	**OT_ER_ERaERa_1440** ^**a**^
0.000342	0.064(0.036, 0.088)	0.0342	5.506(2.647, 8.286)
0.000342	0.064(0.037, 0.091)	0.0342	5.984(3.199, 8.671)
0.000342	0.066(0.041, 0.089)	0.0342	5.776(2.937, 8.357)
0.00114	0.218(0.123, 0.302)	0.1140	15.756(8.346, 23.086)
0.00114	0.210(0.108, 0.293)	0.1140	16.479(9.024, 23.656)
0.00114	0.222(0.115, 0.297)	0.1140	15.792(9.130, 23.596)
0.00342	0.641(0.370, 0.918)	0.3300	35.363(15.713, 50.038)
0.00342	0.606(0.317, 0.848)	0.3300	35.934(18.461, 54.609)
0.00342	0.636(0.322, 0.881)	0.3300	37.669(17.623, 53.009)
0.0114	1.944(1.028, 2.660)	1.1000	55.016(36.066, 80.310)
0.0114	1.946(1.077, 2.770)	1.1000	61.304(35.836, 84.071)
0.0114	1.900(1.018, 2.686)	1.1000	60.767(35.497, 87.693)
0.0342	5.818(3.056, 8.414)	3.3000	71.272(41.800, 102.735)
0.0342	5.908(3.096, 8.405)	3.3000	83.133(41.956, 117.018)
0.0342	5.539(2.809, 8.167)	3.3000	73.174(39.195, 109.996)
0.1140	16.179(8.797, 23.327)	11.000	89.123(53.953, 137.919)
0.1140	15.493(7.909, 22.599)	11.000	88.440(53.447, 132.985)
0.1140	15.608(8.027, 22.916)	11.000	101.950(54.850, 142.909)

a Estrogen receptor activation.

b Pregnane X receptor binding.

The summary statistics for PORALDOSE were extrapolated from hepatic tissue concentrations (CVli) estimated from the *in vitro* concentration-response datasets; ATG_ERE_CIS_up (estrogen receptor activation), ATG_PXR_TRANS_up (pregnane X receptor binding), and from kidney tissue concentrations (CKli) estimated from OT_ER_ERaERb_0480 (estrogen receptor activation) and OT_ER_ERaERa_1440 (estrogen receptor activation).

### 3.5 Benchmark Dose Analysis

**FIGURE 6:**
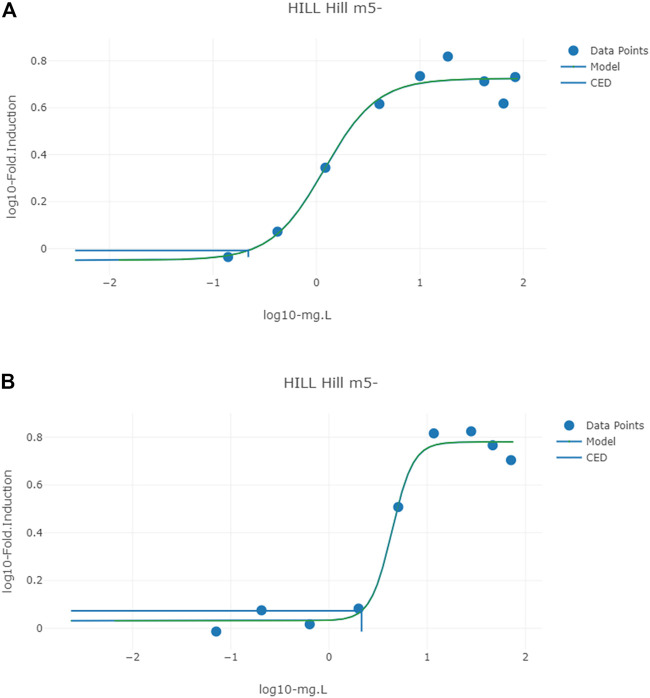
Typical predicted *in vivo* dose-response curves for PORALDOSE extrapolated from hepatic tissue concentration (CVli) for the *in vitro* datasets ATG_ERE_CIS_up (estrogen receptor activation) **(A)** and pregnane X receptor binding ATG_PXR_TRANS_up **(B)**. The curves for the means only are shown. Benchmark dose values were calculated from such curves for lower and upper bounds (2.5 and 97.5%) of the credible intervals (Table 10).

The mean *in vivo* dose responses shown in Table 9 were used to calculate a BMDL_10_ (lower limit of the 95% confidence interval on the BMR equivalent to a 10% effect size) for each *in vitro* assay (Figure 6). The mean, 2.5 and 97.5% percentile BMDL_10_ values calculated for PORALDOSE were compared with the HED adjusted BMDL_10_ and t_TDI values calculated by EFSA for relative kidney weight change in the mouse (Table 10). The mean BMDL_10_ values for PORALDOSE showed 48-fold variability, ranging from 2.7 to 1300 µg/kg BW/day (Table 10). However, these BMDL_10_ values may justifiably be adjusted by dividing with a chemical specific adjustment factor (CSAF) for inter-individual variability in pharmacokinetics (Bhat et al., 2017). Such a CSAF can be calculated by dividing the posterior 97.5% quantile by the median for a parameter that is responsible for the greatest variability in each output. In this case, the CSAF calculated for the fraction of BPA bound to plasma proteins, FB_BPA, is 2.52 (Table 3). The BMDL_10_ of 516 µg/kg BW/day for the ATG_PXR_TRANS_up was similar to the HED adjusted BMDL_10_ derived by EFSA. The BMDL_10_ of 329 µg/kg BW/day is about 54% the HED adjusted BMDL_10_ derived by EFSA. The application of an additional UF to an *in vivo* BMDL_10_ calculated using an isolated human cell line to derive a t_TDI is a matter for discussion but could reduce these values further.

**TABLE 10:** BMDL_10_ mode and 95% credible intervals for daily oral dose.

Oral dose (µg/kg BW/day)				
Assay	1	2	3	4
Nominal Concentration				
Mean	5.6	2690	2700	3730
Lower	2.85	1400	1400	2960
Upper	11.1	3800	4000	4240
Mean/CSAF	2.2	1067	1071	1480
FreeConcentration				
Mean	2.7	1300	960	693
Lower	0.3	790	530	415
Upper	5.7	1800	1300	828
Mean/CSAF	1.1	516	381	329
Lower/CSAF	0.27	718	210	377
Upper/CSAF	5.2	1636	516	753
Ratios^a^				
Mean/CSAF		469	346	299
Lower/CSAF		2659	778	1396
Upper/CSAF		314	99	145
EFSA	HED^b^ BMDL_10_ (Relative mouse kidney weight)	t_TDI		
Mean	609	4^c^		
AC50 (µM)	0.1	0.72	0.32	4.31
AC50 ratios		7.2	3.2	43.1

Assays; 1 = ATG_ERE_CIS_up (Estrogen receptor activation,) 2 = ATG_PXR_TRANS_up (Pregnane X receptor binding), 3 = OT_ER_ERaERb_0480 (Estrogen receptor activation), 4 = OT_ER_ERaERa_1440 (Estrogen receptor activation).

a Ratio = higher/lowest.

b HED = human equivalent dose.

c HED divided by an uncertainty factor (UF) of 150.

The BMDL_10_ of 1.07 µg/kg BW/day with application of the CSAF or 2.7 µg/kg BW/day, without application of the CSAF, derived from the ATG_ERE_CIS_up assay was similar to the EFSA t_TDI of 4 µg/kg BW/day.

The mean *in vivo* BMDL_10_ values for BPA calculated for the four ToxCast assays is considerable, ranging from 2.7 to 1300 µg/kg BW/day or 1.1–516 µg/kg BW/day with application of a CSAF of 2.52. The ratios of the higher to the lowest values of the latter (i.e., 516/1.1, 381/1.1 and 329/1.1) ranged from 299 to 469-fold which were far higher than the differences in the *in vitro* AC50 values which ranged from 3.2 to 43.1-fold (Table 10).

Also, the ratios of BMDL_10_ calculated from free versus nominal concentrations were 0.48 for three of the four assays. Therefore, the ratio of free to nominal BMDL_10_ is consistent with the ratio of *in vitro* free to nominal concentrations in three from four assays.

**FIGURE 7:**
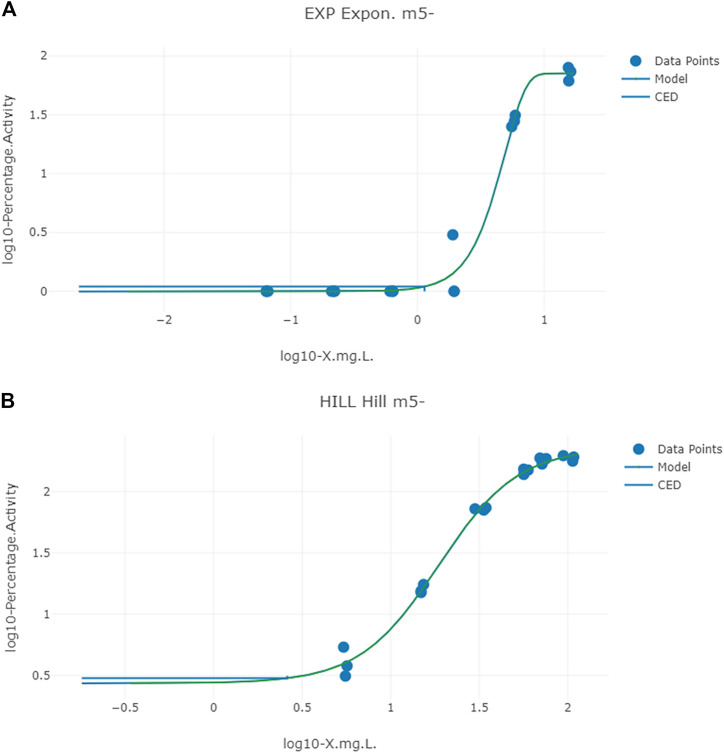
Typical predicted *in vivo* dose-response curves for PORALDOSE extrapolated from kidney tissue concentration (CVki) for the *in vitro* datasets OT_ER_ERaERb_0480 (estrogen receptor activation) **(A)** and OT_ER_ERaERa_1440 (estrogen receptor activation) **(B)**. The curves for the means only are shown. Benchmark dose values were calculated from such curves for lower and upper bounds (2.5 and 97.5%) of the credible intervals (Table 10).

Typical predicted *in vivo* dose-response curves for PORALDOSE extrapolated from hepatic tissue concentration (CVli) for the *in vitro* datasets ATG_ERE_CIS_up (estrogen receptor activation), pregnane X receptor binding ATG_PXR_TRANS_up, and kidney tissue concentration (CVki) for the *in vitro* datasets OT_ER_ERaERb_0480 (estrogen receptor activation) and OT_ER_ERaERa_1440 (estrogen receptor activation) are shown in Figures 6 and 7.

## 4 Discussion

As we noted in the introduction, numerous groups have used PBK modelling to translate *in vitro* concentration-response data into *in vivo* concentration-response data, and thus to estimate a PoD. A “dose matching” approach was utilised by these groups, whereby model parameters, except dose, are held at fixed values and external dose is varied in order to minimise the discrepancy between model prediction and target concentration (corresponding to *in vitro* dose). Such an approach considers neither parameter value uncertainty, nor model uncertainty. Whilst an optimisation procedure may well have been deployed in order to determine the baseline model parameters (that are subsequently held fixed), the optimal parameter set will depend upon the assumptions in the optimisation procedure applied, and furthermore a subset of parameter space typically provides a similar quality of fit to calibration data. Without a consideration of parameter value uncertainty, the sensitivity of the calculated PoD to the choice of baseline parameters is not assessed. Model uncertainty is also important, since the PBK model is an imperfect surrogate for the human (or animal) and results could furthermore depend upon the fidelity of the model. When a single value such as AUC or a maximum concentration is being extracted from PBK model output for direct use in hazard assessment, it implies a greater belief in the adequacy of the surrogate model than is justified.

Understanding and quantifying the level of uncertainty in each step of a chemical safety assessment with NAMs is important for the development of confidence in this approach (Berggren et al., 2017). In our previous studies, we demonstrated a computationally efficient workflow for model evaluation (utilising techniques for uncertainty and sensitivity analysis as a critical part of model development and evaluation), and accounted for parameter value and model error when conducting QIVIVE, thus accounting for two significant sources of uncertainty.

In this study a further significant refinement of the approach has been made as we investigated another area of potentially significant uncertainty - the effect of calculating *in vivo* BMDL_10_ from free versus nominal concentrations. Preliminary estimations using the VCBA of the free to nominal *in vitro* concentrations for perfluorooctanoic acid and chlorpyrifos predict ratios of 0.037 and 9.3%, respectively. These are both significantly lower than the 48% for BPA. These ratios suggest that significantly erroneous HBGV could be derived if based on nominal *in vitro* concentrations.

The mean *in vivo* BMDL_10_ values calculated from free *in vitro* concentrations for assays 1 to 3 were approximately 48% of the mean *in vivo* BMDL_10_ calculated from nominal *in vitro* concentrations. The free to nominal BMDL_10_ ratio for assay 4 was approximately 19%. That is, the ratio of free to nominal *in vitro* concentrations were consistent with the calculated BMDL_10_ ratios for three from four assays. It may be inferred that the relationship between *in vivo* tissue concentrations and nominal *in vitro* concentrations was linear in three from four assays but not for the fourth. Without further investigation the reason for this difference is unclear and would require a detailed analysis of the curve-fitting mathematical models used to analyse the data.

The differences in mean *in vivo* BMDL_10_ values for the various assays exceeded the concomitant differences in in vitro AC50 values. The increased variability of *in vivo* BMDL_10_ values is likely due to intra-individual variations in organ and tissue masses, regional blood flow rates, and metabolism which is not considered in the *in vitro* assays. The differences are even higher for the lower *in vivo* BMDL_10_ values ranging from 1396 to 2659-fold which may be due to increased biological variability at the extreme tails of the lower confidence intervals. These observations provide further support for the use of probabilistic rather than deterministic models in QIVIVE.

The European Food Safety Authority (EFSA) panel on food contact materials, enzymes, flavourings and processing aids (CEF) set a temporary Tolerable Daily Intake (t_TDI) of 4 μg/kg bw per day for BPA using a weight of evidence approach (EFSA Panel on Food Contact Materials and Aids 2015). The latter involved ascribing a “likelihood” level for the occurrence of potential critical toxicological endpoint(s) for the derivation of a HBGV (EFSA Scientific Committee et al., 2017a). The hazard characterisation of BPA was based on increases in liver and kidney weights in rats and mice as likely critical endpoints, with the likelihood of immunotoxic, cardiovascular and metabolic effects classified as “as likely as not” and genotoxicity and carcinogenicity as “unlikely”. A benchmark dose 10% lower confidence limit (BMDL_10_) of 8 960 μg/kg bw per day was calculated for changes in the mean relative kidney weight in a two-generation toxicity study in mice. With the availability of human and animal toxicokinetic data and the use of PBK modelling this value was converted to a human equivalent dose (HED) of 609 μg/kg bw per day. Briefly, the BMDL_10_ of 8 960 was multiplied by a Human Equivalent Dose Factor (HEDF) of 0.068 calculated from the area-under-the curve (AUC) of plasma unconjugated BPA concentrations in mice and humans, (HEDF = AUC_Mice_/AUC_Human_) following a standard oral dose of 100 μg/kg bw per day. Finally, the HED was divided by an overall UF of 150 to obtain the t_TDI of 4 μg/kg bw per day.

The use of hormone binding assays such as, human liver pregnane X and kidney estrogen receptor activation *in vitro* in the risk assessment of BPA may be justified by observations from an occupational exposure study conducted on 3394 subjects (40 years or older) of a Chinese population. In this study high urinary BPA concentrations were correlated with an increased concentration of free triiodothyronine and a decreased concentration of thyroid stimulating hormone (Wang et al., 2013). The authors concluded that there was an association between BPA exposure and altered thyroid hormones. However, this study was not considered by the CEF because it did not meet its geographical origin criteria (EFSA Panel on Food Contact Materials and Aids 2015).

In addition, mechanistic and epigenetic effect studies support the conclusion that BPA is an endocrine disruptor which affects several receptor-dependent and independent signalling pathways which perturb hormone homeostasis and gene expression leading to cytogenetic and epigenetic effects (Rochester 2013; EFSA Panel on Food Contact Materials and Aids 2015; Mesnage et al., 2017). *In vitro* studies have shown that BPA affects not only the estrogenic system but also the functions of androgens, prolactin, insulin and thyroid hormones (Wetherill et al., 2007; Fenichel et al., 2013). Therefore, it is not surprising that BPA has been screened under the Endocrine Disruptor Screening Program for the 21st Century^7^ and the Toxicity Forecaster (ToxCast) Program^8^. A range of *in vitro* concentration-response assays for thousands of chemicals are available from the ToxCast/Tox21 database on the United States Environmental Protection Agency Chemistry Dashboard^9^.

ToxCast was created as a screening program which is reflected in the assay design. It was not intended for the identification of molecular initiating events (MIEs) in the development of Adverse Outcome Pathways (AOPs). The purpose of the program was to maximise throughput, minimise false negatives and facilitate data processing for computational exercises and modelling to identify patterns (Ryan 2017).

The mean CSAF adjusted BMDL_10_ for liver pregnane X receptor (ATG_PXR_TRANS_up) and kidney estrogen receptor activation (OT_ER_ERaERb_0480 and OT_ER_ERaERa_1440) at approximately at 85, 62 and 54% of the HED BMDL_10_ derived by EFSA were similar in magnitude. EFSA applied an UF of 150 for inter- and intra-species differences and uncertainty in mammary gland, reproductive, neurobehavioural, immune and metabolic system effects to establish a t_TDI of 4 µg/kg bw per day. Application of an UF for *in vitro* to *in vivo* extrapolation, which is yet to be determined, in addition to the proposed CSAF used in this study could reduce the BMDL_10_ to values similar to the EFSA t_TDI for three of the four assays.

Finally, the ABC algorithm in combination with the VCBA represents an in silico infrastructure that may be successfully and effectively used to translate *in vitro* concentration-response data for environmental pollutants from the Tox21 and ToxCast high-throughput *in vitro* screening programs and any *in vitro* concertation-response data in general. This algorithm is consistent with the workflow proposed by Bell et al. (2018) and could be an effective tool in a NAMs based chemical risk assessment strategy.

## Data Availability

The original contributions presented in the study are included in the article/Supplementary Material, further inquiries can be directed to the corresponding author.

## Footnotes

1
https://comptox.epa.gov/dashboard/dsstoxdb/results?search=DTXSID7020182#invitrodb-bioassays-toxcast-tox21 (as on 27/09/2021)

2
https://www.gnu.org/software/mcsim/

3
https://github.com/GMPtk/MCSimViaRtools

4
http://cefic-lri.org/toolbox/r-vis-open-access-pbpk-modelling-platform/

5
https://github.com/GMPtk/RVis/releases

6
https://efsa.openanalytics.eu/

7
https://www.epa.gov/endocrine-disruption/endocrine-disruptor-screening-program-edsp-overview

8
https://www.epa.gov/chemical-research/toxicity-forecasting

9
https://comptox.epa.gov/dashboard
