# Supplementary material for: Derivation of a Human In Vivo Benchmark Dose for Bisphenol A from ToxCast In Vitro Concentration Response Data Using a Computational Workflow for Probabilistic Quantitative In Vitro to In Vivo Extrapolation
Source: Front Pharmacol. 2022 Feb 11;12:754408. doi: 10.3389/fphar.2021.754408 (PMC8874249; doi:10.3389/fphar.2021.754408)
Supplement: Supplementary file 1 [file DataSheet1.pdf]

Supplementary material to *Derivation of a Human In Vivo Benchmark Dose for Bisphenol A from ToxCast In Vitro Concentration Response Data Using a Computational Workflow for Probabilistic Quantitative In Vitro to In Vivo Extrapolation*

George Loizou[1], Kevin McNally[1], Alicia Paini[2] and Alex Hogg[1]

Authors' affiliation

[1] Health and Safety Executive, Harpur Hill, Buxton, UK

[2] European Commission Joint Research Centre, Ispra, Italy

PBPK MODEL

A human PBPK model was developed to study the fate of BPA following single oral doses. The initial model was based upon the PBPK model for the plasticizer DPHP described in McNally et al. (2012) with adaptions made as necessary. The final model described entry of BPA through ingestion with absorption of BPA from the gut and gastro-intestinal (GI) tract and a simple model of the lymphatic system describing uptake of BPA via the lacteals in the intestine and entering venous blood after bypassing the liver. The dose that entered the lymphatic system was coded as a fraction of the administered dose; a fraction of administered dose was coded as entering the liver via the portal vein; a fraction of the administered dose of BPA passed through the intestine without being absorbed and was coded as absorbed dose minus the lymphatic and hepatic dose components. The model described the metabolism of BPA to BPAG and BPAS in both liver and gut. Sub-models were included to describe the kinetics of BPAG and BPAS, with the models for BPA and the two metabolites connected via gut and liver. Binding of BPA, BPAG and BPAS was coded from arterial blood, with the consequence that only the unbound fraction in blood was available for distribution to organs and tissues, metabolism, and elimination.

The model structures for BPA and the two metabolites differed only in the coding of uptake required for BPA (including the simplified description of a lymphatic compartment). BPA and metabolite models had a stomach and GI tract draining into the liver. Adipose, blood, kidney, and slowly and rapidly perfused compartments were included. Elimination of BPA, BPAG and BPAS was coded through the kidney compartment, with first order elimination rates, proportional to kidney tissue concentration, coded in each case. Both BPA and metabolite models included the transport process of enterohepatic recirculation. Uptake of BPA and metabolites from the liver into bile was modelled as a first order uptake process with a delay of four hours (to represent transport in bile) before BPA (and metabolites) appeared in the gut and were available for reabsorption. First order elimination rates for each substance were coded to account for fractions of recirculated BPA and metabolites that were eliminated in faeces rather than reabsorbed from the small intestine. Because of coding enterohepatic recirculation, the PBPK model was solved as a system of delay differential equations (DDEs)

The final structure of the model descripted above followed the iterative model development process (incorporating uncertainty and sensitivity analysis) documented in McNally et al. (2021).

Default values for masses and flows, metabolism parameters, partition coefficients were based on literature sources and algorithms (Brown et al. 1997; ICRP 2002; Mazur et al. 2010; Schmitt 2008). Default values for uptake and elimination rates parameters were based on corresponding terms in McNally et al. (2021) and subsequently refined through tuning to available data. The default model assumed 80% of the administered BPA was absorbed through the hepatic route, with a further 5% absorbed through the lymphatic route with the complementary 15% passing through unabsorbed.

.

STATISTICAL ANALYSIS

Parameter Distributions

Probability distributions for tissue volumes, expressed as a fraction of body weight, and tissue blood flows, expressed as a fraction of cardiac output were estimated using a virtual population generated using PopGen (McNally et al. 2014). Specifically, a US population of 10,000 individuals comprised of 2500 Caucasian males; 2500 Caucasian females, 2500 African American males and 2500 African American females, aged 25-45 and with BMI ranging from 19-35 was generated (which captured the characteristics of the human volunteer study population). Probability distributions for tissue volumes and fractional blood flows were based upon statistical analysis of this model output. For other parameters, such as partition coefficients and rate parameters, uniform distributions were ascribed based upon author's judgement to represent conservative yet credible bounds and refined through the model development process. The probability distributions used in the reported uncertainty and sensitivity analyses and parameter calibrations are given in Table A1. The tabulated values are therefore based upon expert judgement and represent conservative yet credible bounding estimates.

Uncertainty Analysis

Uncertainty analysis was conducted throughout the model development process in order to identify coding errors, assess for deficiencies in the current version of the model, and to assess the bounding behaviour of the model as a function of its uncertain parameters; a formalised process for studying model behaviour and thus reducing the likelihood of coding errors is desirable for any PBPK model developed using a bespoke (open source) code (Loizou et al. 2021). An efficient approach using Latin Hypercube sampling was used throughout the model development process. For each new version of the PBPK model a 500-point maxi-min Latin Hypercube design was generated in order to efficiently cover the parameter space defined by the distributions ascribed to model parameter, the PBPK model was run for each of these design points, and various outputs from PBPK models were studied to assess the quantitative behaviour of the current version of the model, and the parameter limits in use at the time. The final uncertainty analysis reported on in this work was based on a 500-point maxi-min Latin Hypercube design and the parameter limits given in Table 2. This analysis used uniform distributions for all parameters, defined by upper and lower limits given in Table A1, with a total of 74 parameters studied. The outputs reported on in this work at the plasma concentrations of BPA, BPAG and BPAS (nM).

Sensitivity Analysis

Sensitivity analysis was conducted throughout the model development process in order to study the key model output sensitivities for each version of the model under development. A two-phased global sensitivity analysis comprising of elementary effects screening followed by a variance-based approach is generally desirable (Loizou et al. 2015; McNally et al. 2011), however during the model development and calibration phase of research, elementary effects screening alone was used to allow a more rapid analysis. In the final analysis reported on in this document the sensitivity analysis focussed on plasma concentrations of BPA, BPAG and BPAS (nM) with results at three time points: 0.5, 2- and 5-hours following ingestion of BPA.

The Morris Test computes two measures of sensitivity: μ^* is the average effect of a parameter on model output; σ is a measure of a non-linear effect of a parameter on the response under study and/or interactions between parameters. For each output under study and at each time point under study the maxima of each of the μ^* and σ over all parameters was determined, and all parameters where μ^* and

σ were within 5% of the maxima were recorded. Any parameter that BPA, BPAG or BPAS were sensitive to, at any time point and for either measure of sensitivity was considered as a variable for taking for forward into calibration – parameters passing this initial filter were subsequently reviewed with decisions for inclusion/exclusion of parameters in the calibration model based on expert judgement. Parameters that did not show sensitivity based upon the within 5% criterion described above were fixed at central values during calibration. This analysis used uniform distributions for all parameters, defined by upper and lower limits given in Table A1, with 74 parameters studied. Five elementary effects were computed for each parameter, leading to a design of 375 model runs.

Calibration

Calibration of a subset of sensitive model parameters using the HBM data of Thayer et al. (2015) was attempted. A Bayesian approach (McNally et al., 2012) was followed. This requires the specification of a joint prior distribution for the parameters under study, which is refined through a comparison of PBPK model predictions and measurements within a statistical model. The resulting (refined) parameter space that is consistent with the prior specification and measurements is the posterior distribution.

In the first instance individual model calibrations were performed using the blood data from each of the 14 volunteers in turn. The final calibration model used in this work used data from all 14 individuals from the HBM study of Thayer et al. (2015) with data on three specific outputs, concentrations of BPA, BPAG and BPAS (nM) in plasma, formally compared within the calibration model.

The final form of the statistical calibration model is given in equations A1-A3, where $\mathrm{CPlasma\ BPA_{ij}}$, $\mathrm{CPlasma\ BPAG_{ij}}$ and $\mathrm{CPlasma\ BPAS_{ij}}$ denote measurement i (at time $\mathrm{t_i}$) for individual j (for j in 1:14) for the three respective model outputs, and $\mu_{\mathrm{BPA}}(\theta, \omega_j)_{ij}$, $\mu_{\mathrm{BPAG}}(\theta, \omega_j)_{ij}$ and $\mu_{\mathrm{BPAS}}(\theta, \omega_j)_{ij}$ denote the predictions from the PBPK model for these outputs corresponding to parameters (θ, ω_j). The vectors θ and ω_j denote the global parameters common to all individuals (suitable for partition coefficients etc.), and participant specific parameters (suitable for accounting for variability in the physiology and modelling the participant specific uptake of BPA etc.). Prediction error for BPA, BPAG and BPAS was modelled through standard deviations σ_{BPA}, σ_{BPAG} and σ_{BPAS} respectively. Normal distributions, truncated at zero were assumed for all three relationships.

$$CPlasma\ BPA_{ij} \sim N\mu_{BPA}(\theta, \omega_j)_{ij}, \sigma_{BPA})[0, \infty] \qquad \text{(A1)}$$

$$CPlasma\ BPAG_{ij} \sim N(\mu_{BPAG}(\theta, \omega_j)_{ij}, \sigma_{BPAG})[0, \infty] \qquad \text{(A2)}$$

$$CPlasma\ BPAS_{ij} \sim N(\mu_{BPAS}(\theta, \omega_j)_{ij}, \sigma_{BPAS})[0, \infty] \qquad \text{(A3)}$$

The prior distributions for model parameters are given in Table A1. Prior distributions for standard deviations were weakly informative half-normal distributions with standard deviations of 10, 250 and 25 for σ_{BPA}, σ_{BPAG} and σ_{BPAS} respectively.

Inference for the model parameters was made using Markov chain Monte Carlo (MCMC). Preliminary calibrations were undertaken using a single component Metropolis-Hastings algorithm. Inference in the final calibration model was made using Markov chain Monte Carlo (MCMC) with a sampling run of 100,000 iterations and every 50th retained for forward inference.

Software

The PBPK model was written using GNU MCSim and compiled. In uncertainty and sensitivity analysis and for runs to produce fits following calibrations the executable was run using scripts called from RStudio. MCMC runs were conducted using GNU MCSim called from a command line environment. The DiceDesign package of R was used for generating Latin Hypercube designs. GSA of model outputs (through elementary effects screening and eFAST) were conducted using the Sensitivity package of R. The reshape2 package of R was used for reshaping of data for plotting and other processing of results.

RESULTS

Assessment of model form

As described in the statistical analysis section an iterative process of model development was conducted with uncertainty and sensitivity analysis informing improvements to model structure. The final model was tested using a 500-point maxi-min Latin Hypercube design and the parameter limits given in Table S1. The simulations corresponding to these design points are shown for concentrations of BPA, BPAS and BPAG in blood (nM) over the period 0-48 hours. The broad range of the runs covered the HBM data reported in Thayer et al. (2015) with the model form considered suitable for the purposes of this work.

Sensitivity analysis

Results from elementary effects screening are reported for BPA, BPAS and BPAG in Tables S2 to S4 for three time points, 0.5, 2 and 5 hours following ingestion of BPA. The mu and sigma measures at each time point are normalised with all results at the time point divided by the maximum value of mu or sigma at that time point; values are therefore between 0 and 1, with 1 denoting the most important for a given measure at a particular time point. The parameters where any of the measures were within 5% of the maximum (i.e. any value above 0.05) are highlighted in bold and considered for inclusion in the calibration model. This analysis was conducted for BPA, BPAS and BPAG.

In total 29 global and a further 11 local (individual specific parameters) were taken forward to calibration.

Calibration

Results from calibration and our interpretation are provided in the main body of the paper, with the fit of the model demonstrated for a subset of 3 of the 14 volunteers (volunteers 1, 3 and 5). A similar comparison of model predictions (central estimate and a pointwise 95% credible interval) and measurements is made for the remaining 11 volunteers in Figures S2 to S12, thus demonstrating an excellent fit to all of the volunteers following calibration.

Table S1 **Parameter distributions (and upper and lower bounds) used in Latin Hypercube sampling, Elementary effects screening sensitivity analysis and as prior distributions in calibration of sensitive parameters.**

Parameter	Unit	Mean	SD	Lower bound	Upper bound	Distribution
**BW**	kg	4.36	0.313	3.747	4.973	Lognormal
**VliC**	L kg^{-1} BW	0.0307	0.00758	0.02	0.05	Normal
**VstC**	L kg^{-1} BW	0.0210	0.00069	0.021	0.0235	Normal
**VguC**	L kg^{-1} BW	0.0150	0.00234	0.008	0.0220	Normal
**VkiC**	L kg^{-1} BW	0.0038	0.00148	0.0012	0.005	Normal
**VlymphC**	L kg^{-1} BW	0.0036	0.0007	0.0022	0.0050	Normal
**VfaC**	L kg^{-1} BW	0.27	0.0600	0.1500	0.39	Normal
**VspdC**	L kg^{-1} BW	0.6050	0.1000	0.4500	0.7500	Normal
**VrpdC**	L kg^{-1} BW	0.0302	0.005	0.010	0.0454	Normal
**VBldC**	L kg^{-1} BW	0.060	0.01	0.04	0.09	Normal
**QCC**	L h^{-1} Kg^{-1} $BW^{0.75}$	11	1	9	13	Normal
**QhepartC**	Unit less	0.0690	0.0060	0.03	0.12	Normal
**QstC**	Unit less	0.0110	0.0009	0.005	0.015	Normal
**QguC**	Unit less	0.1490	0.0130	0.09	0.25	Normal
**QkiC**	Unit less	0.20	0.05	0.10	0.30	Normal
**QfaC**	Unit less	0.0500	0.0050	0.0300	0.0699	Normal
**QspdC**	Unit less	0.2870	0.0221	0.2100	0.3600	Normal
**QrpdC**	Unit less	0.2100	0.0168	0.1600	0.2700	Normal
**FracDOSEHep**	Unit less	-	-	0.7	0.92	Uniform
**FracDOSELymph**	Unit less	-	-	0.02	0.08	Uniform
**FB_BPA**	Unit less	-	-	0	0.99	Uniform
**FB_BPAG**	Unit less	-	-	0.7	0.99	Uniform
**FB_BPAS**	Unit less	-	-	0.7	0.99	Uniform
**MPY**	mg/g	34	10	14	54	Normal
**MPYgu**	mg/g	3.9	0.8	2.3	5.5	Normal
**BELLYPERM**	h^{-1}	-	-	0.1	10	Uniform
**GIPERM**	h^{-1}	-	-	0.5	25	Uniform
**BELLYPERMLymph**	h^{-1}	-	-	0.84	2.5	Uniform
**GIPERMLymph**	h^{-1}	-	-	0.55	1.6	Uniform
**KEMAX**	h^{-1}	-	-	0.1	25	Uniform
**KEMIN**	h^{-1}	-	-	0.0025	0.0075	Uniform
**K1_BPA_GUT**	h^{-1}	-	-	0.01	20	Uniform
**K1_BPAG_GUT**	h^{-1}	-	-	0.01	20	Uniform
**K1_BPAS_GUT**	h^{-1}	-	-	0.01	20	Uniform
**K1_BPA_LIVER**	h^{-1}	-	-	0.55	1.6	Uniform
**K1_BPAG_LIVER**	h^{-1}	-	-	0.005	0.015	Uniform
**K1_BPAS_LIVER**	h^{-1}	-	-	0.005	0.015	Uniform
**K1_BPA_Urine**	h^{-1}	-	-	0.0005	0.0015	Uniform
**K1_BPAG_Urine**	h^{-1}	-	-	0.0005	0.0015	Uniform
**K1_BPAS_Urine**	h^{-1}	-	-	0.0005	0.0015	Uniform
**K1_BPA_REMOVED_PLASMA**	h^{-1}		-	0.01	100	Uniform
**K1_BPAG_REMOVED_PLASMA**	h^{-1}		-	10	150	Uniform
**K1_BPAS_REMOVED_PLASMA**	h^{-1}		-	0.01	100	Uniform
**K1Lymph**	h^{-1}	-	-	0.25	0.75	Uniform
**Lymphlag**	h^{-1}	-	-	0.25	1.25	Uniform

**Vmax_liv_BPA_in_vitro**	pmol/min/mg	4255	900	1000	8000	Normal
**KM_liv_BPA_in_vitro**	mg/L	1.118	0.20	0.1	2.5	Normal
**Vmax_liv_BPAS_in_vitro**	pmol/min/mg	80	30	13	133	Normal
**KM_liv_BPAS_in_vitro**	mg/L	3.114	0.6	1.5	6.5	Normal
**Vmax_gut_BPAG_in_vitro**	pmol/min/mg	487	100	244	974	Normal
**KM_gut_BPAG_in_vitro**	mg/L	18.29	4	9	37	Normal
**Vmax_gut_BPAS_in_vitro**	pmol/min/mg	73	30	13	133	Normal
**KM_gut_BPAS_in_vitro**	mg/L	3.114	0.6	1.5	6.5	Normal
**Pbab**	Unit less	-	-	0.36	1.1	Uniform
**Plib**	Unit less	-	-	0.36	1.1	Uniform
**Pkib**	Unit less	-	-	1.35	15	Uniform
**Pfab**	Unit less	-	-	1.35	15	Uniform
**Pgub**	Unit less	-	-	1.35	15	Uniform
**Pstb**	Unit less	-	-	1.35	15	Uniform
**Prpdb**	Unit less	-	-	1.4	4.2	Uniform
**Pspdb**	Unit less	-	-	1.4	4.2	Uniform
**PbaG**	Unit less	-	-	0.7	2.1	Uniform
**PliG**	Unit less	-	-	1.0	23	Uniform
**PkiG**	Unit less	-	-	1.0	23	Uniform
**PfaG**	Unit less	-	-	1.2	3.60	Uniform
**PguG**	Unit less	-	-	1.0	23	Uniform
**PstG**	Unit less	-	-	1.70	5.3	Uniform
**PrpdG**	Unit less	-	-	2.1	6.4	Uniform
**PspdG**	Unit less	-	-	1	3	Uniform
**PbaS**	Unit less	-	-	0.7	2.1	Uniform
**PliS**	Unit less	-	-	1.0	23	Uniform
**PkiS**	Unit less	-	-	1.0	23	Uniform
**PfaS**	Unit less	-	-	1.3	3.9	Uniform
**PguS**	Unit less	-	-	1.0	23	Uniform
**PstS**	Unit less	-	-	1.9	5.7	Uniform
**PrpdS**	Unit less	-	-	2.3	6.8	Uniform
**PspdS**	Unit less	-	-	1	3.1	Uniform

Table S2 **Results (normalised mu and sigma) from elementary effects screening of BPA at three time points. Parameters satisfying the criteria for further consideration are highlighted in bold.**

	BPA					
	mu 0.5	sigma 0.5	mu 2.0	sigma 2.0	mu 5.0	sigma 5.0
**QCC**	0.0553	0.0713	0.0314	0.0545	0.0353	0.0637
VfaC	0.1303	0.1427	0.0457	0.0564	0.0102	0.0142
VguC	0.0552	0.0414	0.0108	0.0186	0.0162	0.0287
VstC	0.0015	0.0026	0.0002	0.0002	0.0004	0.0005
VspdC	0.0473	0.0814	0.0277	0.0525	0.0167	0.0325
VrpdC	0.0096	0.0126	0.0026	0.0042	0.0064	0.007
**VkiC**	0.0067	0.0057	0.0304	0.0348	0.0931	0.1154
**VliC**	0.0314	0.0501	0.0042	0.0049	0.0067	0.0088
VlymphC	0.0002	0.0001	0	0	0	0
**VBldC**	0.2111	0.4171	0.0885	0.1684	0.0334	0.0649
QhepartC	0.0202	0.0311	0.0158	0.0242	0.0738	0.1209
**QguC**	0.0364	0.069	0.0078	0.0092	0.0417	0.0715
**QstC**	0.1056	0.2033	0.0044	0.0079	0.0085	0.0103
QspdC	0.0276	0.0246	0.0039	0.0029	0.0509	0.0907
QrpdC	0.09	0.169	0.0016	0.0006	0.0121	0.0198
**QkiC**	0.0069	0.0093	0.0281	0.0482	0.0478	0.0859
QfaC	0.0186	0.0165	0.0035	0.0045	0.0049	0.0081
**FracDOSELymph**	0.282	0.3197	0.3931	0.4466	0.538	0.5834
**FracDOSEHep**	0.0425	0.059	0.0092	0.0103	0.0122	0.0166
**FB_BPA**	1	1	1	1	1	1
**FB_BPAG**	0	0	0	0	0	0
**FB_BPAS**	0	0	0	0	0	0
**BELLYPERM**	0.3124	0.608	0.0229	0.0416	0.0948	0.185
**GIPERM**	0.2675	0.3855	0.008	0.0126	0.006	0.0071
**KEMAX**	0.1813	0.2626	0.0074	0.0113	0.0173	0.0276
KEMIN	0.0003	0.0005	0	0	0.0001	0.0002
**MPY**	0.1722	0.203	0.012	0.0159	0.0126	0.0168
MPYgu	0.0037	0.0065	0.0003	0.0003	0.0003	0.0003
**K1_BPA_REMOVED_PLASMA**	0.0146	0.0152	0.0408	0.0528	0.128	0.148
**K1_BPAG_REMOVED_PLASMA**	0	0	0	0	0	0
**K1_BPAS_REMOVED_PLASMA**	0	0	0	0	0	0
**K1_BPA_GUT**	0.0927	0.1095	0.0068	0.0075	0.0071	0.0076
K1_BPA_LIVER	0.0007	0.0014	0	0.0001	0.0001	0.0001
**K1_BPAG_GUT**	0	0	0	0	0	0
K1_BPAG_LIVER	0	0	0	0	0	0
K1_BPAS_GUT	0	0	0	0	0	0
K1_BPAS_LIVER	0	0	0	0	0	0
Pbab	0	0	0	0	0	0
Pfab	0.0006	0.0008	0.0056	0.0076	0.0517	0.0775
**Pgub**	0.0212	0.0211	0.0005	0.0007	0.0009	0.0013
**Pstb**	0.0487	0.0637	0.0079	0.0142	0.001	0.0008
Prpdb	0.0075	0.0048	0.0064	0.0106	0.0032	0.0042
Pspdb	0.0065	0.0068	0.0372	0.0351	0.1002	0.1401
Plib	0	0.0001	0	0	0	0
PbaG	0	0	0	0	0	0
PspdG	0	0	0	0	0	0
**PliG**	0	0	0	0	0	0
PrpdG	0	0	0	0	0	0
PfaG	0	0	0	0	0	0
PstG	0	0	0	0	0	0
**PguG**	0	0	0	0	0	0
PbaS	0	0	0	0	0	0
PspdS	0	0	0	0	0	0
**PliS**	0	0	0	0	0	0
PrpdS	0	0	0	0	0	0

PfaS	0	0	0	0	0	0
PstS	0	0	0	0	0	0
**PguS**	0	0	0	0	0	0
Pkib	0.0093	0.0096	0.0195	0.0223	0.0504	0.0576
**PkiG**	0	0	0	0	0	0
**PkiS**	0	0	0	0	0	0
**Vmax_liv_BPA_in_vitro**	0.5204	0.8879	0.1174	0.1563	0.2077	0.295
**KM_liv_BPA_in_vitro**	0.7788	0.6316	0.0854	0.0416	0.1178	0.0912
**Vmax_gut_BPAG_in_vitro**	0.0024	0.0022	0.0005	0.0005	0.0006	0.0006
**KM_gut_BPAG_in_vitro**	0.0054	0.0092	0.0008	0.0009	0.0007	0.0008
**Vmax_liv_BPAS_in_vitro**	0.0001	0.0001	0	0	0	0
**KM_liv_BPAS_in_vitro**	0	0	0	0	0	0
**Vmax_gut_BPAS_in_vitro**	0.0044	0.0076	0.0006	0.0011	0.0008	0.0013
**KM_gut_BPAS_in_vitro**	0.0008	0.0009	0.0001	0.0001	0.0001	0.0001
K1_BPA_Urine	0	0	0	0	0	0
K1_BPAG_Urine	0	0	0	0	0	0
K1_BPAS_Urine	0	0	0	0	0	0
**Lymphlag**	0.3377	0.2802	0.0161	0.0219	0.0316	0.0351
**K1Lymph**	0.2652	0.3801	0.1045	0.1857	0.0725	0.123

Table S3 **Results (normalised mu and sigma) from elementary effects screening of BPAS at three time points. Parameters satisfying the criteria for further consideration are highlighted in bold.**

	BPAS					
	mu 0.5	sigma 0.5	mu 2.0	sigma 2.0	mu 5.0	sigma 5.0
**QCC**	0.0513	0.0396	0.0372	0.0203	0.0411	0.0374
VfaC	0.0524	0.0198	0.0241	0.0226	0.0177	0.025
VguC	0.0252	0.0058	0.0146	0.0104	0.0055	0.0021
VstC	0.0009	0.0006	0.0004	0.0003	0.0002	0.0003
VspdC	0.1257	0.0558	0.0484	0.0375	0.0164	0.0202
VrpdC	0.0186	0.0116	0.0092	0.0064	0.0054	0.0069
**VkiC**	0.0043	0.0019	0.0144	0.0084	0.0473	0.057
**VliC**	0.0959	0.054	0.0394	0.0186	0.018	0.0168
VlymphC	0.0026	0.0017	0.0011	0.0006	0.0005	0.0004
**VBldC**	0.188	0.1233	0.1003	0.0897	0.0553	0.0794
QhepartC	0.0339	0.0188	0.016	0.0039	0.0087	0.0033
**QguC**	0.2096	0.1371	0.1087	0.088	0.1535	0.1598
**QstC**	0.0327	0.0305	0.003	0.0022	0.0028	0.0027
QspdC	0.0544	0.0218	0.0266	0.0151	0.0459	0.0404
QrpdC	0.0251	0.0133	0.0224	0.0137	0.0241	0.0266
**QkiC**	0.0661	0.0339	0.095	0.0776	0.1185	0.1361
QfaC	0.0114	0.005	0.004	0.002	0.0051	0.0041
**FracDOSELymph**	0	0	0.0015	0.0009	0.0032	0.0025
**FracDOSEHep**	0.0525	0.0184	0.0354	0.0147	0.0249	0.0179
**FB_BPA**	0.0002	0.0001	0.0026	0.0014	0.0009	0.0008
**FB_BPAG**	0	0	0	0	0	0
**FB_BPAS**	0.5243	0.2533	0.878	0.4307	1	1
**BELLYPERM**	0.0738	0.0454	0.1008	0.0887	0.0881	0.1281
**GIPERM**	0.8571	0.7807	0.5226	0.5389	0.203	0.3009
**KEMAX**	0.1951	0.0985	0.1508	0.1274	0.1346	0.1797
KEMIN	0.0001	0.0001	0	0	0	0
**MPY**	0.6095	0.5458	0.5632	0.5235	0.3948	0.5086
MPYgu	0.0073	0.0078	0.01	0.0088	0.0065	0.0067
**K1_BPA_REMOVED_PLASMA**	0	0	0.0004	0.0002	0.001	0.0007
**K1_BPAG_REMOVED_PLASMA**	0	0	0	0	0	0
**K1_BPAS_REMOVED_PLASMA**	0.0257	0.0236	0.0999	0.0732	0.162	0.1545
**K1_BPA_GUT**	0.2598	0.064	0.2382	0.1283	0.2026	0.1943
K1_BPA_LIVER	0.0002	0.0002	0.0001	0.0001	0	0

**K1_BPAG_GUT**	0	0	0	0	0	0
K1_BPAG_LIVER	0	0	0	0	0	0
K1_BPAS_GUT	0.004	0.0031	0.2118	0.0916	0.3252	0.2744
K1_BPAS_LIVER	0.0004	0.0002	0.0013	0.0004	0.0014	0.0013
Pbab	0	0	0	0	0	0
Pfab	0	0	0	0	0.0001	0
**Pgub**	0.1272	0.0466	0.2469	0.1654	0.2831	0.3199
**Pstb**	0.0083	0.0042	0.0037	0.0023	0.0006	0.0005
Prpdb	0	0	0.0001	0.0001	0	0
Pspdb	0	0	0.0002	0.0001	0.0004	0.0004
Plib	0	0	0	0	0	0
PbaG	0	0	0	0	0	0
PspdG	0	0	0	0	0	0
**PliG**	0	0	0	0	0	0
PrpdG	0	0	0	0	0	0
PfaG	0	0	0	0	0	0
PstG	0	0	0	0	0	0
**PguG**	0	0	0	0	0	0
PbaS	0	0	0	0	0	0
PspdS	0.0108	0.0075	0.0369	0.0168	0.0486	0.0683
**PliS**	0.3208	0.1836	0.0775	0.0576	0.024	0.0257
PrpdS	0.0057	0.0014	0.0026	0.0016	0.0007	0.0008
PfaS	0.0003	0.0001	0.0056	0.0021	0.0123	0.0106
PstS	0	0	0.0006	0.0001	0.0016	0.0014
**PguS**	0.4869	0.1888	0.2018	0.1327	0.1595	0.2231
Pkib	0	0	0.0002	0.0001	0.0003	0.0004
**PkiG**	0	0	0	0	0	0
**PkiS**	0.0305	0.0136	0.1012	0.0518	0.1572	0.1272
**Vmax_liv_BPA_in_vitro**	0.0463	0.0276	0.0182	0.006	0.0193	0.0094
**KM_liv_BPA_in_vitro**	1	1	1	1	0.5443	0.7893
**Vmax_gut_BPAG_in_vitro**	0.0041	0.0021	0.005	0.0016	0.0051	0.0039
**KM_gut_BPAG_in_vitro**	0.0047	0.002	0.006	0.0026	0.0062	0.0045
**Vmax_liv_BPAS_in_vitro**	0.0212	0.0067	0.0152	0.008	0.0158	0.013
**KM_liv_BPAS_in_vitro**	0.0036	0.0019	0.007	0.0028	0.0091	0.0052
**Vmax_gut_BPAS_in_vitro**	0.3133	0.1431	0.1265	0.0426	0.1053	0.0666
**KM_gut_BPAS_in_vitro**	0.1844	0.1108	0.4211	0.2849	0.5298	0.5886
K1_BPA_Urine	0	0	0	0	0	0
K1_BPAG_Urine	0	0	0	0	0	0
K1_BPAS_Urine	0	0	0	0	0	0
**Lymphlag**	0.0562	0.0572	0.0524	0.0462	0.0112	0.0107
**K1Lymph**	0.0001	0	0.0064	0.0037	0.0031	0.002

Table S4 **Results (normalised mu and sigma values) from elementary effects screening of BPAG at three time points. Parameters satisfying the criteria for further consideration are highlighted in bold.**

	BPAG					
	mu 0.5	sigma 0.5	mu 2.0	sigma 2.0	mu 5.0	sigma 5.0
**QCC**	0.0328	0.0395	0.0467	0.1349	0.0848	0.1359
VfaC	0.1433	0.1694	0.1199	0.0757	0.0744	0.098
VguC	0.0428	0.0252	0.0916	0.1012	0.1152	0.1069
VstC	0.0012	0.0013	0.0034	0.0017	0.0022	0.0036
VspdC	0.1578	0.125	0.0769	0.2857	0.0874	0.1029
VrpdC	0.0232	0.0305	0.0206	0.0584	0.013	0.0163
**VkiC**	0.0246	0.0386	0.1913	0.5072	0.2376	0.3121
**VliC**	0.079	0.1099	0.1362	0.3996	0.0528	0.1123
VlymphC	0.0031	0.0032	0.0032	0.0059	0.0016	0.0026
**VBldC**	0.0794	0.1035	0.0215	0.0726	0.0324	0.0409
QhepartC	0.0165	0.0197	0.0219	0.0495	0.0256	0.0448

**QguC**	0.1508	0.1242	0.0606	0.1793	0.1321	0.3144
**QstC**	0.0531	0.0568	0.0596	0.1378	0.0879	0.1688
QspdC	0.1078	0.0604	0.1269	0.3083	0.0901	0.0636
QrpdC	0.0336	0.0242	0.0312	0.0677	0.0337	0.0483
**QkiC**	0.0863	0.089	0.2057	0.4693	0.3035	0.5814
QfaC	0.0301	0.042	0.0645	0.1858	0.0341	0.0517
**FracDOSELymph**	0	0.0001	0.0251	0.0569	0.0399	0.0453
**FracDOSEHep**	0.0411	0.0134	0.2038	0.2595	0.2422	0.2436
**FB_BPA**	0.0019	0.0029	0.0187	0.0583	0.0053	0.013
**FB_BPAG**	0.5948	0.6454	1	0.9287	1	0.6251
**FB_BPAS**	0	0	0	0	0	0
**BELLYPERM**	0.1984	0.1959	0.1894	0.7073	0.2035	0.3176
**GIPERM**	1	1	0.2685	0.5515	0.2031	0.3273
**KEMAX**	0.2945	0.2712	0.2491	0.9648	0.2355	0.3533
KEMIN	0.0003	0.0005	0	0.0001	0.0001	0.0003
**MPY**	0.0241	0.0407	0.0179	0.0371	0.0184	0.0259
MPYgu	0.001	0.0016	0.0102	0.0393	0.0133	0.0346
**K1_BPA_REMOVED_PLASMA**	0.0001	0.0002	0.0034	0.0081	0.0065	0.0078
**K1_BPAG_REMOVED_PLASMA**	0.0216	0.0244	0.1907	0.2892	0.4365	0.557
**K1_BPAS_REMOVED_PLASMA**	0	0	0	0	0	0
**K1_BPA_GUT**	0.2879	0.1815	0.3078	0.8421	0.2872	0.6714
K1_BPA_LIVER	0.0002	0.0003	0.0008	0.0028	0.0005	0.001
**K1_BPAG_GUT**	0.0019	0.0025	0.3342	1	0.5865	1
K1_BPAG_LIVER	0.0009	0.0009	0.0027	0.0042	0.002	0.0036
K1_BPAS_GUT	0	0	0	0	0	0
K1_BPAS_LIVER	0	0	0	0	0	0
Pbab	0	0	0	0	0	0
Pfab	0	0	0.0002	0.0005	0.0023	0.0033
**Pgub**	0.1747	0.2164	0.2024	0.4539	0.1599	0.3026
**Pstb**	0.0321	0.0305	0.1258	0.2758	0.0272	0.0396
Prpdb	0	0	0.0007	0.0016	0.0002	0.0006
Pspdb	0	0	0.0045	0.0087	0.0094	0.0112
Plib	0.0002	0.0003	0.0003	0.0012	0.0002	0.0006
PbaG	0	0	0	0	0	0
PspdG	0.0215	0.027	0.1418	0.2366	0.0594	0.11
**PliG**	0.5366	0.5089	0.1545	0.4385	0.1032	0.1742
PrpdG	0.0422	0.0475	0.0225	0.0722	0.023	0.0459
PfaG	0.0014	0.0014	0.0282	0.0411	0.0485	0.0569
PstG	0.0001	0.0001	0.0107	0.0181	0.0111	0.0164
**PguG**	0.0046	0.0039	0.0751	0.14	0.1471	0.2405
PbaS	0	0	0	0	0	0
PspdS	0	0	0	0	0	0
**PliS**	0	0	0	0	0	0
PrpdS	0	0	0	0	0	0
PfaS	0	0	0	0	0	0
PstS	0	0	0	0	0	0
**PguS**	0	0	0	0	0	0
Pkib	0.0001	0.0002	0.0044	0.0209	0.0061	0.0166
**PkiG**	0.0248	0.0249	0.1144	0.1984	0.2379	0.2733
**PkiS**	0	0	0	0	0	0
**Vmax_liv_BPA_in_vitro**	0.0114	0.013	0.0226	0.0355	0.0186	0.0197
**KM_liv_BPA_in_vitro**	0.035	0.0452	0.0517	0.138	0.0383	0.0938
**Vmax_gut_BPAG_in_vitro**	0.0038	0.0076	0.0136	0.06	0.0188	0.0553
**KM_gut_BPAG_in_vitro**	0.0018	0.0028	0.0152	0.0671	0.0213	0.0616
**Vmax_liv_BPAS_in_vitro**	0.0009	0.0007	0.0013	0.0027	0.0014	0.0019
**KM_liv_BPAS_in_vitro**	0.0003	0.0003	0.0024	0.007	0.0028	0.005
**Vmax_gut_BPAS_in_vitro**	0.0349	0.0603	0.0393	0.073	0.0385	0.0438
**KM_gut_BPAS_in_vitro**	0.0058	0.0043	0.0239	0.069	0.0262	0.0588
K1_BPA_Urine	0	0	0	0	0	0
K1_BPAG_Urine	0	0	0	0	0	0

K1_BPAS_Urine	0	0	0	0	0	0
**Lymphlag**	0.0019	0.004	0.0058	0.0187	0.0021	0.002
**K1Lymph**	0.0002	0.0004	0.019	0.0469	0.0061	0.0154

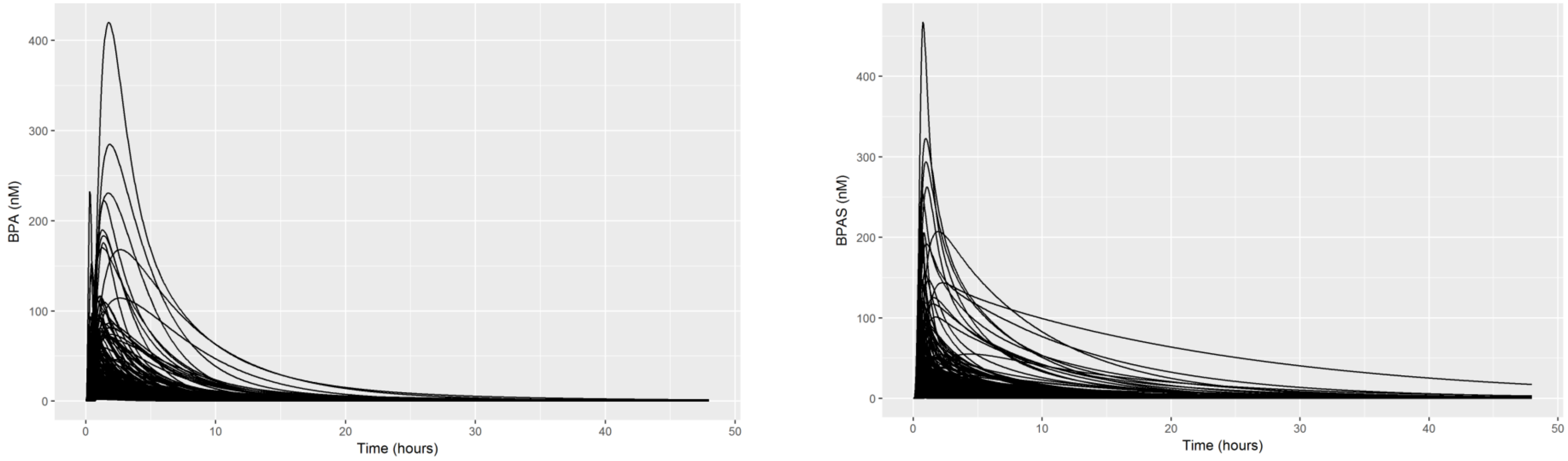

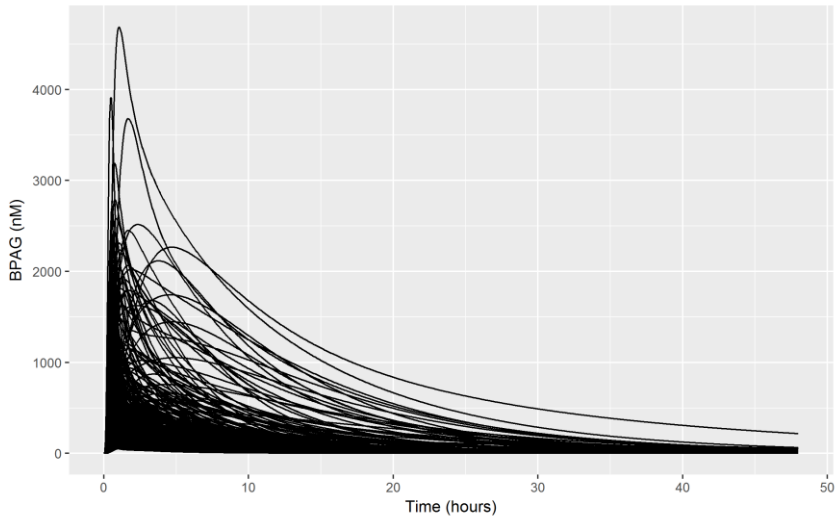

Figure S1 Uncertainty analysis results for BPA, BPAG and BPAS

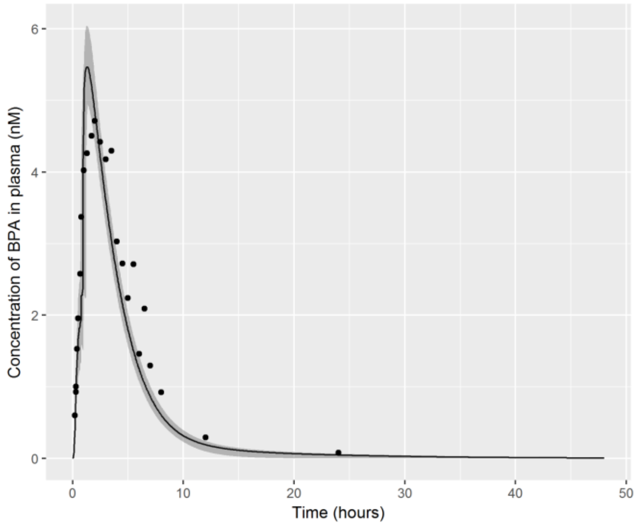

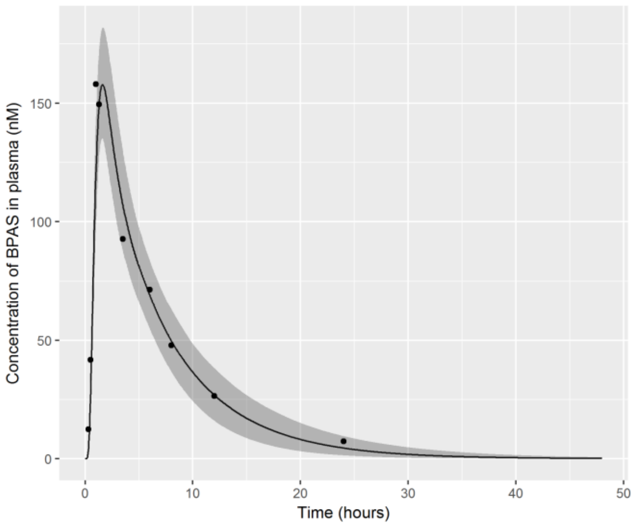

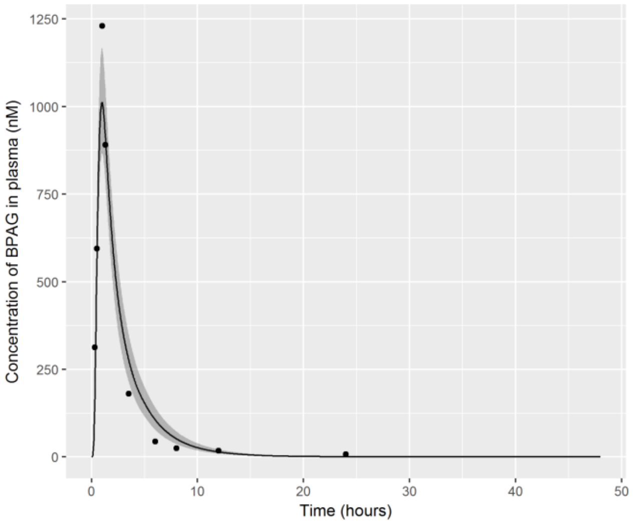

Figure S2 Individual 2

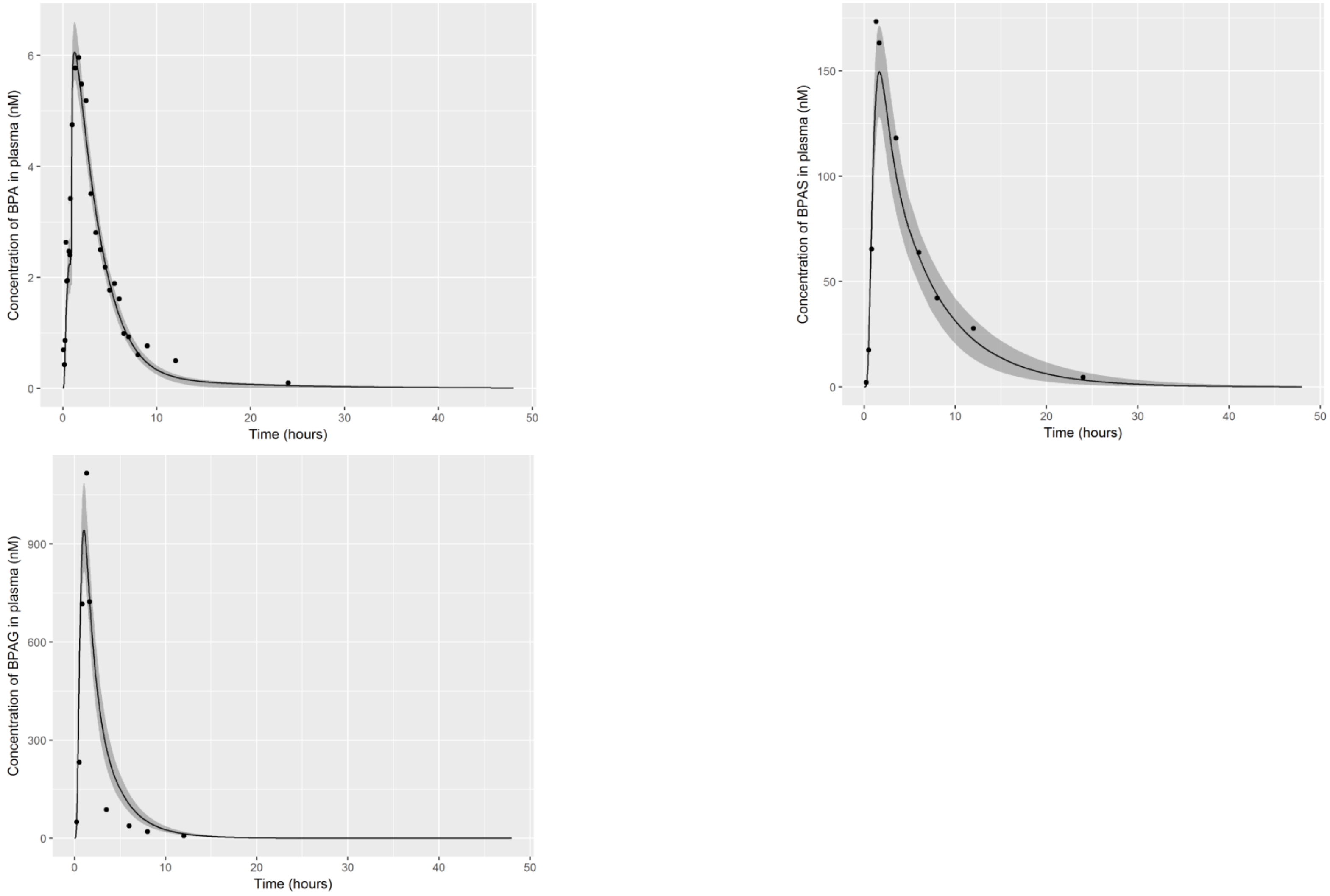

Figure S3 Individual 4

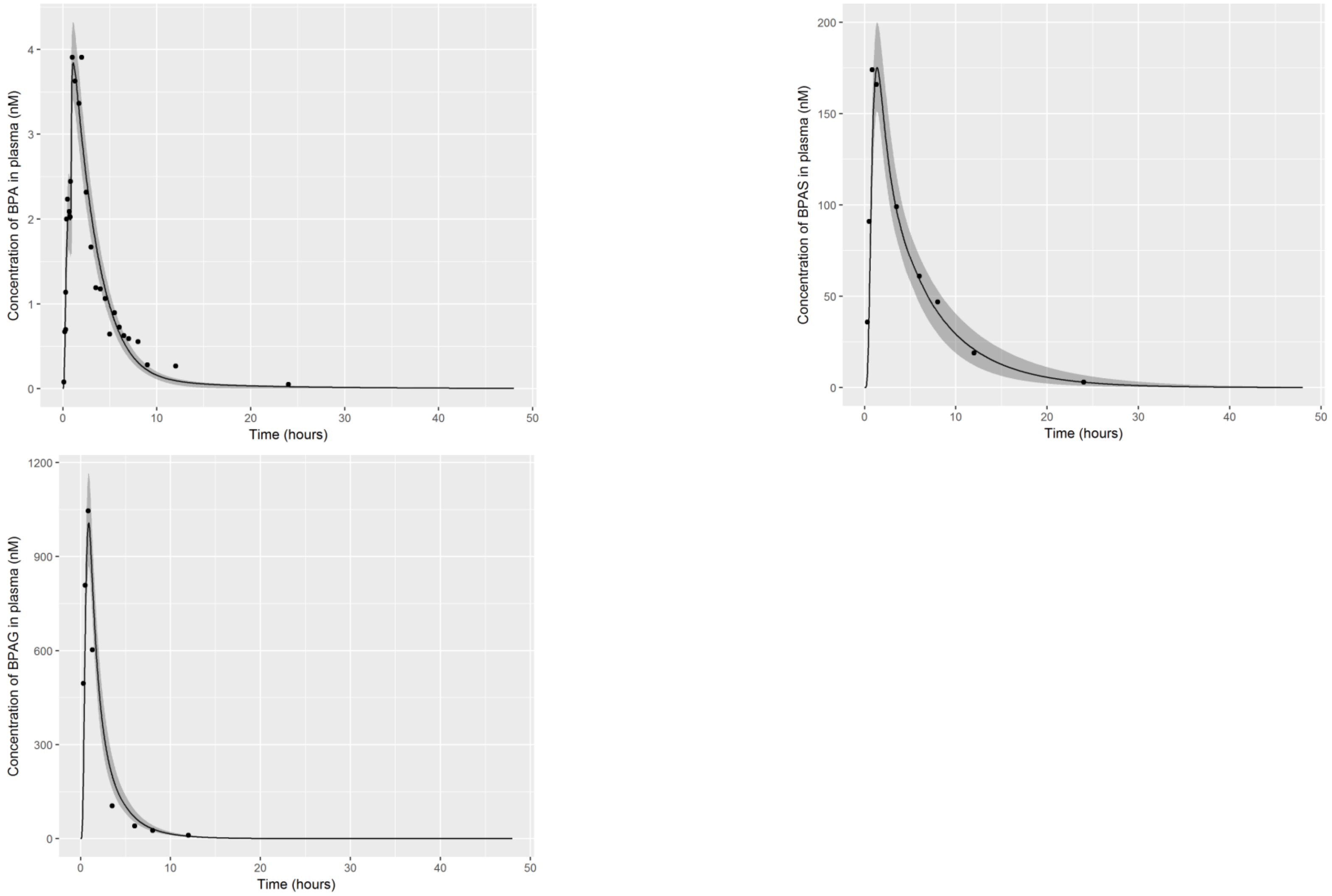

Figure S4 Individual 6

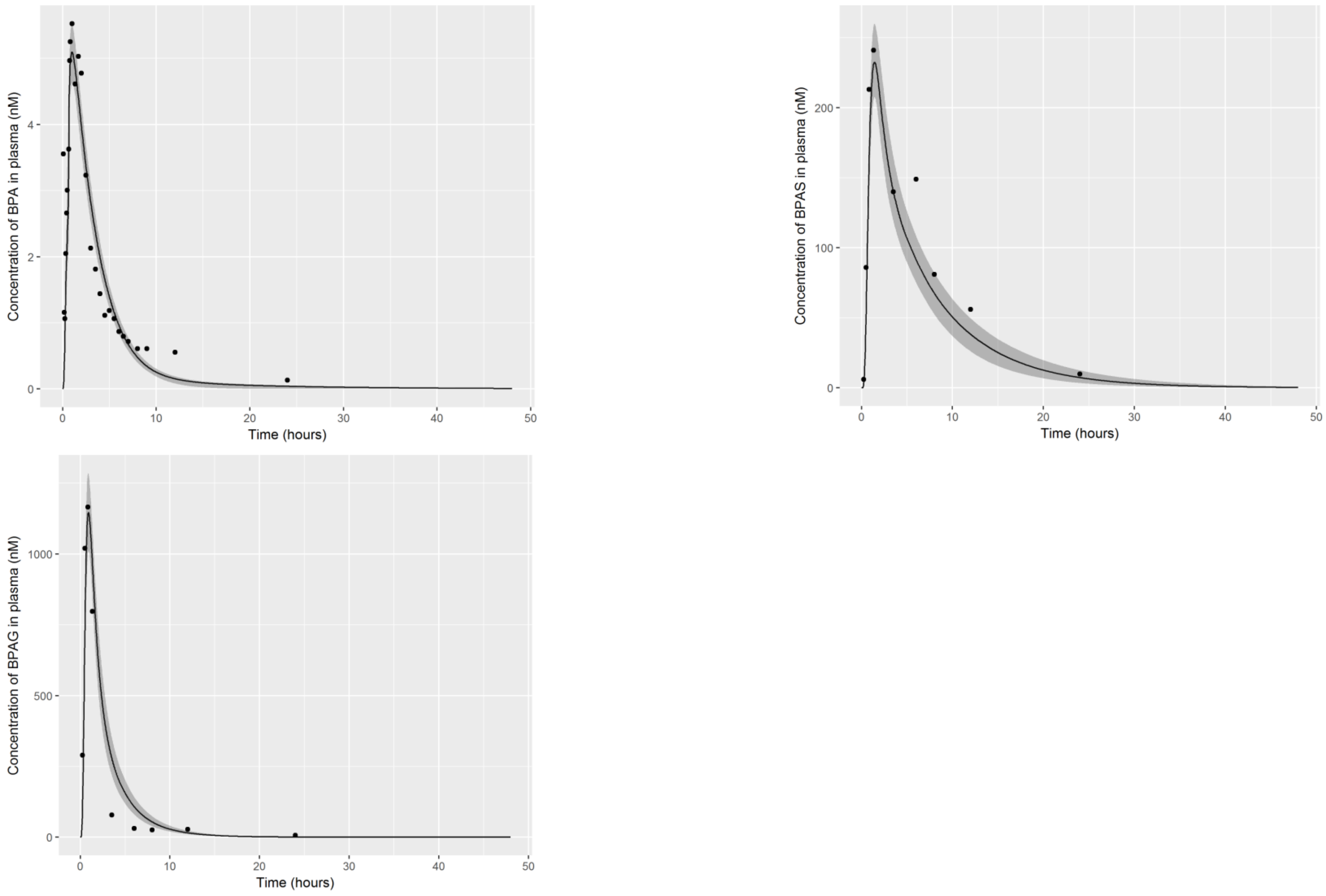

Figure S5 Individual 7

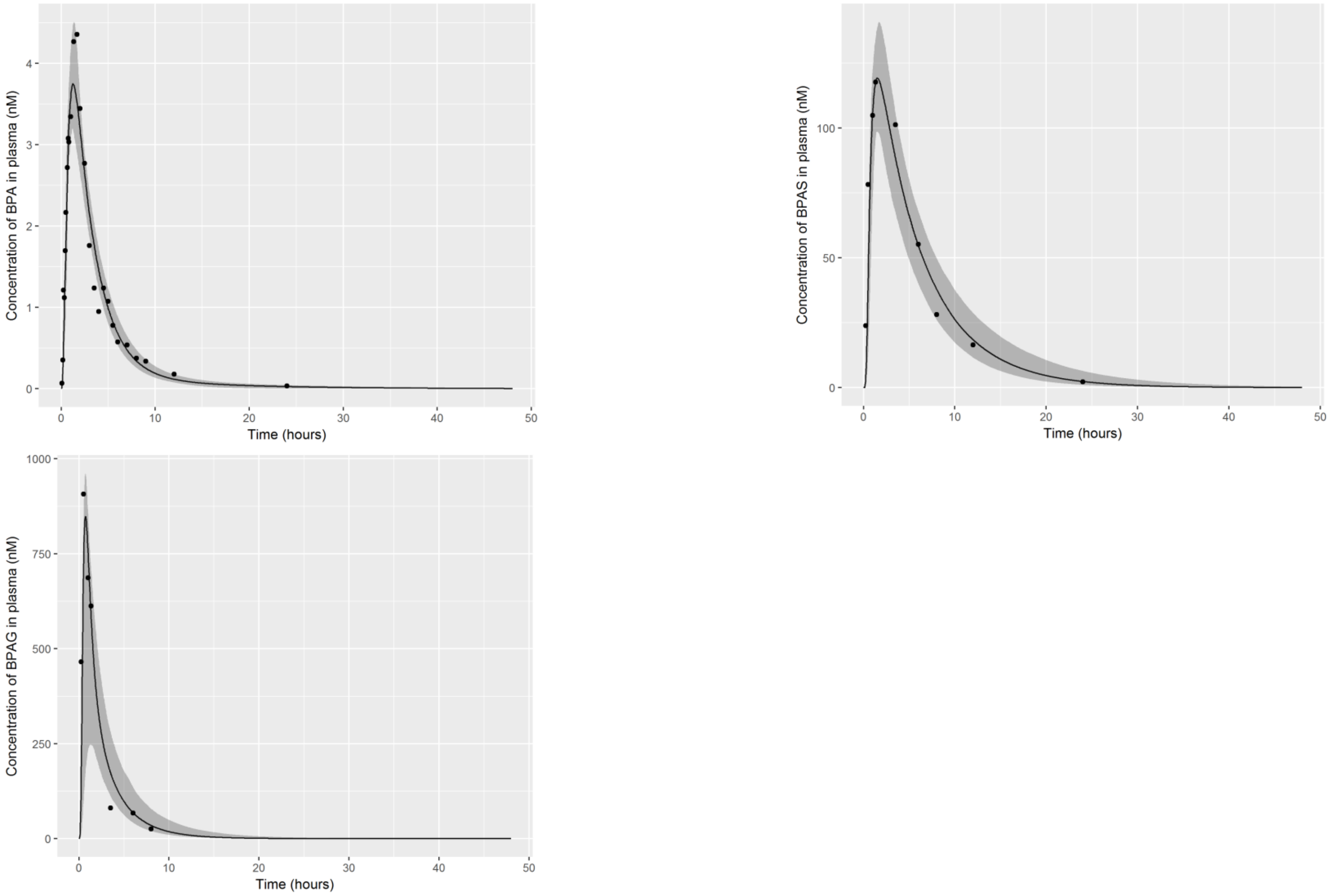

Figure S6 Individual 8

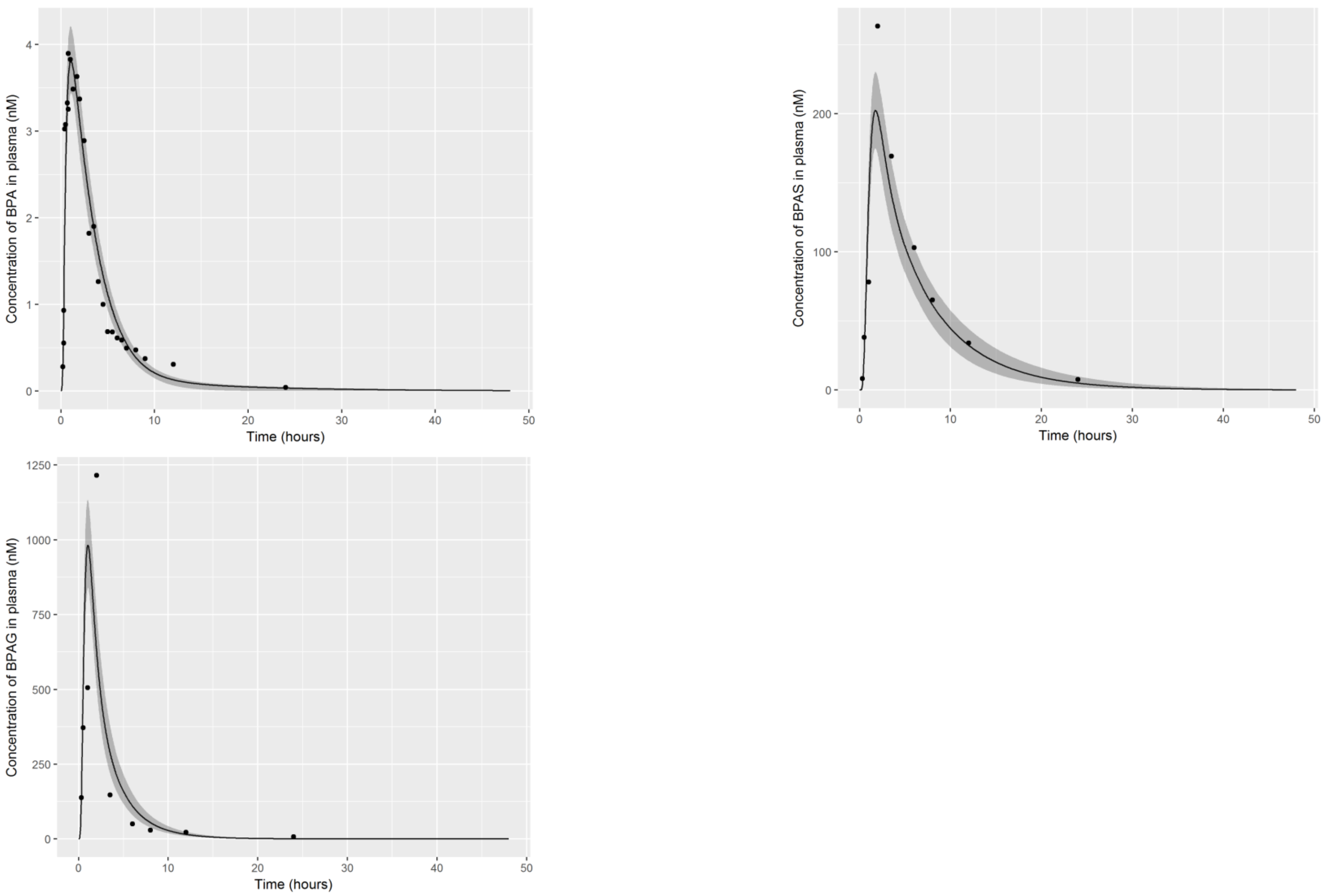

Figure S7 Individual 9

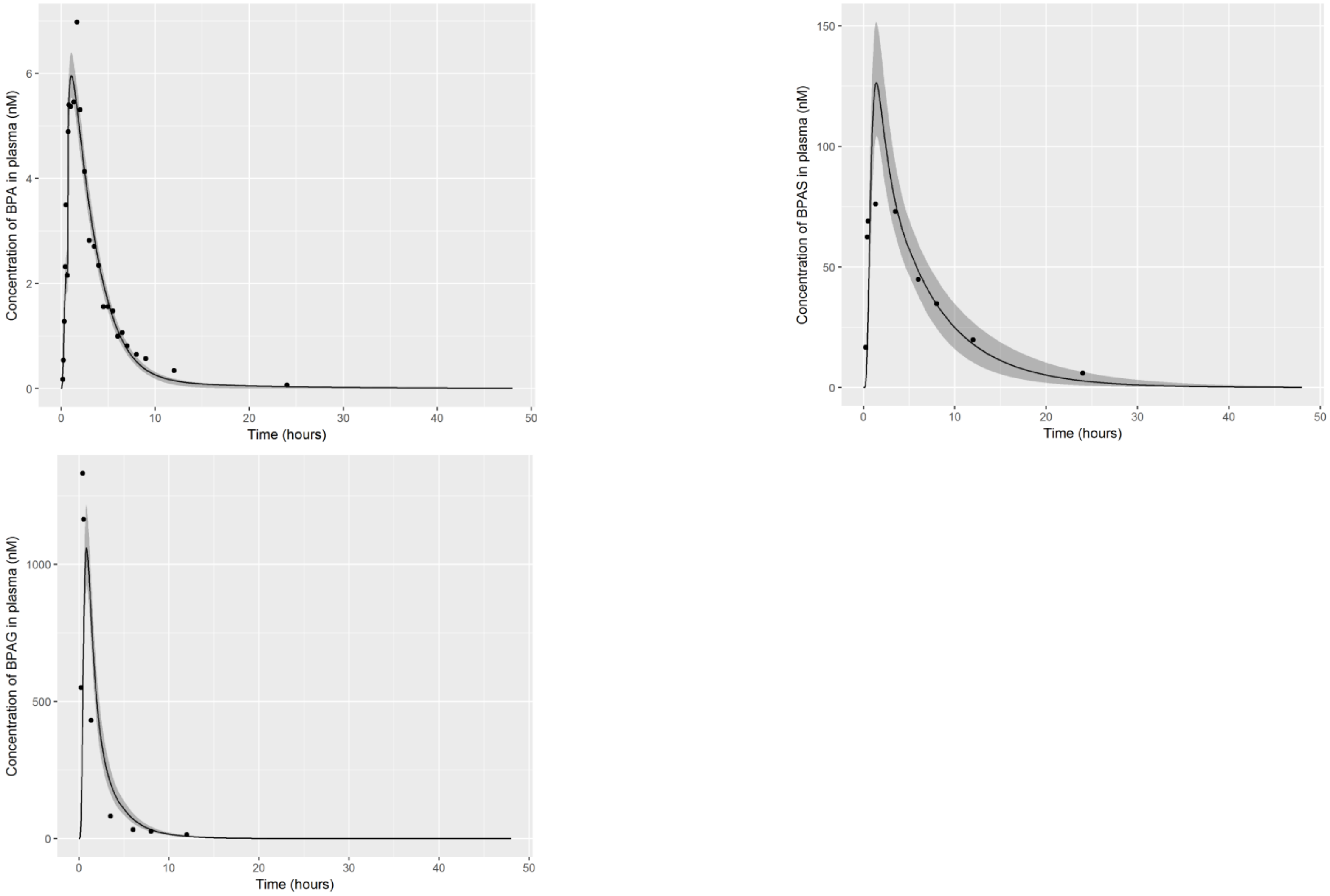

Figure S8 Individual 10

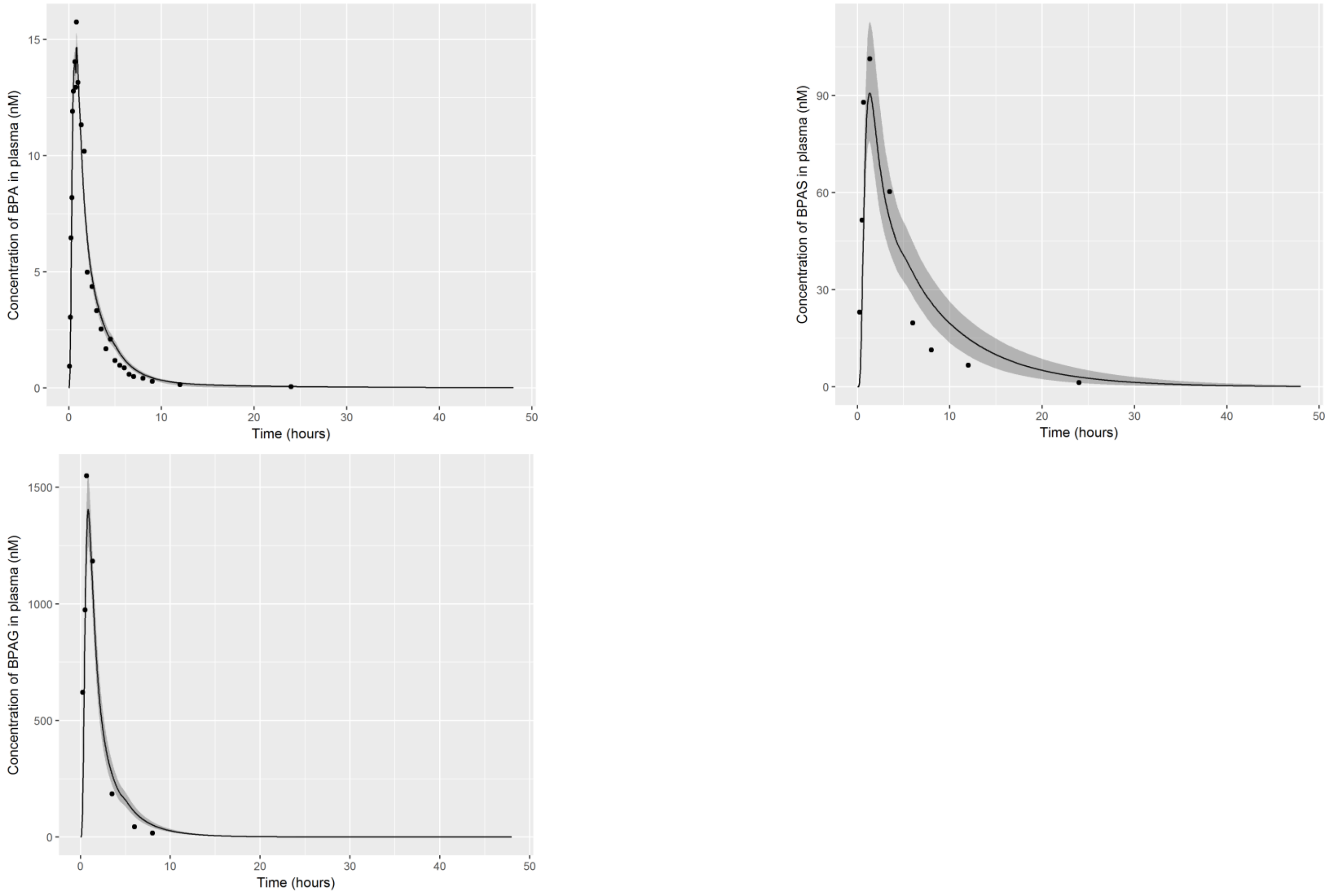

Figure S9 Individual 11

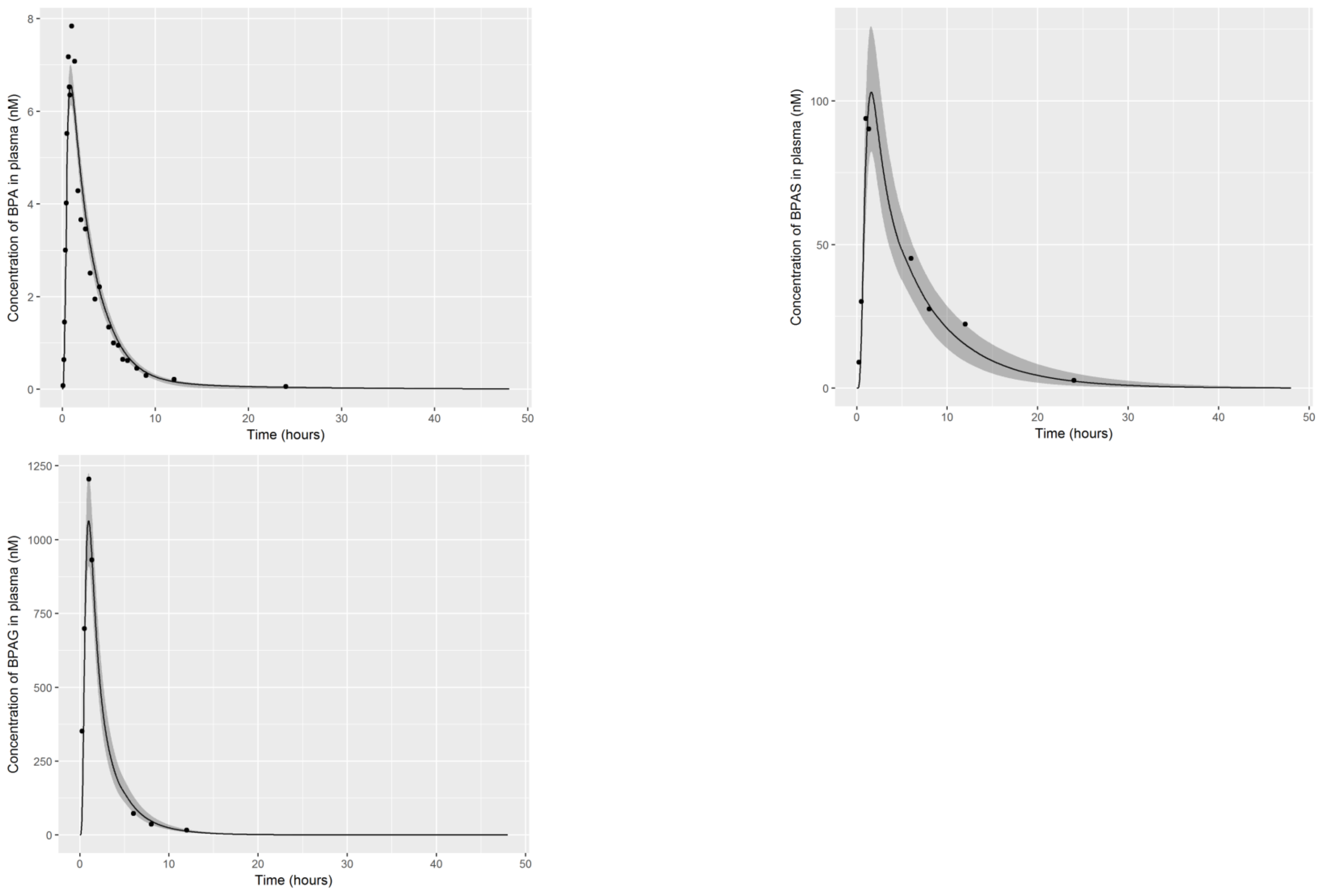

Figure S10 Individual 12

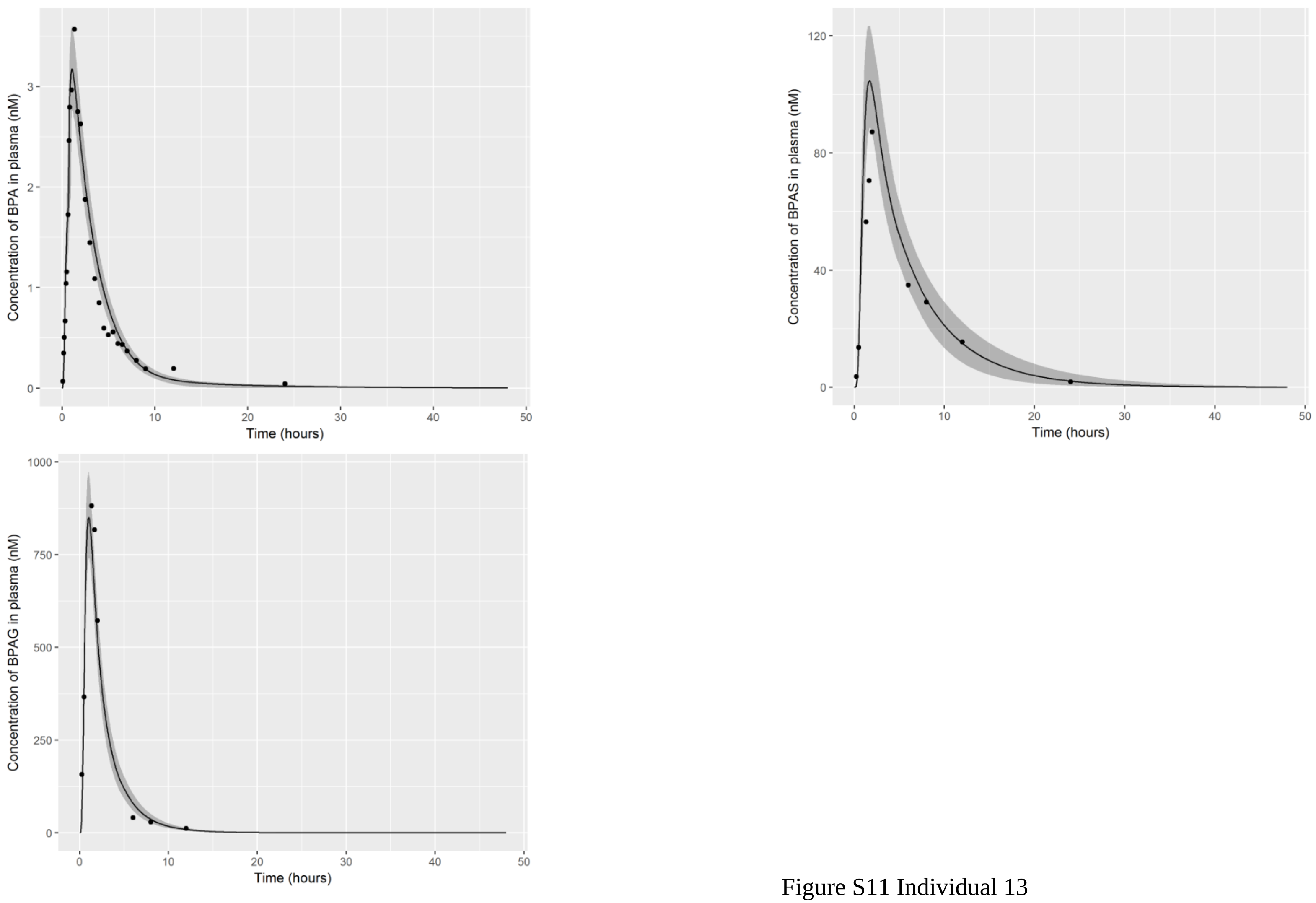

Figure S11 Individual 13

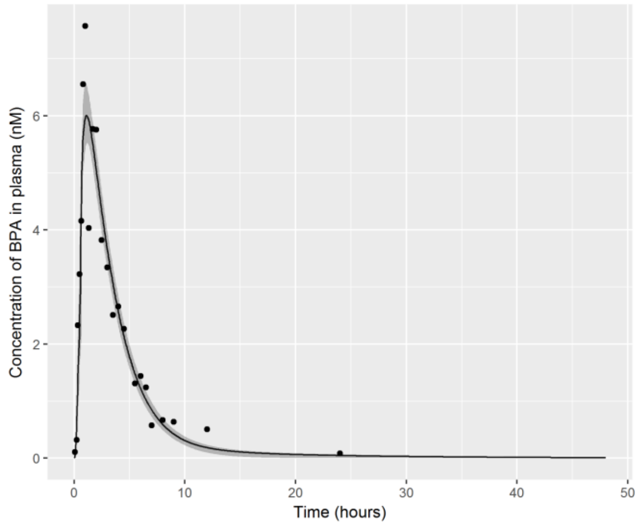

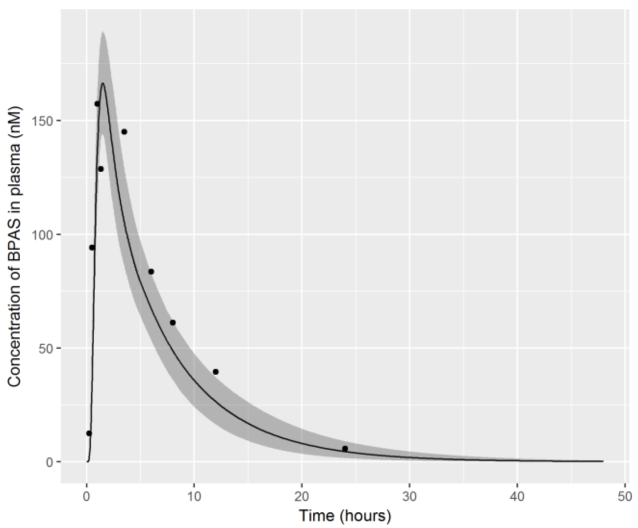

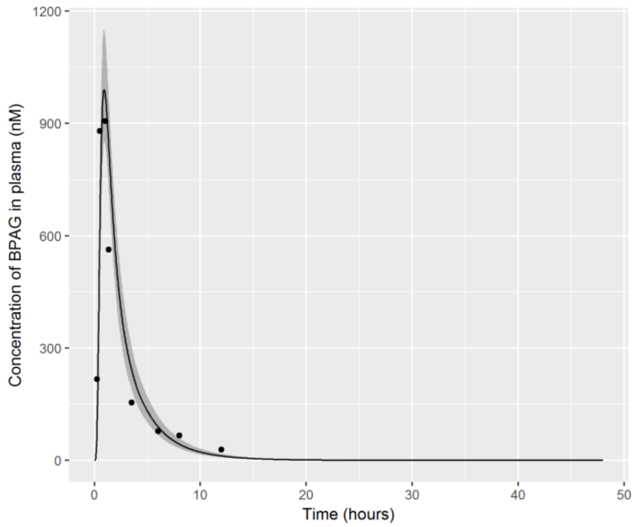

Figure S12 Individual 14

Model Code

```
# MCSim 5.x script

# *** BPA ***

# Di(2-propyl heptyl) phthalate
# George Loizou (HSL) 2020-01-16T00:00:00.0000000Z

# Compiled on: 2020-01-16T15:34:16.8794082Z

States =
{
  Afa,
  Agu1,
  Agu2,
  AMguBPAG,
  AMguBPAS,
  Ast,
  Arpd,
  Aspd,
  Ali,
  AMliBPAG,
  AMliBPAS,
  ABile,
  Alymph,
  Aplasm_BPA,
  Aplasm_BPAG,
  Aplasm_BPAS,
  ABellyH,
  AGiTractH,
  ABowel,
  ABellylymph,
  AGiTractlymph,
  AfaG,
  AstG,
  AguG,
  AliG,
  ABileG,
  ABowelG,
  AspdG,
  ArpdG,
  AfaS,
  AstS,
  AguS,
  AliS,
  ABileS,
  ABowelS,
  AspdS,
  ArpdS,
  AMBPA_REMOVED_PLASMA,
  AMBPAG_REMOVED_PLASMA,
  AMBPAS_REMOVED_PLASMA,
  AMBPA_Urine,
  AMBPAG_Urine,
  AMBPAS_Urine,
```

```
  Gutswitch,
  Lymphswitch,
  DOSESTEP
};

Outputs =
{
  Cli,
  Cfa,
  Cgu1,
  Cgu2,
  Cst,
  Clymph,
  Cspd,
  Crpd,
  mass,
  Uptake,
  reloral,
  CVfa,
  CVgu1,
  CVgu2,
  CVst,
  CVspd,
  CVli,
  CVrpd,
  CV,
  CVnmol,
  CA_BPA,
  CAT_BPA,
  CV_total_nmol,
  CVG,
  CVGnmol,
  CliG,
  CVliG,
  CguG,
  CVguG,
  CstG,
  CVstG,
  CfaG,
  CVfaG,
  CspdG,
  CVspdG,
  CrpdG,
  CVrpdG,
  CVS,
  CVSnmol,
  CliS,
  CVliS,
  CguS,
  CVguS,
  CstS,
  CVstS,
```

```
  CfaS,
  CVfaS,
  CspdS,
  CVspdS,
  CrpdS,
  CVrpdS,
  CA_BPAG,
  CAT_BPAG,
  CAT_BPAS,
  CA_BPAS,
  CV_total_BPAG_nmol,
  CV_total_BPAS_nmol,
  relBPAG,
  massBPAG,
  relBPAS,
  massBPAS,
  Curine_BPA,
  Curine_BPAG,
  Curine_BPAS,
  ORALDOSE,
  ODOSE,
  ODOSEliver,
  ODOSElymph,
  ODOSEbowel,
  Vmax_liv_BPA,
  Vmax_gut_BPAG,
  Vmax_gut_BPAS,
  Vmax_liv_BPAS,
  Ali_lag,
  AliG_lag,
  Plasma_BPA,
  Plasma_BPAG,
  Plasma_BPAS,
  Urine_BPA,
  Urine_BPAG,
  Urine_BPAS,
  Aplasmub_BPA,
  Aplasmub_BPAG,
  Aplasmub_BPAS
};

Inputs =
{
  events_Gutswitch,
  events_Lymphswitch,
  events_DOSESTEP,
  events_AMBPA_Urine,
  events_AMBPAG_Urine,
  events_AMBPAS_Urine,
};

# Parameters
```

```
# ==========
#

BW = 89;                          # body mass (kg)
MWBPA = 228.291;                  # BPA molecular mass (g/mol)
MWBPAG = 404.41;                  # BPAG molecular mass (g/mol)
MWBPAS = 308.4;                   # OH-BPAS molecular mass (g/mol)
CAE = 0.75;                       # cardiac allometric exponent
QCC = 11.22;                      # cardiac allometric constant (L/h/kg^CAE)

VT = 0.95;                        # proportion of vascularised tissue
VfaC = 0.195;                     # fractional volume
VguC = 0.067;                     # fractional volume
VstC = 0.0158;                    # fractional volume
VspdC = 0.4714;                   # fractional volume poorly perfused
VrpdC = 0.033;                    # fractional volume richly perfused
VliC = 0.0203;                    # fractional volume
VlymphC = 0.0036;                 # lymph system fractional volume
VBldC = 0.05;                     # blood fractional volume

QhepartC = 0.06;                  # hepatic artery fractional blood flow
QguC = 0.17;                      # fractional blood flow
QstC = 0.01;                      # fractional blood flow
QspdC = 0.27;                     # overall fractional blood flow to slowly perfused tissue
QrpdC = 0.42;                     # overall fractional blood flow to rapidly perfused tissue
QfaC = 0.05;                      # fractional blood flow

FracDOSELymph = 0.05;             # Fraction of dose taken into lymph
FracDOSEHep = 0.8;                # Fraction of dose taken into hepatic

FB_BPA = 0.1;                     # Fraction of BPA bound to plasma proteins 0.9998752
FB_BPAG = 0.1;                    # Fraction of BPAG bound to plasma proteins 0.9854
FB_BPAS = 0.1;                    # Fraction of BPAG bound to plasma proteins 0.9854
FracMetabG = 0.8;                 # Fraction of metabolism BPA -> BPAG
FracMetabS = 0.1;                 # Fraction of metabolism BPA -> BPAS


PORALDOSE = 0.1;                  # oral dose [mg/kg]
DRINKTIME = 0.25;                 # Drink time [h]
BELLYPERM = 0.685;                # [/h]
GIPERM1 = 5.1;                    # [/h]
GIPERM2 = 5.1;                    # [/h]
BELLYPERMlymph = 0.685;           # [/h]
GIPERMlymph = 5.1;                # [/h]
KEMAX = 10.2;                     # [Maximum emptying rate /h]
KEMIN = 0.005;                    # [Minimum emptying rate /h]
Lymphswitch = 1;
Gutswitch = 1;

MPY = 34;                         # microsomal protein yield [mg microsomal protein/g liver]
MPYgu = 3.9;                      # microsomal protein yield [mg microsomal protein/g gut]
```

Vmax_liv_BPAS_in_vitro = 73; # BPA -> BPAS hepatic female Vmax (pmol/min/mg prot) Kurebayashi et al (2010)
KM_liv_BPAS_in_vitro = 3.114; # BPA -> BPAG hepatic female KM (mg/L) (10.1 micromoles * MWBPA) Kurebayashi et al (2010)

Vmax_gut_BPAS_in_vitro = 73; # BPA -> BPAS hepatic female Vmax (pmol/min/mg prot) Kurebayashi et al (2010) scaled with gut MPYgu
KM_gut_BPAS_in_vitro = 3.114; # BPA -> BPAG hepatic female KM (mg/L) (10.1 micromoles * MWBPA) Kurebayashi et al (2010)

#BPAS_invit_gut_Clint = 1.0; # BPA -> BPAS GUT in vitro clearance (ul/min/mg prot)

Vmax_liv_BPA_in_vitro = 4255; # BPA -> BPAG hepatic female Vmax (pmol/min/mg prot) Mazur et al (2010)
KM_liv_BPA_in_vitro = 1.118; # BPA -> BPAG hepatic female KM (mg/L) (4.9 micromoles * MWBPA) Mazur et al (2010)
Vmax_gut_BPAG_in_vitro = 487; # BPA -> BPAG Gut mixed Vmax (pmol/min/mg prot) Mazur et al (2010)
KM_gut_BPAG_in_vitro = 18.29; # BPA -> BPAG Gut mixed KM (mg/L) (80.1 micromoles * MWBPA) Mazur et al (2010)

K1_BPA_REMOVED_PLASMA = 0.5; # BPA First-order elimination rate from blood [/h]
K1_BPAG_REMOVED_PLASMA = 1; # BPAG First-order elimination rate from blood [/h]
K1_BPAS_REMOVED_PLASMA = 1; # BPAS First-order elimination rate from blood [/h]

K1_BPA_Urine = 0.1; # BPA First-order elimination rate from bladder[/h]
K1_BPAG_Urine = 0.1; # BPAG First-order elimination rate from bladder [/h]
K1_BPAS_Urine = 0.1; # BPAS First-order elimination rate from bladder [/h]

K1_BPA_GUT = 0.1; # First-order elimination rate of BPA from gut into bowel [/h]
K1_BPA_LIVER = 10; # First-order elimination rate of BPA from liver into bile [/h]

K1_BPAG_GUT = 0.1; # First-order elimination rate of BPAG from gut into bowel [/h]
K1_BPAG_LIVER = 1; # First-order elimination rate of BPAG from liver into bile [/h]

K1_BPAS_GUT = 0.1; # First-order elimination rate of BPAG from gut into bowel [/h]
K1_BPAS_LIVER = 1; # First-order elimination rate of BPAG from liver into bile [/h]

K1Lymph = 0.2; # First-order elimination rate from Lyph into blood [/h]
Lymphlag = 0.01; # Lag between uptake into Lymph and emptying into blood [h]
Gutlag = 0.01; # Lag between uptake into GItract and emptying into gut [h]

Pbab = 0.73; # BPA blood:air partition coefficient
Pfab = 5.0; # BPA tissue:blood partition coefficient
Pgub = 2.8; # BPA tissue:blood partition coefficient
Pstb = 2.8; # BPA tissue:blood partition coefficient
Prpdb = 2.8; # BPA tissue:blood partition coefficient
Pspdb = 2.7; # BPA tissue:blood partition coefficient
Plib = 0.73; # BPA tissue:blood partition coefficient

PbaG = 1.39; # BPAG Red blood cells:plasma partition coefficient

```
PspdG = 2.00;                        # BPAG Slowly perfused tissue:air partition coefficient
PliG = 11.5;                         # BPAG tissue:air partition coefficient
PrpdG = 4.29;                        # BPAG Richly tissue:air partition coefficient
PfaG = 2.38;                         # BPAG Fat tissue:air partition coefficient
PstG = 3.52;                         # BPAG Stomach tissue:air partition coefficient
PguG = 3.52;                         # BPAG GI Tract tissue:blood partition coefficient

PbaS = 1.52;                         # BPAS Red blood cells:plasma partition coefficient
PspdS = 2.08;                        # BPAS Slowly perfused tissue:air partition coefficient
PliS = 12.20;                        # BPAS tissue:air partition coefficient
PrpdS = 4.56;                        # BPAS Richly tissue:air partition coefficient
PfaS = 2.63;                         # BPAS Fat tissue:air partition coefficient
PstS = 3.80;                         # BPAS Stomach tissue:air partition coefficient
PguS = 3.80;                         # BPAS GI Tract tissue:blood partition coefficient

tau = 4;                             # the required delay

Vfa = 0;
Vgu = 0;
Vst = 0;
Vspd = 0;
Vrpd = 0;
Vli = 0;
Vlymph = 0;
Qfa = 0;
Qgu = 0;
Qst = 0;
Qrpd = 0;
Qspd = 0;
Qli = 0;
QCMC = 0;
ODOSEliver = 0;
ODOSElymph = 0;
ODOSEbowel = 0;
Uptake = 0;
Vplas = 0;
VRB = 0;
Vbld = 0;
Qhepart = 0;
CA_BPA = 0;
CAT_BPA = 0;
CA_CRBC = 0;
CVnmol = 0;
CV_total_nmol = 0;
CVG = 0;
CA_BPAG = 0;
CAT_BPAG = 0;
CV_total_BPAG_nmol = 0;

# SD terms for MCMC
# ==========
#
```

```
Sigma1 = 0.1;
Sigma2 = 0.1;
Sigma3 = 0.1;
Sigma4 = 0.1;

Initialize
{
  BWc = pow(BW, CAE);                    # cardiac scaling output factor (kg)
  VplasC = 0.55 * VBldC;                 # plasma fractional volume
  HEME = 1 - (VplasC / VBldC);           # Volume of Haeme
  VRBC = HEME * VBldC;                   # Volume of red blood cells

  ## Gelman reparameterisations
  Qcci = QrpdC + QspdC + QhepartC + QfaC + QstC + QguC;
  Qrpdci = QrpdC / Qcci;
  Qspdci = QspdC / Qcci;
  Qhepartci = QhepartC / Qcci;
  Qfaci = QfaC / Qcci;
  Qstci = QstC / Qcci;
  Qguci = QguC / Qcci;

  Vti =
    (1 - VT) + VrpdC + VspdC + VliC +
    VfaC + VstC + VguC + VplasC +
    VRBC + VlymphC;
  Vguci = VguC / Vti;
  Vstci = VstC / Vti;
  Vfaci = VfaC / Vti;
  Vlici = VliC / Vti;
  Vspdci = VspdC / Vti;
  Vrpdci = VrpdC / Vti;
  Vbldci = VBldC / Vti;
  Vplasci = VplasC / Vti;
  VRBCci = VRBC / Vti;
  Vlymphci = VlymphC / Vti;

  # Volumes scaled to actual volumes
  Vfa = Vfaci * BW;                      # scaled fractional volume
  Vgu = Vguci * BW;                      # scaled fractional volume
  Vst = Vstci * BW;                      # scaled fractional volume
  Vspd = Vspdci * BW;                    # scaled fractional volume
  Vli = Vlici * BW;                      # scaled fractional volume
  Vrpd = Vrpdci * BW;                    # scaled fractional volume
  Vlymph = Vlymphci * BW;                # scaled fractional volume
  VRB = VRBCci * BW;                     # scaled red blood cell fractional volume
  Vbld = Vbldci * BW;                    # Whole blood  fractional volume
  Vplas = Vplasci * BW;                  # plasma fractional volume

  # Calculate actual blood flows from total flow and percent flows
  QC = QCC * BWc;                        # cardiac output (L/h)
  Qfa = Qfaci * QC;                      # scaled fractional blood flow
  Qgu = Qguci * QC;                      # scaled fractional blood flow
```

```
  Qst = Qstci * QC;                    # scaled fractional blood flow
  Qrpd = Qrpdci * QC;                  # scaled fractional blood flow
  Qspd = Qspdci * QC;                  # scaled fractional blood flow
  Qhepart = Qhepartci * QC;            # scaled hepatic artery fractional blood flow
  Qli = Qhepart + Qst + Qgu;           # scaled fractional blood flow
  QCMC = Qhepart + Qgu + Qst + Qfa + Qrpd + Qspd;

} # End of model initialization

Dynamics
{
  Ali_lag = CalcDelay(Ali, tau);
  AliG_lag = CalcDelay(AliG, tau);
  AliS_lag = CalcDelay(AliS, tau);

  ORALDOSE = PORALDOSE * BW;              # scaled oral dose (mg/day)
  DOSEFLOW = ORALDOSE / DRINKTIME;        # zero order uptake rate constant
  ODOSE = DOSEFLOW * DOSESTEP;            # amount absorbed (mg)
  ODOSEliver = ODOSE * FracDOSEHep;
  ODOSElymph = ODOSE * FracDOSELymph;
  ODOSEbowel = ODOSE * (1 - FracDOSEHep - FracDOSELymph);

  #ClintBPASgu = (BPAS_invit_gut_Clint/1E6) * MPYgu * Vgu * 1000 * 60;                     #
Clearance (L/h whole liver)

  #Vmax_liv_BPAG = (Vmax_liv_BPAG_in_vitro * MPY * Vli * 60 * MWBPA)/1000;                    #
Vmax scaled (mg/h whole liver)
  #Vmax_gut_BPAG = (Vmax_gut_BPAG_in_vitro * MPYgu * Vgu * 60 * MWBPA)/1000;
# Vmax scaled (mg/h whole gut)

  Vmax_liv_BPA = ((Vmax_liv_BPA_in_vitro/1E9) * MPY * Vli * 1000 * 60 * MWBPA);                 #
Vmax scaled (mg/h whole liver)
  Vmax_gut_BPAG = ((Vmax_gut_BPAG_in_vitro/1E9) * MPYgu * Vgu* 1000 * 60 * MWBPA);
# Vmax scaled (mg/h whole gut)

  Vmax_liv_BPAS =  ((Vmax_liv_BPAS_in_vitro/1E9) * MPY * Vli * 1000 * 60 * MWBPA);
# Vmax scaled (mg/h whole liver)
  Vmax_gut_BPAS =  ((Vmax_gut_BPAS_in_vitro/1E9) * MPY * Vgu * 1000 * 60 * MWBPA);
# Vmax scaled (mg/h whole liver)

#*****************************************************************************************
***!
  #            BPA   Concentrations in Compartments

#*****************************************************************************************
***!

  # cellular concentrations (mg/L)
  Cfa = Afa / Vfa;
  Cgu1 = Agu1 / Vgu;
  Cgu2 = Agu2 / Vgu;
  Cst = Ast / Vst;
```

```
  Cspd = Aspd / Vspd;
  Crpd = Arpd / Vrpd;
  Cli = Ali / Vli;
  Clymph = Alymph / Vlymph;

  # venous organ concentration (mg/L)
  CVfa = Cfa / Pfab;
  CVgu1 = Cgu1 / Pgub;
  CVgu2 = Cgu2 / Pgub;
  CVst = Cst / Pstb;
  CVspd = Cspd / Pspdb;
  CVli = Cli / Plib;
  CVrpd = Crpd / Prpdb;

  GPER = KEMAX / (1 + KEMIN * Cst);

  # venous concentration (mg/L)
  CV =
    ((CVfa * Qfa) +
    (CVrpd * Qrpd) +
    (CVspd * Qspd) +
    (CVli * Qli)) / QCMC;

  # BPA Venous concentration (nmoles/L)
  CVnmol = (CV / MWBPA) * 1000000;

  # Fraction unbound
  Aplasmub_BPA = Aplasm_BPA * (1 - FB_BPA);

  # mass in system (kg)
  mass =
    Aplasm_BPA + AMliBPAG + AMliBPAS + Ali + AMguBPAG + AMguBPAS + ABellyH + AGiTractH +
    Ast + Agu1 + Agu2 + ABowel + ABellylymph + AGiTractlymph + Alymph + Afa + Arpd +
    Aspd + ABile + AMBPA_REMOVED_PLASMA;

  Uptake = ODOSEliver + ODOSElymph + ODOSEbowel;

  # mass balance
  reloral = ((t>0) ? mass / (ORALDOSE + 1e-10) : 1);
  #reloral = ((t>0) ? mass / (Uptake + 1e-10) : 1);

  CA_BPA = Aplasmub_BPA / (Vplas);    # Arterial unbound concentration (ng/L)

#*****************************************************************************************
***!
  #              BPAG  Concentrations in Compartments

#*****************************************************************************************
***!

  # cellular concentrations (mg/L)
  CguG = AguG / Vgu;
```

```
  CstG = AstG / Vst;
  CfaG = AfaG / Vfa;
  CliG = AliG / Vli;
  CspdG = AspdG / Vspd;
  CrpdG = ArpdG / Vrpd;

  # venous organ concentration (mg/L)
  CVguG = CguG / PguG;
  CVstG = CstG / PstG;
  CVfaG = CfaG / PfaG;
  CVliG = CliG / PliG;
  CVspdG = CspdG / PspdG;
  CVrpdG = CrpdG / PrpdG;

  CVG = ((CVliG * Qli) + (CVfaG * Qfa) + (CVspdG * Qspd) + (CVrpdG * Qrpd)) / QCMC;

  # BPAG Venous concentration (nmoles/L)
  CVGnmol = (CVG / MWBPAG) * 1000000;

  #unbound model
  Aplasmub_BPAG = Aplasm_BPAG * (1 - FB_BPAG);
  CA_BPAG = Aplasmub_BPAG / (Vplas);


  # mass in system (kg)
  massBPAG = AliG + AguG + AstG + AfaG + AspdG + ArpdG + ABileG + Aplasm_BPAG + ABowelG +
AMBPAG_REMOVED_PLASMA;

  # BPAG mass balance
  relBPAG = ((t>0) ? massBPAG / (AMliBPAG + AMguBPAG + 1e-10) : 1);
  #relBPAG = ((t>0) ? massBPAG / (AMguBPAG + 1e-10) : 1);
  #relBPAG = ((t>0) ? massBPAG / (AMliBPAG + 1e-10) : 1);

#**************************************************************************************
***!
  #              BPAS  Concentrations in Compartments

#**************************************************************************************
***!

  # cellular concentrations (mg/L)
  CguS = AguS / Vgu;
  CstS = AstS / Vst;
  CfaS = AfaS / Vfa;
  CliS = AliS / Vli;
  CspdS = AspdS / Vspd;
  CrpdS = ArpdS / Vrpd;

  # venous organ concentration (mg/L)
  CVguS = CguS / PguS;
  CVstS = CstS / PstS;
  CVfaS = CfaS / PfaS;
```

```
  CVliS = CliS / PliS;
  CVspdS = CspdS / PspdS;
  CVrpdS = CrpdS / PrpdS;

  CVS = ((CVliS * Qli) + (CVfaS * Qfa) + (CVspdS * Qspd) + (CVrpdS * Qrpd)) / QCMC;
  # BPAS Venous concentration (nmoles/L)
  CVSnmol = (CVS / MWBPAS) * 1000000;

  #unbound model
  Aplasmub_BPAS = Aplasm_BPAS * (1 - FB_BPAS);
  CA_BPAS = Aplasmub_BPAS / (Vplas);


  # mass in system (kg)
  massBPAS = AliS + AguS + AstS + AfaS + AspdS + ArpdS + ABileS + Aplasm_BPAS + ABowelS +
AMBPAS_REMOVED_PLASMA;

  # BPAS mass balance
  relBPAS = ((t>0) ? massBPAS / (AMliBPAS + AMguBPAS + 1e-10) : 1);


#******************************************************************************************
***!
  #               BPA   Differential Equations

#******************************************************************************************
***!

  dt (Gutswitch) = 0;
  dt (Lymphswitch) = 0;
  dt (DOSESTEP) = 0;

  dt (Aplasm_BPA) = QCMC * (CV - CA_BPA) +
                Lymphswitch * Alymph * K1Lymph
                - (K1_BPA_REMOVED_PLASMA*Aplasm_BPA);             # Amount in plasma (mg)


  #dt (AMliBPAG) = ((CVli * Vmax_liv_BPA) / (CVli + KM_liv_BPA_in_vitro)) * FracMetabG;
# Amount of hepatic metabolism BPA -> BPAG (mg/h/kg)

  #dt (AMliBPAS) = ((CVli * Vmax_liv_BPA) / (CVli + KM_liv_BPA_in_vitro)) * (1-(FracMetabG);
# Amount of hepatic metabolism BPA -> BPAS (mg/h/kg)

  dt (AMliBPAG) = ((CVli * Vmax_liv_BPA) / (CVli + KM_liv_BPA_in_vitro));                        #
Amount of hepatic metabolism BPA -> BPAG (mg/h/kg)

  dt (AMliBPAS) = ((CVli * Vmax_liv_BPAS_in_vitro) / (CVli + KM_liv_BPAS_in_vitro));
# Amount of hepatic metabolism BPA -> BPAS (mg/h/kg)


  dt (Ali) = (Qhepart * CA_BPA) + (Qst * CVst) + (Qgu * CVgu2) - (Qli * CVli) -
            (dt (AMliBPAG) + dt (AMliBPAS))  -
```

```
        (K1_BPA_LIVER * Ali);              # Amount in liver (mg)

  dt (ABile) = K1_BPA_LIVER * (Ali - Ali_lag);                                    # Amount in Bile (mg)

  dt (AMguBPAG) = ((CVgu1 * Vmax_gut_BPAG) / (CVgu1 + KM_gut_BPAG_in_vitro));
# Amount of gut metabolism BPA -> BPAG  (mg/h/kg)

  dt (AMguBPAS) = ((CVgu1 * Vmax_gut_BPAS) / (CVgu1 + KM_gut_BPAS_in_vitro));
# Amount of gut metabolism BPA -> BPAS  (mg/h/kg)


  #dt (AMguBPAS) = ((Qgu * ClintBPASgu) / (Qgu + ClintBPASgu / Pbab)) * CVgu1;
# Amount of gut metabolism BPA -> BPAS  (mg/h/kg)


  dt (ABellyH) = (ODOSEliver) - (GPER * ABellyH) - (BELLYPERM * ABellyH);
# Amount of in stomach compartment (mg/h/kg)
  dt (AGiTractH) = (GPER * ABellyH) - (GIPERM1 * AGiTractH);                      # Amount of in GI Tract compartment (mg/h/kg)
  dt (Ast) = Qst * (CA_BPA - CVst) + BELLYPERM * ABellyH;                         # Amount of in STOMACH compartment (mg/h/kg)

  dt (Agu1) = (GIPERM1 * AGiTractH) -
        (dt (AMguBPAG) + dt (AMguBPAS)) -
        (Gutswitch * GIPERM2 * Agu1);

  dt (Agu2) = Qgu * (CA_BPA - CVgu2) +
        (Gutswitch * GIPERM2 * Agu1) -
        (Lymphswitch * K1_BPA_GUT * Agu2) +
        (K1_BPA_LIVER * Ali_lag);

  dt (ABowel) = ODOSEbowel + (Lymphswitch * K1_BPA_GUT * Agu2);                   # Elimination rate from gut into faeces (mg)

  dt (ABellylymph) = (ODOSElymph) -(GPER * ABellylymph) - (BELLYPERMlymph * ABellylymph);

  dt (AGiTractlymph) = (GPER * ABellylymph) - (GIPERMlymph * AGiTractlymph);
# BPA rate of uptake in lymph compartment  (mg/h/kg)

  dt (Alymph) =                                                 # Amount in lymph
    (BELLYPERMlymph * ABellylymph) +
    (GIPERMlymph * AGiTractlymph) -
    Lymphswitch * Alymph * K1Lymph;

  dt (Afa) = Qfa * (CA_BPA - CVfa);            # cellular compartment derivative (mg/h/kg)
  dt (Arpd) = Qrpd * (CA_BPA - CVrpd);         # cellular compartment derivative (mg/h/kg)
  dt (Aspd) = Qspd * (CA_BPA - CVspd);         # cellular compartment derivative (mg/h/kg)


#*****************************************************************************************
***!
```

```
 #              BPAG   Differential Equations

#*****************************************************************************************
***!

 dt (AliG) =
   (Qhepart * CA_BPAG) +
   (Qst * CVstG) +
   (Qgu * CVguG) -
   (Qli * CVliG) +
   dt (AMliBPAG) -
   (K1_BPAG_LIVER * AliG);


 dt (ABileG) = K1_BPAG_LIVER * (AliG - AliG_lag);
 dt (AstG) = Qst * (CA_BPAG - CVstG);

 dt (AguG) =
   Qgu * (CA_BPAG - CVguG) +
   dt (AMguBPAG) -
   (Lymphswitch * K1_BPAG_GUT * AguG) +
   (K1_BPAG_LIVER * AliG_lag);

 dt (ABowelG) = K1_BPAG_GUT * AguG;

 dt (AfaG) = Qfa * (CA_BPAG - CVfaG);
 dt (AspdG) = Qspd * (CA_BPAG - CVspdG);
 dt (ArpdG) = Qrpd * (CA_BPAG - CVrpdG);

 #unbound model

 dt (Aplasm_BPAG) = QCMC * (CVG - CA_BPAG) - (K1_BPAG_REMOVED_PLASMA*Aplasm_BPAG);


#***************************S*************************************************************
****!
 #              BPAS   Differential Equations

#*****************************************************************************************
***!

 dt (AliS) =
   (Qhepart * CA_BPAS) +
   (Qst * CVstS) +
   (Qgu * CVguS) -
   (Qli * CVliS) +
   dt (AMliBPAS) -
   (K1_BPAS_LIVER * AliS);

 dt (ABileS) = K1_BPAS_LIVER * (AliS - AliS_lag);
 dt (AstS) = Qst * (CA_BPAS - CVstS);
```

```
  dt (AguS) =
    Qgu * (CA_BPAS - CVguS) +
    dt (AMguBPAS) -
    (Lymphswitch * K1_BPAS_GUT * AguS) +
    (K1_BPAS_LIVER * AliS_lag);

  dt (ABowelS) = K1_BPAS_GUT * AguS;

  dt (AfaS) = Qfa * (CA_BPAS - CVfaS);
  dt (AspdS) = Qspd * (CA_BPAS - CVspdS);
  dt (ArpdS) = Qrpd * (CA_BPAS - CVrpdS);

  #unbound model

  dt (Aplasm_BPAS) = QCMC * (CVS - CA_BPAS) - (K1_BPAS_REMOVED_PLASMA*Aplasm_BPAS);

#*********************************************************************************************
***!
  #            BPAG Urinary excretion

#*********************************************************************************************
***!

  dt (AMBPA_REMOVED_PLASMA) = K1_BPA_REMOVED_PLASMA*Aplasm_BPA;   # Removal of
BPA from liver
  dt (AMBPAG_REMOVED_PLASMA) = K1_BPAG_REMOVED_PLASMA*Aplasm_BPAG; # Removal of
BPAG from liver
  dt (AMBPAS_REMOVED_PLASMA) = K1_BPAS_REMOVED_PLASMA*Aplasm_BPAS; # Removal of
BPAS from liver


  dt (AMBPA_Urine)  = dt (AMBPA_REMOVED_PLASMA)  - K1_BPA_Urine * AMBPA_Urine;       #Rate
of change in bladder
  dt (AMBPAG_Urine) = dt (AMBPAG_REMOVED_PLASMA) - K1_BPAG_Urine * AMBPAG_Urine;
#Rate of change in bladder
  dt (AMBPAS_Urine) = dt (AMBPAS_REMOVED_PLASMA) - K1_BPAS_Urine * AMBPAS_Urine;     #
Rate of change in bladder

  Curine_BPA =   (AMBPA_Urine/MWBPA*1e6);                          # Concentration of BPA in urine
(nmol)

  Curine_BPAG =  (AMBPAG_Urine/MWBPAG*1e6)*(MWBPAG/MWBPA);                    # Concentration
of BPA in urine (nmol)

  Curine_BPAS = (AMBPAS_Urine/MWBPAS*1e6)*(MWBPAS/MWBPA);                    # Concentration
of BPA in urine (nmol)

}

#These are new addition. Also specified in final four lines of 'outputs'
CalcOutputs
{
```

```
  CAT_BPA = Aplasm_BPA/(MWBPA*Vplas)*1e6;                  # Total concentration in plasma (nmol/l)
  CV_total_nmol = (Aplasm_BPA)/(MWBPA*Vbld)*1e6;      # Total concentration in blood (nmol/l)

  #CAT_BPAG = Aplasm_BPAG/(MWBPAG*Vplas)*1e6;
  CAT_BPAG = Aplasm_BPAG/(MWBPAG*Vplas)*1e6*(MWBPAG/MWBPA);

  CV_total_BPAG_nmol = (Aplasm_BPAG)/(MWBPAG*Vbld)*1e6*(MWBPAG/MWBPA);

  CAT_BPAS = Aplasm_BPAS/(MWBPAS*Vplas)*1e6*(MWBPAS/MWBPA);
  CV_total_BPAS_nmol = (Aplasm_BPAS)/(MWBPAS*Vbld)*1e6*(MWBPAS/MWBPA);

  Plasma_BPA = (Aplasm_BPA)/(Vbld);
  Plasma_BPAG = (Aplasm_BPAG)/(Vbld);
  Plasma_BPAS = (Aplasm_BPAS)/(Vbld);

  Urine_BPA =   Curine_BPA;
  Urine_BPAG =  Curine_BPAG;
  Urine_BPAS =  Curine_BPAS;
}

End.
```

References

Brown RP, Delp MD, Lindstedt SL, Rhomberg LR, Beliles RP. 1997. Physiological parameter values for physiologically based pharmacokinetic models. Toxicol Ind Health. 13(4):407-484.

ICRP. 2002. Basic anatomical and physiological data for use in radiological protection: Reference values. Pergamon.

Loizou G, McNally K, Dorne J-LCM, Hogg A. 2021. Derivation of a human in vivo benchmark dose for perfluorooctanoic acid from toxcast in vitro concentration–response data using a computational workflow for probabilistic quantitative in vitro to in vivo extrapolation. Frontiers in Pharmacology. 12(570).

Loizou GD, McNally K, Jones K, Cocker J. 2015. The application of global sensitivity analysis in the development of a physiologically based pharmacokinetic model for m-xylene and ethanol co-exposure in humans. Front Pharmacol. 6:1-19.

Mazur CS, Kenneke JF, Hess-Wilson JK, Lipscomb JC. 2010. Differences between human and rat intestinal and hepatic bisphenol a glucuronidation and the influence of alamethicin on in vitro kinetic measurements. Drug metabolism and disposition. 38(12):2232-2238.

McNally K, Cotton R, Cocker J, Jones K, Bartels M, Rick D, Price P, Loizou G. 2012. Reconstruction of exposure to m-xylene from human biomonitoring data using pbpk modelling, bayesian inference, and markov chain monte carlo simulation. Journal of Toxicology. 2012:18.

McNally K, Cotton R, Hogg A, Loizou G. 2014. Popgen: A virtual human population generator. Toxicology. 315(0):70-85.

McNally K, Cotton R, Loizou G. 2011. A workflow for global sensitivity analysis of pbpk models. Frontiers in Pharmacology: Predictive Toxicity. 2:Article 31, 31-21.

Schmitt W. 2008. General approach for the calculation of tissue to plasma partition coefficients. Toxicol In Vitro. 22(2):457-467.

Thayer KA, Doerge DR, Hunt D, Schurman SH, Twaddle NC, Churchwell MI, Garantziotis S, Kissling GE, Easterling MR, Bucher JR et al. 2015. Pharmacokinetics of bisphenol a in humans following a single oral administration. Environment International. 83:107-115.
